# Deep learning-driven optimization and predictive modeling of LASER beam machining for XG3 steel

**DOI:** 10.1038/s41598-025-34323-6

**Published:** 2026-01-02

**Authors:** Adithya Hegde, Raviraj Shetty, Gururaj Bolar, V Balaji

**Affiliations:** 1 Department of Mechanical and Industrial Engineering, Manipal Institute of Technology, Manipal Academy of Higher Education, Manipal, 576104 India; 2 Department of Robotics and Artificial Intelligence, Mangalore Institute of Technology and Engineering, Moodabidre, Mangalore, 574225 Karnataka India; 3 Department of Aerospace Engineering, Indian Institute of Science, Bengaluru, India

**Keywords:** LASER, XG3 steel, Optimization, Surface roughness, TOPSIS, ANOVA, RSM, BPANN, Engineering, Mechanical engineering

## Abstract

LASER Beam Machining (LBM) has emerged as a highly precise and non-contact thermal machining process, widely adopted for cutting complex geometries in advanced engineering materials. Its ability to machine difficult-to-cut alloys with minimal mechanical stress makes it particularly suitable for aerospace and defense components. This paper presents an experimental investigation and multi-objective optimization of LASER Beam Machining (LBM) for XG3 steel, a high-performance alloy used in aerospace and defense applications. The study evaluates the impact of four process parameters i.e. cutting speed (8, 10, 12 m/min), gas pressure (0.5, 0.7, 0.9 Bar), focus point (2, 4, 6 mm), and depth of cut (3, 6, 9 mm) on four output responses: surface roughness, machining time, surface hardness, and burr thickness. Experiments were conducted using a Taguchi L_27_ orthogonal array on three distinct hole geometries: circular, triangular, and square. Analysis of Variance (ANOVA) revealed that cutting speed was the most dominant factor, contributing over 82% to the variation in surface roughness, 74% for machining time, 81% for surface hardness, and 84% for burr thickness. The interaction between cutting speed and depth of cut was also found to be statistically significant. For single-objective optimization, the ideal parameters to minimize surface roughness were a cutting speed of 12 m/min, gas pressure of 0.5 bar, focus point of 2 mm, and depth of cut of 3 mm. Multi-objective optimization using a Genetic Algorithm (MOGA) generated Pareto fronts to identify balanced trade-off solutions; for a circular profile, this resulted in surface roughness values of 1.10–1.16 μm and machining times of 2.44–2.52 s. Furthermore, two predictive models, Response Surface Methodology (RSM) and a Back-Propagation Artificial Neural Network (BPANN), were developed. Comparative analysis showed the BPANN model was significantly more accurate, with regression coefficients (R) exceeding 0.999 and Mean Absolute Percentage Error (MAPE) values of 1.48% for surface roughness and 0.72% for surface hardness, confirming its superior predictive capability.

## Introduction

LASER Beam Machining (LBM) is an advanced non-contact thermal energy-based material removal process that uses a high-intensity beam of monochromatic and coherent photons to melt, vaporize, or thermally fracture material from the surface of a workpiece. The beam is focused using lenses or fiber optics into a small, concentrated spot that generates an intense localized heat source. This heat input results in rapid melting and, in many cases, partial or complete vaporization of the material, depending on the laser type, pulse characteristics, and interaction time. LBM has become a key technique in precision manufacturing, especially in sectors like aerospace, automotive, biomedical, and electronics, where the need for intricate geometries and superior surface integrity is critical.

**Fig. 1:**
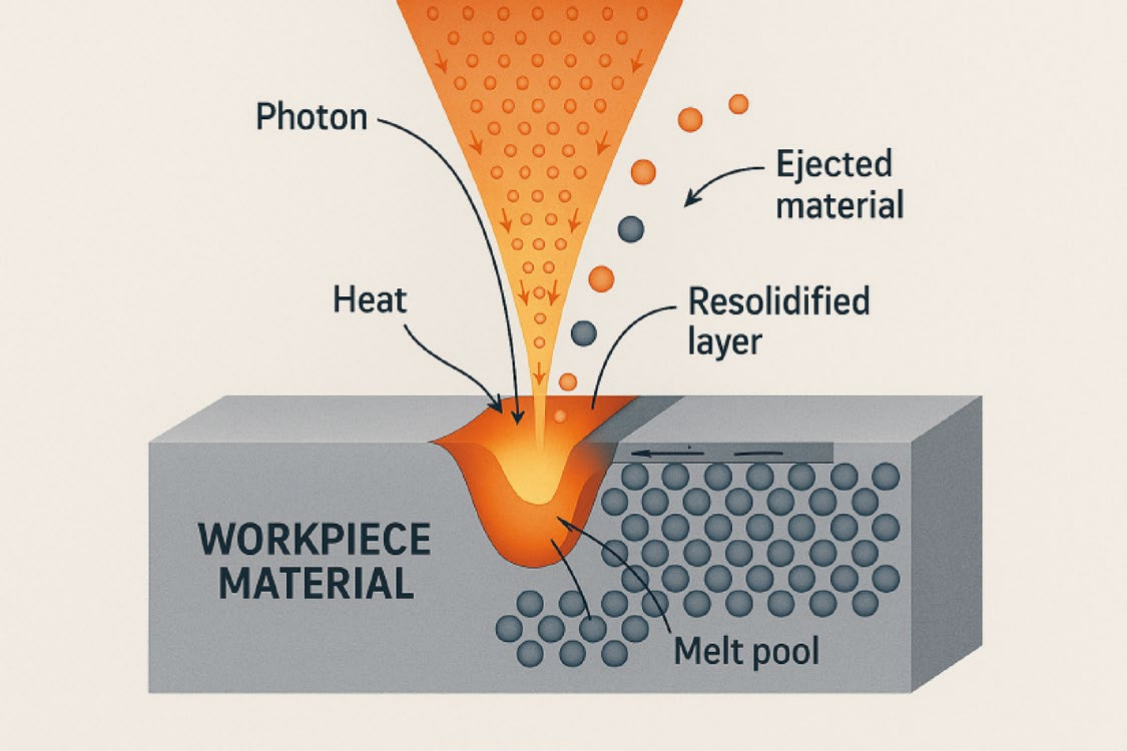
Material removal mechanism in LASER beam machining.

The laser action begins with the generation of photons by exciting the lasing medium using an external energy source, such as electrical discharge, optical pumping, or chemical reaction (Fig. 1). The photons stimulate the emission of other photons in a cascade process inside the laser cavity, and the resultant beam is then focused on the work surface. The high photon density at the focal point leads to intense absorption of energy by the surface atoms. This energy absorption increases the temperature of the material at the surface beyond its melting and boiling points. As a result, a localized molten pool is formed, which either vaporizes or is expelled due to recoil pressure, leaving behind a cavity or cut.

The material removal mechanism in LBM includes complex thermal and fluidic phenomena. In continuous wave lasers, sustained heating leads to progressive melting and vaporization, while in pulsed lasers, short bursts of high energy result in rapid thermal shocks and spallation^1^. Following material removal, the remaining molten layer cools and resolidifies. This resolidification forms a recast layer, which can be detrimental to surface finish and dimensional accuracy^2^. The thickness and morphology of this layer depend heavily on the energy input, cooling rate, and thermal diffusivity of the workpiece material^3^. In high-strength alloys and hard materials, resolidification may also introduce residual stresses and microcracks, which need to be minimized through optimized process control^4^.

LASER Beam Machining offers several advantages such as high precision, minimal mechanical load, and the ability to process difficult-to-machine materials. However, these benefits can only be fully realized when the process parameters are carefully selected and optimized. The primary input parameters in LBM include laser power, cutting or scanning speed, pulse duration and frequency (for pulsed lasers), focal position, beam diameter, and assist gas pressure and type. Each of these inputs significantly influences the machining performance and output characteristics^5–7^.

For example, laser power governs the total energy delivered to the material. Higher power typically leads to deeper penetration and faster machining, but can also increase surface roughness due to uncontrolled melting or excessive recast. Cutting speed determines the interaction time between the beam and material^8^. A higher speed reduces thermal damage and recast layer thickness but may result in incomplete cutting. The focal position affects energy density; focusing below the surface can promote deeper cuts, while surface-level focusing helps in achieving finer features. Assist gas is another critical factor. Inert gases like nitrogen or argon are used to prevent oxidation, while reactive gases like oxygen can enhance cutting efficiency through exothermic reactions^9^.

The key output responses in LBM include surface roughness, machining time, material removal rate, heat-affected zone, burr formation, and surface hardness^10^. Surface roughness is primarily affected by the stability of the molten pool and the dynamics of melt ejection. Machining time is influenced by power density and scanning strategy. Burr thickness and hardness changes depend on thermal gradients and rapid cooling, while the presence of a recast layer affects dimensional accuracy and fatigue life^11^.

To improve the quality of machining and achieve multiple performance targets simultaneously, optimization of LBM parameters is essential. Numerous researchers have adopted statistical and computational techniques to identify optimal parameter combinations^12,13^. The Taguchi method is one of the most widely used statistical tools for LBM process optimization. It uses orthogonal arrays to design a minimal set of experiments and applies signal-to-noise ratios to determine the influence of parameters on output responses. This method is particularly effective for single-objective optimization and sensitivity analysis^14^.

However, when multiple objectives are involved, such as minimizing surface roughness while maximizing material removal rate, more advanced techniques are required. Genetic Algorithm (GA) is a popular evolutionary approach that mimics natural selection to search for optimal solutions. It uses operations such as selection, crossover, and mutation to evolve a population of solutions over generations. GA is robust in handling complex, nonlinear, and multi-modal objective functions^15^.

Other optimization methods include Grey Relational Analysis (GRA), which converts multiple responses into a single grey relational grade for ranking; Artificial Neural Networks (ANN), which model complex interactions among variables; and hybrid methods like ANFIS (Adaptive Neuro-Fuzzy Inference System), which combine the learning capabilities of neural networks with the decision logic of fuzzy systems. These AI-driven models are often trained using experimental data and then coupled with metaheuristic algorithms such as Particle Swarm Optimization (PSO), Simulated Annealing (SA), or Whale Optimization Algorithm (WOA) to identify the best combination of process parameters.

As the demand for precision components with superior surface integrity continues to grow across industries, researchers have focused on improving the LBM process through systematic experimentation and parameter optimization. Several investigations have been carried out on different materials to understand how laser parameters affect output responses such as surface roughness, machining time, material removal rate, burr thickness, and microstructural quality.

For instance, Eaysin et al.^16^ investigated laser beam machining of AISI P20 mold steel using a pulsed fiber laser. In their experiments the P20 steel was cut by a Bodor F3015 fiber laser into 1.5 × 1.5 cm squares in 27 runs. They employed a Taguchi L_27_ design (set up in Minitab) to vary cutting speed, assist gas pressure and laser power, while measuring surface roughness (Ra), kerf width and heat-affected zone. An adaptive neuro-fuzzy inference system (ANFIS) was trained on the data to predict Ra and kerf; the model was then exhaustively searched to find the parameter set that minimized both metrics. The authors used a “smaller-is-better” signal-to-noise ratio for both Ra and kerf, and the brute-force search agreed with the Taguchi optimum. They found that 1 m/min cutting speed, 2 bar gas and 1.8 kW power gave the lowest Ra, while 1 m/min, 2 bar and 1.9 kW produced the narrowest kerf (0.84 mm). At this setting the surface roughness was about 4.48 μm. Microstructural examination of the cut edges confirmed the differences between low- and high-Ra trials. The study highlighted that cutting speed was the most significant factor, and demonstrated the effectiveness of combining Taguchi design with ANFIS modeling for optimizing multiple LBM outputs simultaneously. For completeness, the heat-affected zone was also recorded, but the optimization focused on Ra and kerf. Their hybrid statistical–computational framework successfully identified a realistic tradeoff between productivity and quality in P20 steel cutting.

Similarly, Rajamani et al.^17^ studied Nd: YAG laser cutting of Hastelloy C276, a nickel-based superalloy, using an ANFIS model coupled with a whale optimization algorithm (WOA). They ran multiple cutting trials on 0.5 mm thick Hastelloy sheets to train the model. The authors reported a maximum material removal rate (MRR) of 236.98 mg/min with a kerf taper of 1.135° under the best conditions, while the optimized setting also yielded a surface roughness on the order of 1.109 μm. The ANFIS–WOA approach was verified by confirmatory experiments, demonstrating accurate predictions. Cuts made at the optimal parameters exhibited clean, defect-free edges. Notably, ANFIS is a neuro-fuzzy predictor that captures nonlinear relations and WOA is an evolutionary search that efficiently seeks global optima, so their combination enabled efficient convergence to the best settings. The authors noted that higher assist-gas pressure led to straighter cuts and lower taper, aligning with the general finding that gas pressure dominates kerf quality. Overall, this study showed that the hybrid ANFIS–WOA optimization can balance throughput and finish, automatically converging to superior laser-cutting conditions for this difficult-to-machine alloy. They found that this intelligent optimization outperformed manual tuning, highlighting the value of data-driven global search in LBM parameter selection.

Another team, Alsoruji et al.^18^ applied a CO₂ laser for drilling holes in Inconel 718, another Ni-based superalloy. They used a 2 kW continuous-wave CO₂ laser with oxygen assist to drill into a 14 mm thick plate, following a Taguchi-designed set of trials. Grey relational analysis combined surface finish, MRR and taper into a single performance measure. The TGRA analysis identified a laser power of 2 kW, nozzle distance 0.7 mm, focal offset + 2 mm and gas pressure 3 bar as the best setting. Under that combination the holes exhibited minimal taper and the best surface finish while maintaining a high MRR. Analysis of variance showed that assist gas pressure had the dominant effect on performance. The authors emphasized that achieving high cut quality in Inconel requires effective assist gas and focus control. This work confirmed that Taguchi–Grey relational optimization can successfully find parameter sets that balance the tradeoff between fast drilling and smooth hole quality. In practice, Grey relational analysis normalized the multiple outputs so that Ra, taper and MRR could be ranked as a single grey grade, greatly simplifying multi-response optimization.

Muthuramalingam et al.^19^ performed a CO₂ laser drilling study on a Ti-6Al-4 V titanium alloy. They used a pulsed 2 kW CO₂ laser to drill through 8 mm thick sheet, varying laser power, nozzle standoff, focal position and gas pressure in a Taguchi L9 design. Grey relational grades combined surface roughness and taper for each trial. Analysis of variance confirmed laser power was the most influential factor, due to plasma energy effects. The optimal condition (3 kW power, 1.5 mm standoff, 2 mm focal shift, 2 bar) produced the lowest Ra and taper. In particular, the high-power setting with close nozzle spacing produced finer plasma craters and reduced particle adhesion on the Ti surface. If power was set too low, holes were incomplete and finish suffered, illustrating the narrow effective window. Overall, the results reinforce that TGRA can effectively balance multiple objectives, and that increasing laser energy (within reason) can improve surface finish in pulsed CO₂ drilling of titanium. The study used Mitutoyo profilometry to measure Ra and careful cross-sectional imaging for taper, and confirmed that multi-response Taguchi–grey optimization provided a clear single optimum.

Min et al.^20^ examined groove cutting in a titanium alloy using a low-power fiber-coupled diode laser. In their experiments the Ti samples (6 mm thick) were grooved with a 20 W diode laser over 50 runs from a fractional factorial design. Kerf width and Ra were measured and used to train an ANFIS model with five inputs (laser power, standoff, feed rate, duty cycle, frequency). The ANFIS model was tuned with a genetic algorithm (GA) over 100 iterations to improve its predictive accuracy: it achieved R²≈0.99 on training data and 0.98 on testing data. For multi-objective optimization, a grey-relational approach combined kerf and roughness into a single index. The result was an optimum set (20 W power, 22 mm standoff, 300 mm/min feed, 85% duty, 18 kHz) that minimized both kerf width and Ra. In effect, the GA–ANFIS model accurately captured the complex interactions of all five parameters, and the grey grade provided an efficient way to pick a balanced trade-off solution. The authors noted that GA tuning converged faster than a simple grid search, and grey analysis allowed equal weighting of both quality measures. This hybrid approach proved capable of finding optimal diode-laser settings that cut cleanly with minimal recast, demonstrating the power of combining AI modeling with evolutionary search for laser machining.

Praveen et al.^21^ investigated fiber-laser cutting of a high-strength low-alloy (HSLA) steel. They used a 2 kW fiber laser on 4 mm thick HSLA plates, employing Taguchi’s L18 design to vary parameters like power, cutting speed and assist gas pressure. Surface roughness and kerf width were measured by profilometry on each cut. Analysis of variance identified laser power and cutting speed as the most significant factors. The multiple responses were combined into a single score via the MOORA (Multi-Objective Optimization on the basis of Ratio Analysis) method, which normalized and weighted the Ra and kerf values to rank each run. The top-ranked parameter set was then tested and produced a much smoother cut than baseline conditions. Thus, this work demonstrated that the MOORA approach can efficiently integrate conflicting objectives: the chosen settings gave a simulated ~ 20% reduction in Ra and kerf compared to average runs. The authors remarked that MOORA required minimal computation and gave a clear ranking, making it a practical multi-criteria tool for experimental LBM optimization.

Demir et al.^22^ performed multi-objective optimization of fiber-laser cutting parameters on a 20 mm thick AISI 304 L stainless-steel plate. They used Taguchi’s design to vary cutting speed, focal position, pulse frequency and duty cycle, while monitoring surface roughness and kerf width. ANOVA showed that duty cycle (49% contribution) and pulse frequency (31%) were the most influential factors. Grey relational analysis was then used to merge Ra and kerf into a single optimization score. The optimum condition was 310 mm/min speed, 11 mm focal offset, 105 Hz frequency and 60% duty cycle, which yielded the minimal kerf and roughness in their trials. Surface profiles confirmed that cut edges under these settings were smooth and vertical. This study illustrates the applicability of Taguchi–grey optimization to pulsed fiber-LBM of thick stainless steel, highlighting pulse parameters (duty/frequency) as key variables. In their case, a shorter pulse period and moderate duty cycle produced the best surface finish, showing how oxide layer and material properties were effectively managed.

Taken together, these experimental investigations illustrate the utility of combining statistical DOE with intelligent search methods for LBM process optimization. Commonly, a Taguchi array is first used to structure the experiments. Then modeling tools such as ANFIS or neural networks capture the nonlinear process behavior from the data. Multi-response techniques like Grey relational analysis or MOORA convert multiple objectives (e.g. minimize Ra and kerf) into a single score for optimization. For example, Eaysin et al. used Taguchi–ANFIS with brute-force search, Alsoruji and Demir employed Taguchi–Grey analysis, Min and Rajamani used hybrid ANFIS tuned by genetic/whale algorithms, and Praveen combined Taguchi with MOORA. This variety shows the common pattern: Taguchi for experiment planning, AI models for prediction, grey or decision metrics for multi-criteria synthesis, and metaheuristic searches to fine-tune the result. These hybrid strategies effectively capture nonlinear parameter effects and balance multiple quality measures within one framework. Each study’s chosen method succeeded in finding improved LBM settings, significantly enhancing surface finish or cutting speed over nominal conditions.

The present study focuses on the experimental investigation and multi-objective optimization of LASER Beam Machining (LBM) for the processing of XG3 steel, a high-performance structural alloy developed for critical applications such as aerospace and defense components, where dimensional precision, thermal resistance, and mechanical integrity are essential. Unlike traditional grades, XG3 steel exhibits enhanced strength-to-weight ratio, improved corrosion resistance, and superior thermal stability, making it a challenging material to machine using conventional methods. The complex thermal and mechanical behavior of XG3 under laser irradiation has not been adequately explored in the existing literature, particularly with regard to feature geometries and burr formation in intricate profiles. While many earlier studies have concentrated on simple cylindrical cuts or linear grooves, this work introduces a comprehensive comparison of LBM response across three different hole geometries—circular, triangular, and rectangular—using the same material and experimental conditions. This allows for a deeper understanding of how geometric complexity interacts with process parameters to influence key machining outcomes. This study varies four critical input parameters; cutting speed, gas pressure, focus point, and depth of cut; each selected based on a preliminary screening and literature guidance. These parameters are known to directly affect energy delivery, molten material ejection, thermal gradient distribution, and plasma formation during the LBM process. The influence of these inputs is studied on four output responses: surface roughness (as an indicator of surface integrity), machining time (as a measure of productivity), surface hardness (to assess thermal effects on the microstructure), and burr thickness (to evaluate dimensional quality). Notably, the inclusion of burr thickness and hardness as output parameters adds a novel dimension to the study, since most past works have focused primarily on surface finish and material removal rate. Furthermore, the use of three distinct geometries enables the identification of laser–material interaction dynamics not only from a material perspective but also from a profile-dependent energy distribution and flow characteristic point of view.

The primary objective of this research is to optimize the LBM process parameters for XG3 steel across different geometrical configurations to achieve a balance between quality and productivity. Taguchi’s L_27_ orthogonal array is employed to design the experiments efficiently, ensuring that the interaction of all four variables is systematically captured for each hole shape^23^. Post-experimental analysis includes the application of Analysis of Variance (ANOVA) to determine the statistical significance and percentage contribution of each input on the respective output parameters. Genetic Algorithm (GA) is used for global optimization to derive the best combination of parameters that simultaneously minimize surface roughness, machining time, and burr thickness while maintaining acceptable surface hardness. To further enhance the utility of this work, predictive modelling has also been carried out using two different approaches: Response Surface Methodology (RSM) and Feed Forward Artificial Neural Network (BPANN)^24^. While RSM provides a mathematical model that fits second-order polynomial relationships between inputs and outputs, BPANN uses training-based machine learning to capture nonlinear interactions. These models are validated using statistical indicators and experimental verification, thereby offering a robust predictive framework for industrial application. The dual modelling approach contributes to both scientific understanding and practical implementation of LBM for high-strength alloys^25^.

This study addresses key gaps in current literature and makes several innovative contributions to advanced manufacturing. It pioneers a novel material process focus by presenting the first systematic investigation of Laser Beam Machining (LBM) on third-generation Advanced High-Strength Steel (AHSS), specifically 980 XG3, whose behavior under intense localized thermal cycles remains largely unexplored. Moving beyond conventional studies limited to simple geometries, it conducts a systematic evaluation of geometric complexity by comparing LBM performance across circular, triangular and square geometries under identical process parameters, revealing how toolpath dynamics and sharp features influence thermal distribution, melt flow and cut quality. A major contribution lies in its advanced predictive modeling and comparative analysis, where a high fidelity Back Propagation Artificial Neural Network (BPANN) is developed and validated to capture the highly non-linear interactions between process parameters and material response outperforming traditional Response Surface Methodology (RSM) with regression coefficients exceeding 0.999 and minimal Mean Absolute Percentage Error (MAPE). Finally, the study establishes an integrated optimization and prediction framework that unites Taguchi L_27_ Design of Experiments, ANOVA-based statistical analysis, Multi-Objective Genetic Algorithm (MOGA) optimization and BPANN modeling into a cohesive workflow. This holistic approach enables accurate process understanding, optimization, and implementation of LBM for complex, high-performance materials like XG3 steel.

## Methodology

**Fig. 2:**
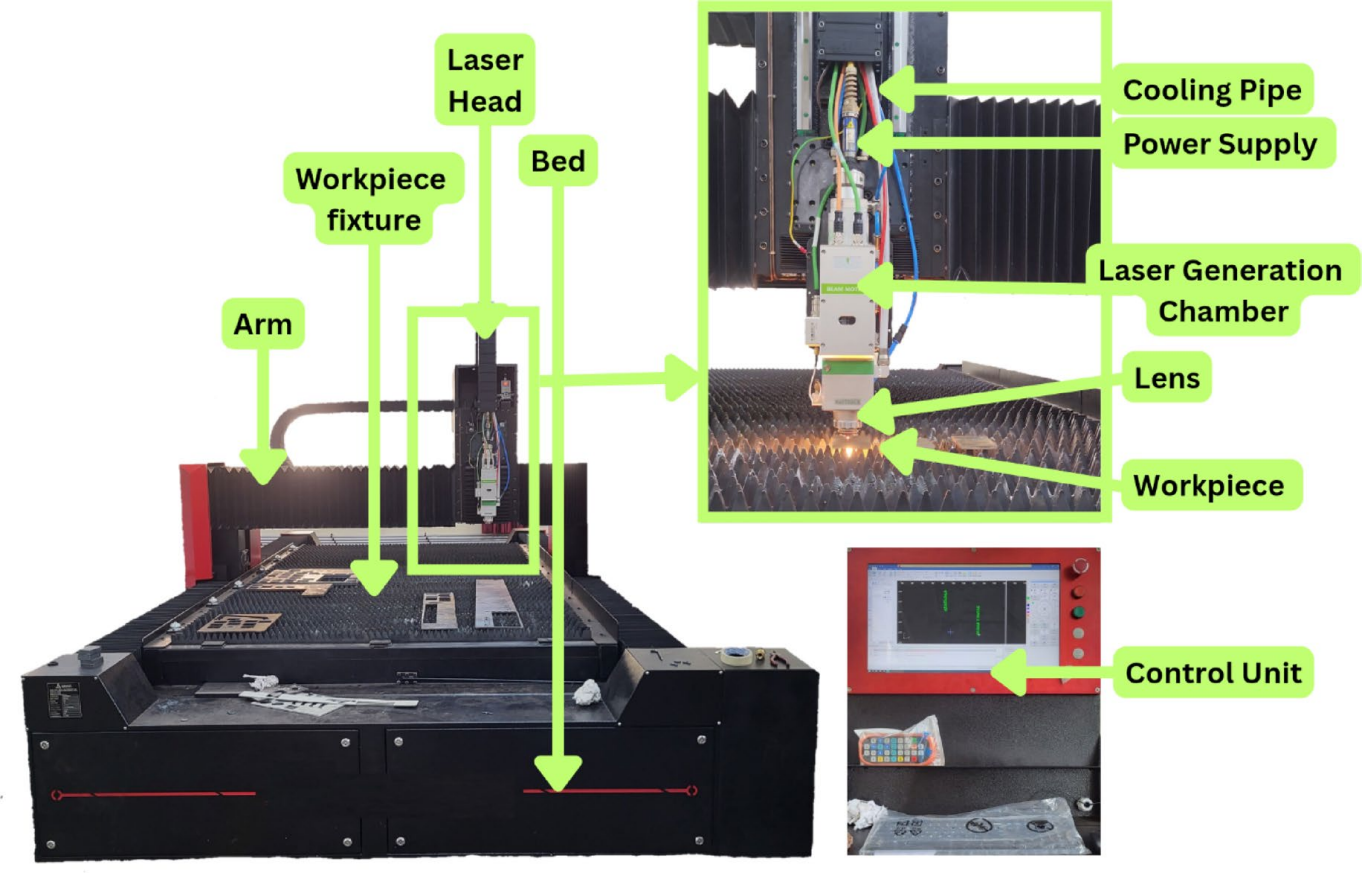
LASER beam machining system.

**Fig. 3:**
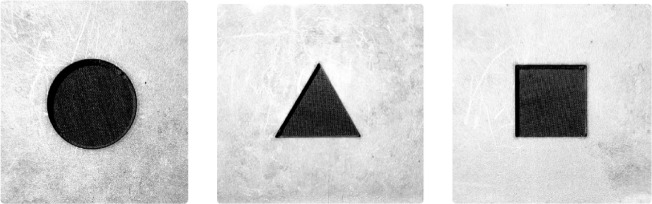
LASER beam machined holes on XG3 Steel workpiece.

XG3 steel plates of thickness 3 mm, 6 mm, and 9 mm were procured from AM Industries, Mumbai, for this research. XG3 is a high-performance, precipitation hardened stainless steel known for its superior combination of high strength, corrosion resistance, and thermal stability. It belongs to the category of maraging steels and is widely used in aerospace, defense, mould manufacturing and precision tooling due to its excellent dimensional accuracy and wear resistance. As per the data sheet provided by the manufacturer, the chemical composition of XG3 steel includes chromium, nickel, molybdenum, and cobalt, contributing to its excellent mechanical properties. The material possesses an ultimate tensile strength of 1350 MPa and a yield strength of 1200 MPa along with the hardness value of 275 to 280 Hv. These properties make XG3 steel difficult to machine by conventional processes. Therefore, laser beam machining (LBM), being a non-contact thermal energy-based process, is suitable for precision machining of such materials. The good dimensional stability and hardness retention of XG3 steel under thermal loads further support its suitability for LBM. The properties of XG3 steel used in this experimentation as provided by the vendor is presented in Table 1. The industrial relevance and challenging machinability of the material justify its selection for the present study on optimization of laser beam machining parameters. The use of 3 mm, 6 mm, and 9 mm thick plates enables detailed investigation into the influence of depth of cut and thermal input during the LBM process. Machining of XG3 steel plates was carried out using the SINAR LASER Beam Machining system (Fig. 2) equipped with a CO₂ laser source. The CO₂ laser operates in the infrared region with a wavelength of 10.6 μm and is known for its high power density, stable beam quality, and ability to machine reflective and hard materials such as stainless steels and maraging steels. The CO₂ laser used in this research is capable of delivering continuous wave output with controlled beam focusing, making it suitable for precise and repeatable cutting operations. The detailed specifications of the SINAR LBM system are provided in Table 2. In order to study the influence of laser beam machining parameters on different geometries, three hole shapes were selected for the experiment (circular, triangular, and square). The circular hole had a diameter of 10 mm, resulting in a perimeter of 31.42 mm. To maintain a constant cutting length and ensure uniform energy input across all shapes, the side lengths of the equilateral triangle and square were calculated such that their total perimeters also measured 31.42 mm. This standardization allowed for a fair comparison of machining performance irrespective of the shape complexity. After each laser cutting operation, a cooling period of 5 min was provided to the workpiece to allow it to return to room temperature. This step was essential to avoid thermal interference and heat accumulation, ensuring consistency in material response and accuracy across all cutting trials. The laser beam machined XG3 workpiece is depicted in Fig. 3.

**Table 1:** Nominal chemical composition of XG3 Steel.

Element	Content (wt%)
Carbon (C)	0.15–0.25
Manganese (Mn)	2.0–3.0
Silicon (Si)	1.0–2.0
Chromium (Cr)	< 0.5
Molybdenum (Mo)	< 0.3
Iron (Fe)	Balance

**Table 2:** Properties of LASER beam machining System.

Specification	Details
Machine Model	LCM-3015EC-O
Serial Number	MM03-023
LASER Type	Fiber LASER
LASER Power	3 kW
Wavelength	1064 nm
Supply Voltage	3 Phase AC, 415 V, 50 Hz
Control Voltage	24 V DC
Rated Current	63 A
Cooling Method	Water Cooling
Maximum Cutting Area	3000 mm × 1500 mm
Positioning Accuracy	± 0.02 mm
Repeatability	± 0.01 mm
Maximum Cutting Speed	40 m/min
Compressed Air Pressure	5–7 Bar
Assist Gas	Nitrogen/Oxygen
Control System	CNC Controller with LCD Display
Machine Dimensions	6000 mm × 3500 mm × 1800 mm
Machine Weight	4500 kg
Operating Environment	Temperature 15–35 °C, Humidity < 70%

The surface roughness of the inner walls of the holes machined using LASER Beam Machining (LBM) on XG3 steel was evaluated using a Talysurf Surtronic surface roughness measuring instrument. To ensure consistent and reliable results, the machined specimens were carefully cleaned using acetone to remove any loosely adhered debris or recast layers before measurement. The surface roughness values were measured for three different geometries of holes, namely circular, triangular, and square, with varying depths of 3 mm, 6 mm, and 9 mm. For each hole geometry and depth, at least three readings were taken at equidistant positions along the internal wall using a stylus-type contact probe fitted on the Surtronic instrument. The stylus tip, made of diamond with a nominal radius of 2 μm, was capable of accessing the internal surfaces and tracing the micro-topographical profile of the hole walls. To ensure accuracy, the measuring length (cut-off) was maintained at 0.8 mm with an evaluation length of 4.0 mm. The equipment was operated in the Ra mode, which corresponds to the arithmetic mean deviation of the roughness profile, as it is the most widely used parameter in machining studies. The stylus was inserted along the axis of the hole and positioned to contact the inner surface with a constant measuring force of 0.7 mN and then moved vertically while maintaining contact to capture the roughness variation over the hole depth. Care was taken to avoid surface damage due to excessive stylus pressure or misalignment. The average of the three measured values was reported as the representative surface roughness (Ra) for each hole type and depth combination. This procedure was repeated for all combinations of geometry and depth to study the influence of laser parameters and hole shape on the internal surface finish produced by LBM.

To ensure metrological traceability, the measurement uncertainty was evaluated according to the Guide to the Expression of Uncertainty in Measurement (GUM). The expanded uncertainty, U, was calculated as U = k × u_c_, where the coverage factor k = 2 (for a 95% confidence level) and u_c_ is the combined standard uncertainty. The combined uncertainty was determined by the root-sum-of-squares of Type A uncertainty (the standard deviation of repeated measurements) and Type B uncertainty (arising from instrument calibration, resolution, and environmental factors). Based on this analysis, the expanded uncertainty for the Ra measurements was determined to be ± 0.15 μm.

The machining time for each hole geometry and depth during LASER Beam Machining (LBM) of XG3 steel was directly obtained from the computer interface integrated with the LBM system. The machine’s software recorded the total duration taken from the moment the laser beam initiated interaction with the workpiece surface until the beam ceased after completing the machining of the hole. This duration included the time taken for beam focusing, material ablation, and completion of the desired profile based on the programmed toolpath. The recorded time for each trial was noted with a precision of ± 0.01 s, ensuring high accuracy and repeatability. These values were used to analyze the impact of laser parameters and geometry on the machining efficiency.

Further, to evaluate the surface hardness in the regions affected by the laser, Vickers hardness measurements were carried out using a standard Vickers Hardness Tester. The region considered for testing was a circular area with a 5 mm radial distance around the perimeter of the machined hole, which typically experienced thermal influence due to laser heating. The specimens were carefully ground and polished using emery papers followed by alumina paste to obtain a mirror-finish surface before hardness testing. A load of 500 gf was applied for a dwell time of 10 s using a diamond pyramidal indenter. For each specimen, five indentations were taken symmetrically around the hole within the defined 5 mm zone, and the average of these readings was reported as the representative surface hardness. The diagonal lengths of the impressions were measured using the inbuilt optical scale in the Vickers tester, with a resolution of ± 1 μm.

Burr thickness around the periphery of the machined holes was examined using a Trinocular Inverted Metallurgical Microscope (Model: Metzer MIM-301) equipped with an image acquisition and measurement software (MetRel Pro v6.2). The microscope was calibrated using a stage micrometer with a least count of 1 μm to ensure dimensional accuracy. After machining, the burrs formed at the exit edges of the holes were visually inspected under 100x magnification. Measurements were taken at four equally spaced positions along the periphery of each hole to capture any asymmetry in burr formation. The software allowed for digital line measurement directly on the captured images, and the average of the four values was considered the representative burr thickness for each hole type and depth. All specimens were mounted and leveled properly on the microscope stage to ensure that only the burr height extending from the edge surface was captured, eliminating any background noise or uneven surface contribution to reading.

For each of the 27 experimental conditions defined by the L27 array, all measurements (surface roughness, hardness, burr thickness) were performed three times at distinct, equidistant locations on the feature. The average value is reported in all results tables to minimize the impact of random error and local material inconsistencies. Machining time, being a deterministic output from the CNC controller for a given toolpath, was recorded as a single replicate per run.

### Design of experiments

Taguchi’s Design of Experiments (DOE) approach was selected for this study to systematically investigate the effect of multiple laser beam machining parameters on the quality of machined features with a reduced number of experimental trials. The L_27_ orthogonal array was chosen to accommodate four input parameters at three levels each, allowing for the analysis of main effects while maintaining experimental efficiency. A total of 81 experimental runs were conducted by machining 9 holes of each geometry (circular, triangular, and square) on XG3 steel plates of three different thicknesses (3 mm, 6 mm, and 9 mm). The selection of process parameters such as cutting speed, gas pressure, focus point, and depth of cut was based on the capabilities of the SINAR CO₂ laser system and relevant literature on laser machining of high-strength steels. The selected parameter ranges were within the operational limits of the machine and were identified to significantly influence LASER-material interaction mechanisms such as thermal diffusion, melt ejection, and kerf formation. Taguchi’s method was preferred due to its robustness in handling variability, its ability to identify optimal settings with minimal experimentation, and its widespread acceptance in machining research. The approach also enables signal-to-noise ratio analysis to identify parameter sensitivity and process stability. The levels and values of each parameter used in the study are listed in Table 3.

**Table 3:** TDOE factors and Levels.

Parameters	Levels		
	1	2	3
(A) Cutting Speed (m/min)	8	10	12
(B) Gas Pressure (Bar)	0.5	0.7	0.9
(C) Focus Point (mm)	2	4	6
(D) Depth of cut (mm)	3	6	9

### Optimization techniques

To optimize the LASER Beam Machining of XG3 steel, a combined approach involving Analysis of Variance (ANOVA), Multi-Objective Genetic Algorithm (MOGA), and Technique for Order Performance by Similarity to Ideal Solution (TOPSIS) was employed. ANOVA was used to identify the significant process parameters influencing surface roughness, hardness, burr thickness, and machining time. MOGA generated a set of optimal solutions by considering multiple objectives simultaneously. TOPSIS was then applied to rank these solutions based on their closeness to the ideal outcome, allowing the selection of the most effective machining condition.

#### Analysis of variance (ANOVA)

The experimental data obtained from the L_27_ orthogonal array were first entered in statistical software Minitab-15, where each column represents one control factor and the response columns hold the measured values of surface roughness, machining time, surface hardness and burr thickness. Analysis of variance was applied at a confidence level of 95% to separate the total variation into contributions from cutting speed, gas pressure, focus point and depth of cut. The software calculated the sum of squares, mean squares, F-ratio and p-value for every factor where, a p-value below 0.05 indicates that the factor has a statistically significant influence on the response. The percentage contribution P% for each factor was then obtained by dividing its sum of squares by the total sum of squares and multiplying by 100, which quantitatively depicts the percentage contribution of each parameter on the output. After identifying significant factors, main effects plots were generated for every response; which display the average response at each level of a factor while holding the other factors randomised, thus giving a clear visual indication of the trend. The optimum level for an individual response was chosen at the point where the mean value aligns with the desired objective i.e. minimum surface roughness and machining time, maximum surface hardness and minimum burr thickness.

#### Multi-Objective genetic algorithm (MOGA)

**Fig. 4:**
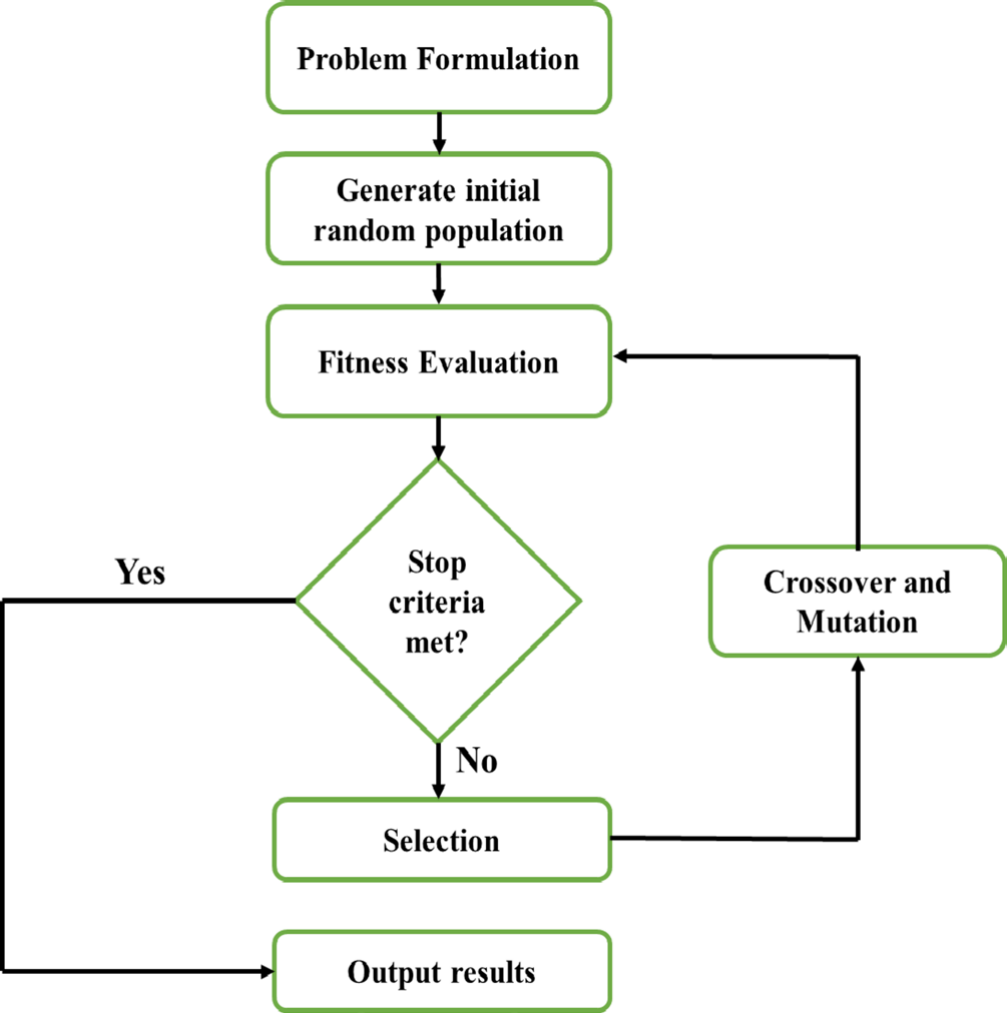
Flowchart indicating basic steps in Multi-objective GA.

Multi-objective genetic algorithms (MOGAs) are optimization algorithms that address the problem of finding a set of solutions to multiple conflicting objectives. MOGAs work on the principles of genetic algorithms (GAs) and extend the traditional GA framework to handle multiple objectives in a single optimization process. Finding a single solution that maximizes or minimizes a single objective function is the goal of conventional GAs. However, in many real-world optimization situations, several objectives may be equally important, and these objectives may be conflicting in nature. MOGAs are designed to handle these multi-objective optimization problems by generating a solution set, called the Pareto front, that represents a trade-off between the multiple objectives. MOGAs work by encoding the solutions as chromosomes in a population, which are evolved using the principles of natural selection and genetic variation. The selection process in MOGAs is based on the concept of Pareto dominance, where one solution is considered better than another if it is not dominated in any objective by the other solution. The MOGA aims to balance the trade-offs between the various objectives and sustain population diversity throughout the evolution process. The steps involved in a typical MOGA are shown in the flowchart in Fig. 4.

#### TOPSIS (Technique for order performance by similarity to ideal Solution)

The Pareto front was generated through MOGA optimization, and the TOPSIS method was employed to determine the best solution among the Pareto-optimal solutions. According to the specified criteria, TOPSIS, a multi-criteria decision-making (MCDM) technique, aids in choosing the best option from a list of alternatives. First proposed in 1981 by Hwang and Yoon, TOPSIS involves comparing alternatives to an ideal and worst solution to determine the best option. While the worst solution is the one that performs the poorest across all conditions, the ideal solution is the hypothetical alternative that meets all requirements with the best performance. When assessing alternatives, their similarity to the ideal solution and their dissimilarity to the worst solution is taken into account. TOPSIS is a popular method employed in different disciplines like engineering, economics, and environmental science. Nevertheless, the technique has certain limitations, such as the sensitivity of the results to the weights assigned to each criterion and the assumption that both the best and worst solutions have equal importance. The following are the fundamental steps involved in TOPSIS:

**Step 1**: Identify the criteria: Find the criteria which are relevant to the decision-making problem and create a matrix of alternatives by criteria values and assign weights.

**Step 2**: Normalize the matrix: Normalize the matrix of alternatives by criteria values so that each criterion has a comparable scale. This can be done by using the below Eq. 1.

1 $$\:{\stackrel{-}{X}}_{mn}=w\left(n\right)\cdot\:\left\{\frac{{X}_{mn}}{\sqrt{\sum\:_{j=1}^{n}{X}_{mn}^{2}}}\right\}$$

**Step 3**: Identify the ideal solutions: The best value for each criterion is represented by the positive ideal solution, while the negative ideal solution represents the worst value. These solutions can be obtained using the Eq. 2, Eq. 3, Eq. 4 and Eq. 5:

For maximization criteria:

2 $${\rm{Positive\:ideal\:solution(}}{{{V}}^{\rm{ + }}}{\rm{) = max(}}{{{\bar{X}}}_{\rm{mn}})}$$

3 $${\rm{Negative\:ideal\:solution(}}{{{V}}^{\rm{ - }}}{\rm{) = min(\bar{X}}}{{}_{\rm{mn}}}{)}$$

4 $${\rm{For\:minimization\:criteria:}}{{{V}}^{\rm{ + }}}{\rm{ = min(\bar{X}}}{_{\rm{mn}}}{)}$$

5 $${V^ - } = max(\bar{X}_{mn}),$$

where $\:{\stackrel{-}{X}}_{mn}$ is the normalized value for the m^th^ alternative and n^th^ criterion, and w(n) is the assigned weight.

**Step 4**: Calculate the distance: The distance of each alternative to the positive and negative ideal solutions can be calculated using a suitable distance measure, such as Euclidean distance, with the help of the following **Eq.** 6 and Eq. 7:

6 $$D_m^ + = \sqrt {\mathop \sum \limits_{n = 1}^k {{\left[ {{X_{mn}} - ~V_n^ + } \right]}^2}}$$

7 $$D_m^ - = \sqrt{{\sum_{n = 1}^{k}}[{X_{mn}} - V_n^ - ]{^2}}$$

where $\:{D}_{m}^{+}$ and $\:{D}_{m}^{-}$ are the distances of the m^th^ alternative to the positive and negative ideal solutions, respectively; $\:{X}_{mn}$is the normalized value of the m^th^ alternative for the n^th^ criterion.

**Step 5**: Estimate the closeness: The closeness of each alternative to both the best and worst values can be computed utilizing the subsequent Eq. 8 as given below:

8 $$C_{m}=\frac{{D_m^ - }}{{\left( {D_m^ - ~ + ~D_m^ + } \right)~}}$$

where $\:{C}_{m}$ is the closeness index of the m^th^ alternative to the ideal solution.

**Step 6**: Ranking the alternatives: Arrange the alternatives in order of their proximity to the ideal solution, with the alternative having the highest closeness index considered the most favorable option.

The Pareto front generated by MOGA contains a set of equally optimal solutions. To select a single, best-compromise solution from this set, a multi-criteria decision-making (MCDM) technique is required. For this study, the Technique for Order Performance by Similarity to Ideal Solution (TOPSIS) was chosen. The selection of TOPSIS is justified by several key advantages that make it particularly suitable for this type of engineering optimization problem. The core principle of TOPSIS is simple and intuitively logical as it identifies the best alternative as the one having the shortest Euclidean distance from the Positive Ideal Solution (PIS) and the farthest distance from the Negative Ideal Solution (NIS). It is also computationally efficient, enabling quick ranking of a finite set of alternatives, which makes it especially practical for post processing solutions on a Pareto front. Moreover, its compensatory nature allows trade offs between criteria, where a weak performance in one objective can be balanced by strong performance in another, closely reflecting real world decision making in manufacturing. Finally, TOPSIS is a well established and widely validated MCDM method, successfully applied in numerous manufacturing and engineering optimization scenarios.

### Prediction techniques

For predictive modeling of the LASER Beam Machining process on XG3 steel, two approaches were used: Response Surface Methodology (RSM) and Backpropagation Artificial Neural Network (BPANN). RSM was employed to develop regression-based mathematical models that describe the relationship between input parameters and output responses. In parallel, BPANN was used to capture complex nonlinear interactions among variables and predict machining performance with higher accuracy. Together, these methods provided a reliable framework for predicting outcomes under various process conditions.

#### Response surface methodology (RSM)

Response Surface Methodology (RSM) was used in this study to develop mathematical models for predicting the output responses such as surface roughness, machining time, surface hardness, and burr formation during laser beam machining of XG3 steel. RSM is a statistical and mathematical technique that fits a second-order polynomial equation to experimental data in order to describe the relationship between the input variables and output responses. The method is particularly useful for modelling and optimizing processes where multiple variables influence the response in a non-linear manner. Based on the results from the Taguchi L_27_ orthogonal array, the data were imported into statistical software such as Design Expert or Minitab, and coded variables were used for better numerical stability during model development.

The general second-order regression equation used in RSM is provided in **Eq. **9**.**

9 $${\rm Y=\upbeta_{0}+\sum\upbeta_{i}X_{i}+\sum\upbeta_{ii}X_{i}^{2}+\sum\upbeta_{ij}X_{i}X_{j}+\varepsilon}$$

Where, Y is the predicted response, β₀ is the intercept, β_i_ are the linear coefficients, β_i__i_ are the quadratic coefficients, β_i_ⱼ are the interaction coefficients, X_i_ and Xⱼ are the input parameters (cutting speed, gas pressure, focus point, and depth of cut), and ε is the residual error.

The coefficients were estimated using least square regression analysis. The adequacy of the model was tested using ANOVA, and the model with high R² value, adjusted R² and significant F-value was selected. Terms with p-values less than 0.05 were considered statistically significant. The developed model was used to predict the responses within the range of selected parameter levels.

To better understand the interactions between two parameters and their combined effect on the response, contour plots were generated. These plots are two-dimensional graphical representations of the response surface where each contour line represents a constant value of the response variable. By fixing two variables and varying the remaining ones within their range, the plots help to identify regions of optimal performance and the sensitivity of the response to changes in parameters. The shape of the contour also indicates the degree of interaction where, circular contours suggest weak interaction, while elliptical contours indicate strong interaction between the variables. These plots provide an intuitive and visual method for identifying the combination of parameters that result in the best machining performance.

#### Back-Propagation artificial neural network (BPANN)

**Fig. 5:**
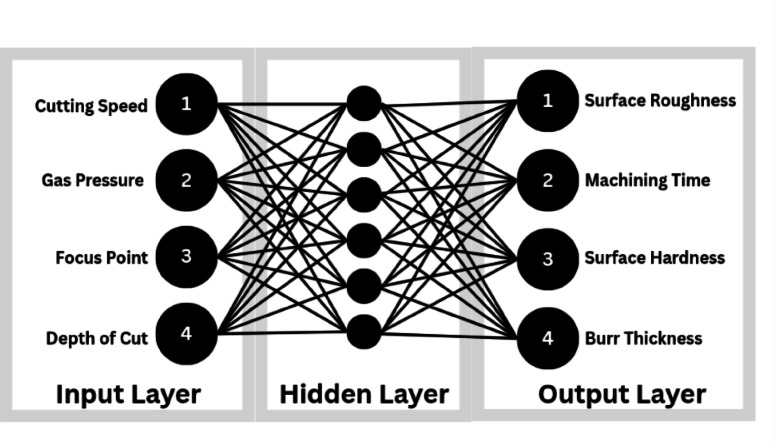
Representation of BPANN algorithm.

In the present study, a Back Propagation Artificial Neural Network (BPANN) (Fig. 5) was developed to predict the output responses such as surface roughness, machining time, surface hardness, and burr formation during the laser beam machining of XG3 steel. BPANN is a supervised machine learning algorithm based on the structure and functioning of biological neural networks. It is capable of modelling complex non-linear relationships between input parameters and output responses, especially in cases where traditional statistical methods may not yield high accuracy.

The BPANN model (Table 4) was developed using MATLAB Neural Network Toolbox. The experimental data obtained from the L_27_ Taguchi orthogonal array served as the dataset for training and testing the network. The input layer of the network consisted of four neurons corresponding to the four input parameters (cutting speed, gas pressure, focal point, and depth of cut). The output layer had four neurons representing response parameters (surface roughness, machining time, surface hardness and burr thickness) and separate networks were trained for each response variable. The data were first normalised in the range of 0 to 1 to improve training efficiency and avoid dominance of higher numerical values.

A trial-and-error approach was used to select the optimal number of neurons in the hidden layer. Various network configurations were tested by varying the number of hidden neurons from 5 to 20. The transfer function used between the input and hidden layer was ‘tansig’ (hyperbolic tangent sigmoid), and the transfer function from the hidden layer to the output layer was ‘purelin’ (linear function). The training algorithm used was Levenberg-Marquardt backpropagation (trainlm), which is known for fast convergence and high accuracy in function approximation problems.

The dataset was divided into three parts i.e. 70% for training, 15% for validation, and 15% for testing. The training phase involved adjusting the weights and biases of the network using the backpropagation algorithm, which minimises the mean square error between the predicted and actual outputs by updating weights in the reverse direction of the error gradient. During training, the performance was monitored through the validation data to prevent overfitting. Once the network was trained successfully, its performance was evaluated using metrics such as mean absolute error (MAE), root mean square error (RMSE), and coefficient of determination (R²).

After achieving acceptable accuracy, the trained BPANN model was used to predict output responses for any combination of input parameters within the experimental range. The model was also used to generate response surfaces and compare its prediction accuracy with those obtained from response surface methodology. The BPANN proved to be a reliable and accurate tool for predicting non-linear machining behaviour, and its flexibility makes it suitable for future optimisation tasks involving multi-objective functions or larger datasets.

The choice of a BPANN model is justified by its inherent strength as a universal function approximator, making it exceptionally well suited for modeling complex, non linear physical processes like Laser Beam Machining (LBM). The interaction between a high energy laser beam and a multi phase alloy such as XG3 involves rapid thermal cycles that can trigger localized phase transformations, altering microstructure and properties in ways that simple mathematical models cannot easily capture. The BPANN’s multi-layer perceptron architecture, equipped with non linear activation functions, enables it to learn these intricate underlying relationships directly from experimental data without assuming any predefined functional form. Although other machine learning algorithms can also perform regression tasks, BPANN serves as a powerful and well established benchmark. Compared to Support Vector Machines (SVM), which are effective for regression but less adept at modeling highly continuous and complex relationships, a well tuned neural network can often deliver superior predictive accuracy through deeper feature learning. Similarly, while Random Forests (RF) offer robustness against overfitting, BPANNs excel at capturing subtle, continuous non linearities typical of thermal processes. Considering these strengths, the BPANN was selected as a strong candidate for achieving high predictive fidelity in this application.

**Table 4:** BPANN model architecture and training parameters.

Parameter	Specification
Network Type	Feed-Forward Back-Propagation
Input Neurons	4 (Cutting Speed, Gas Pressure, Focus Point, Depth of Cut)
Hidden Layers	1
Hidden Neurons	18
Output Neurons	4 (Ra, Time, Hardness, Burr Thickness) - Separate networks trained for each response
Hidden Layer Activation Function	Hyperbolic Tangent Sigmoid (‘tansig’)
Output Layer Activation Function	Linear (‘purelin’)
Training Algorithm	Levenberg-Marquardt (‘trainlm’)
Data Division	70% Training, 15% Validation, 15% Testing

## Results and discussion

This section presents a detailed analysis of how the selected input parameters, i.e. cutting speed, gas pressure, focus point, and depth of cut affect the machining performance of XG3 steel during LASER Beam Machining for three different hole geometries: circular, triangular and rectangular. The response variables studied include surface roughness, machining time, surface hardness and burr thickness, which are critical indicators of dimensional accuracy, thermal impact and surface integrity. The interaction between laser parameters and shape geometry is explored to understand how each factor influences the laser-material interaction and the quality of machined features.

### Optimization of response parameters using analysis of variance (ANOVA)

Optimization of response parameters was carried out using Analysis of Variance (ANOVA), a statistical tool employed to determine the significance and contribution of each input factor on the selected output responses. By evaluating the F-values and associated p-values, ANOVA helps identify the most influential parameters and their interactions, thereby guiding the selection of optimal conditions for improved performance. This method ensures that the observed variations in the output are statistically validated and not due to random experimental error.

#### Surface roughness

The experimental results (Table 5) show clear trends in surface roughness variation with changes in cutting speed, gas pressure, focus point, and depth of cut for the three hole geometries (circle, triangle, and square). The material under study, XG3 steel, is a high-strength, precipitation-hardened maraging steel known for its hardness and thermal stability, which influences the laser-material interaction during machining.

During the analysis of the effect of cutting speed, it can be deduced that, as the speed increases from 8 to 12 m/min, a general reduction in surface roughness is observed across all geometries. For example, circular holes have surface roughness decreasing from above 11 μm at 8 m/min to around 10.1 μm at 12 m/min (trial 21). This can be explained by the fact that at higher cutting speeds, the laser moves faster over the material surface, reducing the heat input per unit length. This limits the heat-affected zone and minimizes excessive melting and resolidification irregularities, producing smoother surfaces. The rapid movement also decreases the interaction time between the laser beam and the material, resulting in finer kerfs and better surface integrity.

Further, gas pressure also shows a significant influence on the machining output. At low pressure (0.5 bar), surface roughness is generally higher compared to values at 0.7 and 0.9 bar. Higher gas pressure assists in efficient removal of molten material and vaporised particles from the kerf. This prevents re-solidification of molten droplets on the machined surface, which otherwise increases surface roughness. For instance, at cutting speed 12 m/min and focus point 6 mm, the circle surface roughness improves from 10.6 μm (0.5 bar) to 10.24 μm (0.9 bar) (trials 20 and 25). The assist gas also acts as a coolant, reducing thermal gradients and micro-cracking, further improving surface finish.

Furthermore, focus point location controls the laser beam spot size and intensity on the workpiece surface. When the focus point moves away from the surface (for example, from 2 mm to 6 mm), the beam intensity distribution changes, affecting melting and vaporisation characteristics. In the results, intermediate focus positions such as 4–6 mm show relatively lower surface roughness compared to 2 mm in many cases, indicating better energy distribution and controlled thermal input. This results in stable melt pools and uniform material removal. For example, at 12 m/min and gas pressure 0.5 bar, surface roughness for the circle hole is lowest at 6 mm focus (trial 21, 10.1 μm) compared to 2 mm (trial 22, 10.43 μm).

Finally, depth of cut influences heat accumulation and material removal volume per pass. Increasing the depth from 3 mm to 9 mm leads to a slight increase in surface roughness due to increased thermal loading, which can cause uneven melting, recast layers, and micro-cracking on the cut surface. The molten pool becomes larger, and heat dissipation becomes less efficient, especially for thicker plates, causing more irregularities. For example, for trial 1 (3 mm depth) circular roughness is 11.2 μm, increasing to 12.1 μm at 9 mm depth (trial 3).

Comparing the geometries, circular holes consistently show lower surface roughness values than triangular and square holes at the same parameter settings. This difference can be attributed to the effect of shape complexity on heat distribution and melt flow dynamics. The curved geometry of the circle allows more uniform energy distribution and smoother melt ejection compared to sharp corners of triangle and square shapes, which tend to concentrate thermal stresses and cause more irregular solidification patterns, increasing surface roughness. For example, trial 1 shows circular roughness 11.2 μm, triangular 16.87 μm, and square 18.95 μm, demonstrating this effect clearly.

**Table 5:** Experimental results of surface roughness (µm).

Trial Number	Cutting Speed (m/min)	Gas Pressure (Bar)	Focus Point (mm)	Depth of Cut (mm)	Circle Surface Roughness (µm)	Triangle Surface Roughness (µm)	Square Surface Roughness (µm)
1	8	0.5	2	3	11.2	16.87	18.95
2	8	0.5	2	6	11.6	17.37	19.52
3	8	0.5	2	9	12.1	18.2	20.42
4	8	0.7	4	3	11.42	17.26	19.45
5	8	0.7	4	6	11.67	17.63	19.9
6	8	0.7	4	9	11.93	17.95	20.13
7	8	0.9	6	3	11.64	17.39	19.56
8	8	0.9	6	6	11.89	17.84	20.04
9	8	0.9	6	9	12.15	18.21	20.46
10	10	0.5	4	3	11.3	15.56	19.01
11	10	0.5	4	6	10.6	16.41	17.46
12	10	0.5	4	9	10.9	16.89	18.45
13	10	0.7	6	3	10.79	15.78	18.2
14	10	0.7	6	6	10.55	16.09	17.78
15	10	0.7	6	9	10.79	16.15	18.12
16	10	0.9	2	3	10.76	15.7	18.23
17	10	0.9	2	6	10.52	16.1	17.65
18	10	0.9	2	9	10.76	16.12	18.1
19	12	0.5	6	3	10.4	15.2	17.56
20	12	0.5	6	6	10.6	15.65	17.83
21	12	0.5	6	9	10.1	15.93	17.01
22	12	0.7	2	3	10.43	15.68	17.6
23	12	0.7	2	6	10.63	15.98	17.9
24	12	0.7	2	9	10.83	16.28	18.25
25	12	0.9	4	3	10.24	15.43	17.35
26	12	0.9	4	6	10.44	15.78	17.84
27	12	0.9	4	9	10.64	16.02	18.02

**Fig. 6:**
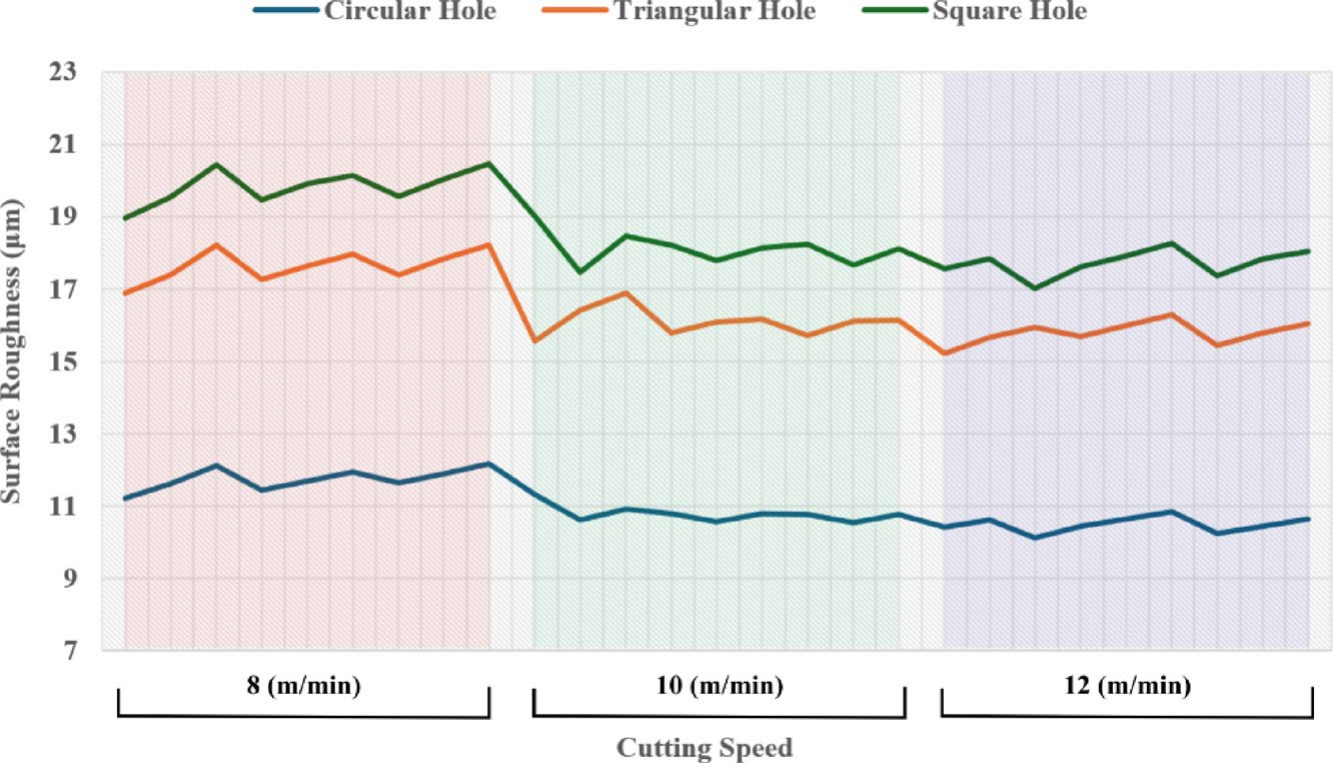
Effect of cutting speed on surface roughness across different hole geometries.

From Fig. 6 it can be deduced that, surface roughness exhibits a clear negative correlation with increasing cutting speed across all geometries, with circular holes achieving the finest finishes (10.1–12.1 μm), followed by triangular (15.2–18.2 μm) and square (17.01–20.46 μm) profiles. The optimization landscape illustrated by the plots reveals that the minimum surface roughness for circular holes (approximately 10.1 μm) occurs at the maximum cutting speed of 12 m/min combined with intermediate gas pressures (0.5–0.7 bar) and optimal focus positioning (6 mm). The geometrically-induced variations in surface quality reflect differential melt pool dynamics; circular profiles facilitate continuous laser advancement without directional changes, enabling uniform melt ejection and minimal recast layer accumulation. Conversely, polygonal geometries require repeated beam deceleration and repositioning at corners, inducing localized thermal accumulation and irregular solidification patterns that elevate roughness by 5–8 μm relative to circular cuts. The plots consistently demonstrate that the parameter window yielding optimal surface finish (cutting speed: 12 m/min, gas pressure: 0.5–0.9 bar, focus point: 6 mm, depth: 3 mm) corresponds to conditions minimizing heat-affected zone extent and thermal degradation, thereby confirming that rapid thermal cycling at elevated cutting speeds represents the most effective strategy for achieving superior surface integrity in laser-machined precision components.

**Fig. 7:**
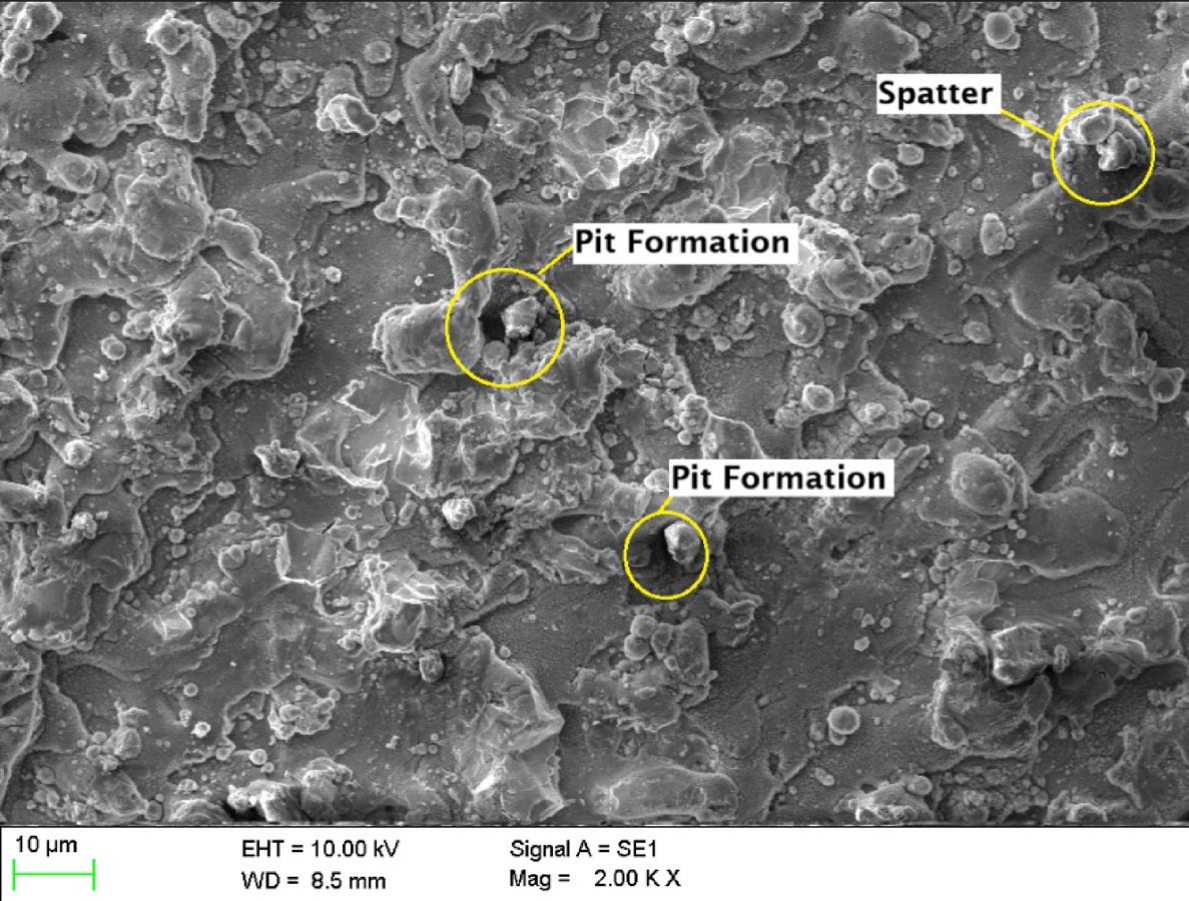
Scanning Electron Microscopic image of laser beam machined circular hole at a cutting speed of 8 m/min.

The morphology of machined surfaces is significantly influenced by cutting speed, as evidenced by scanning electron microscopy (SEM) analysis. At a cutting speed of 8 m/min **(**Fig. 7**)**, the surface exhibits a rugged and irregular topography characterized by extensive re-solidified molten material, numerous spherical micro-particles, and pronounced irregular recast layers. This indicates substantial thermal damage, manifested as crater-like depressions and microcracks, attributable to prolonged interaction time and excessive heat input inherent to lower cutting speeds.

**Fig. 8:**
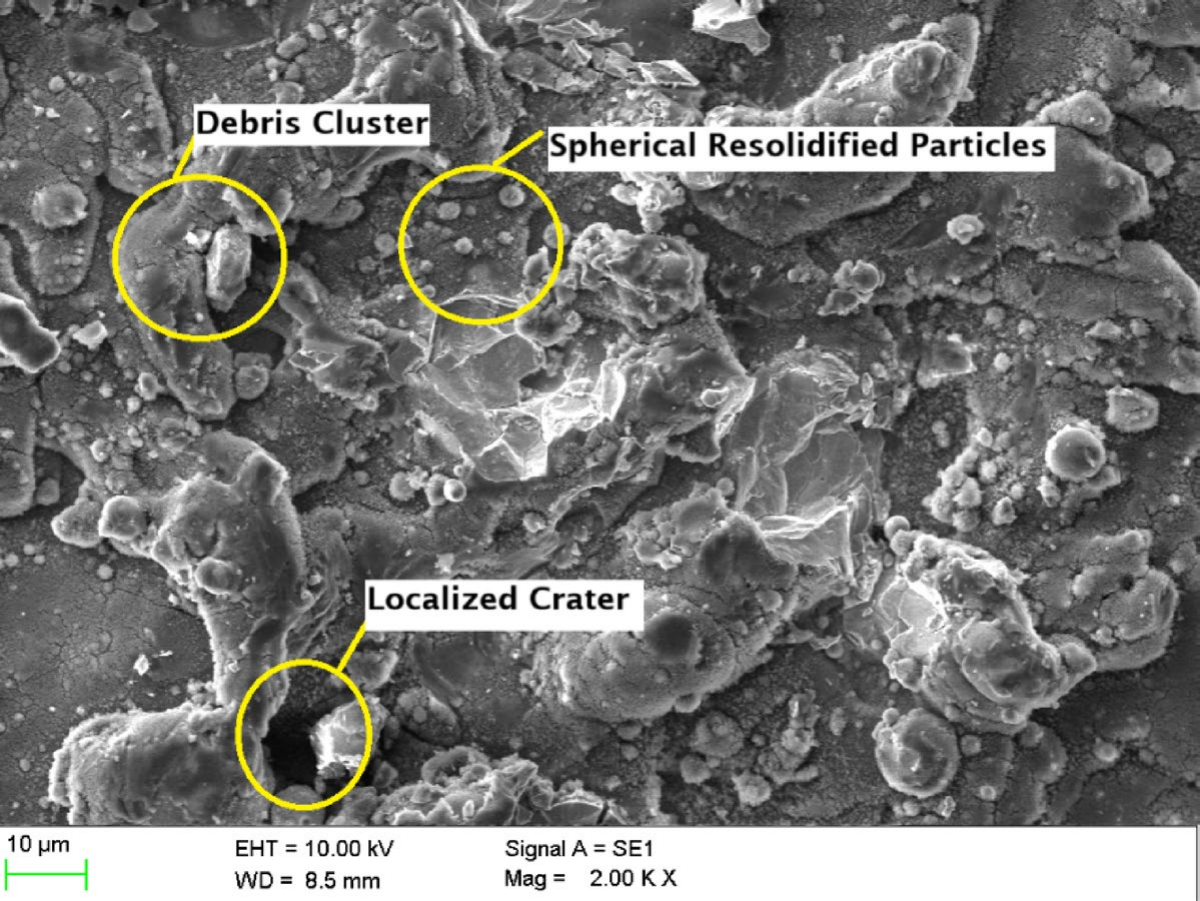
Scanning Electron Microscopic image of laser beam machined circular hole at a cutting speed of 10 m/min.

Conversely, an increase in cutting speed to 10 m/min **(**Fig. 8**)** yields a comparatively improved surface with moderate roughness. While microcracks and debris clusters persist, the overall morphology suggests more efficient material removal and diminished thermal effects. This is further corroborated by a reduction in molten pool size and fewer re-solidified globules, indicative of a more balanced energy distribution.

**Fig. 9:**
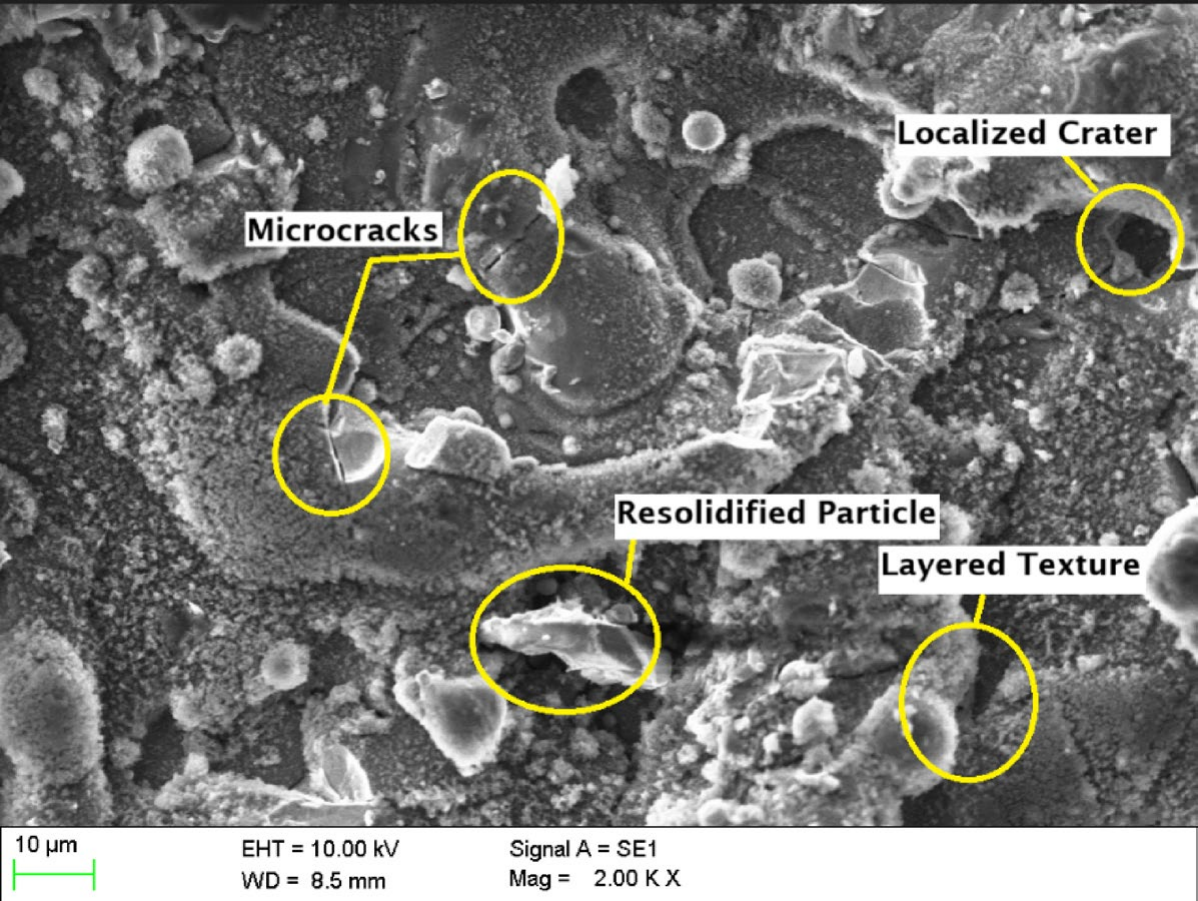
Scanning electron microscopic image of laser beam machined circular hole at a cutting speed of 12 m/min.

Optimizing the cutting process further, 12 m/min **(**Fig. 9**)** results in a notably smoother surface, characterized by a thinner recast layer and reduced microcrack density. The higher cutting speed effectively mitigates thermal load, thereby limiting the heat-affected zone and promoting minimal spatter, finer texture, and enhanced surface uniformity. However, the occasional presence of fine cracks and shallow pits at this higher speed suggests potential insufficient melting, highlighting a complex interplay between cutting parameters and material response.

The analysis of variance for surface roughness (Table 6) reveals that cutting speed is the most influential factor across all three hole geometries. It contributes 82.67% for circular holes, 82.376% for triangular holes, and 82.409% for square holes, with highly significant p-values of 0.000 in all cases. Gas pressure, focus point, and depth of cut show negligible effects on surface roughness, with percentage contributions below 1.1% and p-values well above the significance threshold, indicating statistical insignificance. Among the interaction effects, the interaction between cutting speed and depth of cut shows a noticeable influence, particularly for triangular holes with a contribution of 12.51% and a p-value of 0.026, indicating statistical significance. This interaction is also moderately significant for square holes with a contribution of 9.334% and a p-value of 0.076. All other interactions have minimal impact on surface roughness. The residual standard error is lowest for triangular holes at 3.046, followed by square holes at 3.811 and circular holes at 5.50, indicating that the model provides a better fit for triangular geometries.

**Table 6:** Analysis of variance for surface roughness.

Source	DF	Circular Hole			Triangular Hole			Square Hole		
		F	*P*	*P*%	F	*P*	*P*%	F	*P*	*P*%
A	2	45.05	0.000	82.67	81.13	0.000	82.376	64.87	0.000	82.409
B	2	0.03	0.972	0.052	0.16	0.855	0.162	0.27	0.770	0.346
C	2	0.04	0.963	0.069	0.16	0.853	0.164	0.24	0.795	0.302
D	2	0.45	0.660	0.819	0.33	0.732	0.333	0.80	0.494	1.0114
A*D	4	2.36	0.166	8.669	6.16	0.026	12.51	3.67	0.076	9.3340
B*D	4	0.09	0.983	0.32	0.24	0.908	0.481	0.10	0.977	0.2643
C*D	4	0.51	0.730	1.88	0.45	0.768	0.919	0.99	0.479	2.5203
RSE	6			5.50			3.046			3.811
Total	26									

Where, A – Cutting Speed, B – Gas Pressure, C – Focus Point, D – Depth of Cut, RSE – Residual Error.

**Fig. 10:**
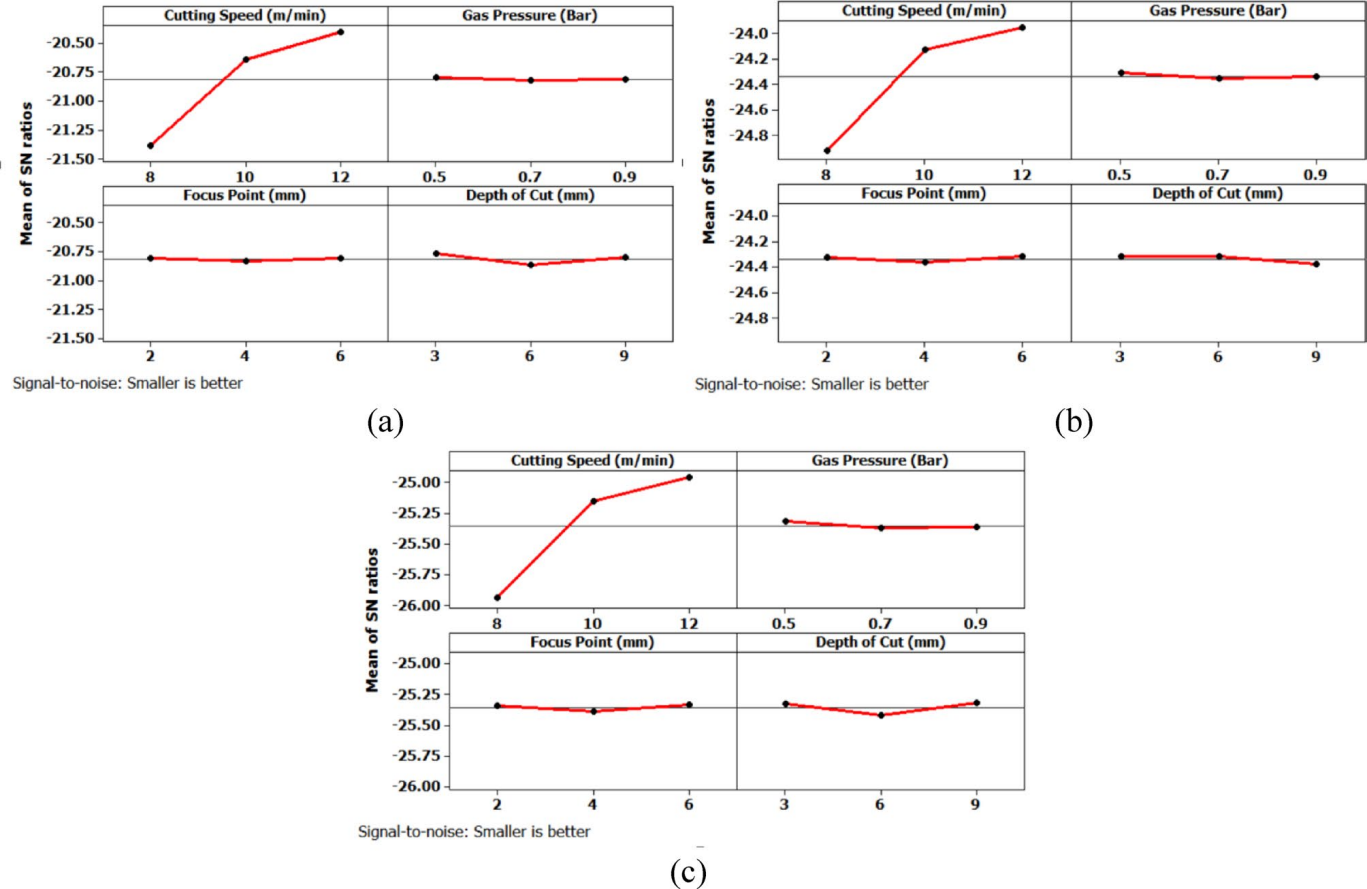
Main Effects plot for surface roughness; (a) Circular Hole; (b) Triangular Hole; (c) Square Hole.

Further analysis of the main effects plot for the Signal-to-Noise ratio (Fig. 10) confirms that the optimal combination of process parameters for achieving the lowest surface roughness across all three hole geometries corresponds to a cutting speed of 12 m/min, gas pressure of 0.5 bar, focus point of 2 mm, and depth of cut of 3 mm. Among the levels considered in the experimental design, 12 m/min represents the highest cutting speed, while 0.5 bar is the lowest gas pressure level. Similarly, 2 mm is the minimum focus point and 3 mm is the shallowest depth of cut employed. This combination appears to favorably influence the machining process by enabling cleaner material removal and reduced thermal degradation at the cut interface, thereby minimizing surface irregularities. The results indicate that higher cutting speed promotes efficient cutting with minimal tool-material interaction time, while lower gas pressure and reduced focus point contribute to a more concentrated energy delivery with minimal turbulence. The smallest depth of cut likely reduces mechanical and thermal stresses at the cutting interface, contributing to an improved surface finish.

#### Machining time

The machining time data (Table 7) reveal the dynamic interaction between process parameters and material behavior during laser cutting of XG3 steel. This maraging steel exhibits high thermal conductivity, mechanical strength, and a uniform microstructure due to its controlled composition. These characteristics influence how efficiently energy from the CO₂ laser beam is absorbed and converted into material removal. Across all geometries, machining time decreases as cutting speed increases, validating the inverse relation between feed rate and exposure duration of the laser beam.

At the lowest cutting speed (8 m/min), the machining time is the highest for all geometries, indicating a slower progression of the laser beam and hence longer exposure at each point. For instance, in trial 3, circular machining time is 2.5 s, triangle is 4.32 s, and square is 5.1 s. As cutting speed increases to 12 m/min (trial 21), the machining time drops significantly to 1.1 s for the circle, 2.76 for triangle, and 2.32 for square. This trend is scientifically supported by the principle that higher cutting speeds allow the laser beam to traverse the material faster, reducing heat accumulation and melt pool size, resulting in faster cuts. However, this must be optimized because excessively high speeds may reduce penetration or cutting depth in tougher steels like XG3. Further, gas pressure plays a dual role in assisting material removal and cooling the kerf zone. Increasing gas pressure improves the ejection of molten material from the kerf, which in turn speeds up the cutting process. For example, in trial 5 (0.7 bar), triangle machining time is 4.01 s, whereas in trial 8 (0.9 bar) under same speed and depth, the time improves to 4.1 s, showing slight benefit from enhanced melt expulsion. This effect is more prominent at higher cutting speeds, where molten ejection and cooling need to be more efficient to maintain continuous cutting. Furthermore, focus point determines the energy density at the surface. A properly focused beam ensures optimal thermal energy delivery into the steel. At 6 mm focus distance, where energy distribution appears to match the material absorption and thermal diffusion characteristics well, machining times are lower. In trial 19, with focus at 6 mm, the machining time for circle is just 1.4 s, which is lower compared to trial 22 (focus 2 mm), where it increases to 1.3 s despite same cutting speed. This implies that improper focus may lead to defocused energy distribution, reducing cutting efficiency due to lower beam intensity at the focal point.

Finally, depth of cut has a directly proportional relationship with machining time. As depth increases, the laser must penetrate deeper layers, increasing energy requirement and beam dwell time. In trial 1 (3 mm depth), the triangle takes 3.3 s, which increases to 4.32 s in trial 3 (9 mm depth) under same conditions. This is due to increased thermal resistance and material volume requiring more sustained energy for complete melting and ejection. For deeper cuts in maraging steel, controlled thermal input and efficient gas-assisted expulsion are critical to maintaining cutting rates.

Geometrically, circular holes consistently exhibit the shortest machining time, followed by triangular and square holes. This is attributed to the uniformity of motion during laser tracing. Circles provide continuous curvature with no sharp corners, allowing uninterrupted beam movement. In contrast, triangle and square shapes involve sudden directional changes and sharp angles, requiring deceleration and repositioning of the laser head. These pauses increase machining time and induce localized thermal accumulation. For example, in trial 16, machining time for circle is 1.59 s, triangle is 2.4, and square is 3.2, clearly demonstrating the effect of geometric complexity on time consumption.

**Table 7:** Experimental results of machining time (s).

Trial Number	Cutting Speed (m/min)	Gas Pressure (Bar)	Focus Point (mm)	Depth of Cut (mm)	Circle Machining Time (s)	Triangle Machining Time (s)	Square Machining Time (s)
1	8	0.5	2	3	1.9	3.3	3.8
2	8	0.5	2	6	2.2	3.86	4.5
3	8	0.5	2	9	2.5	4.32	5.1
4	8	0.7	4	3	2.07	3.66	4.22
5	8	0.7	4	6	2.32	4.01	4.72
6	8	0.7	4	9	2.58	4.5	5.27
7	8	0.9	6	3	2.17	3.6	4.23
8	8	0.9	6	6	2.43	4.1	4.85
9	8	0.9	6	9	2.68	4.5	5.25
10	10	0.5	4	3	2.1	2.52	4.16
11	10	0.5	4	6	1.5	3.12	2.99
12	10	0.5	4	9	1.8	3.54	3.65
13	10	0.7	6	3	1.64	2.49	3.34
14	10	0.7	6	6	1.45	2.82	2.92
15	10	0.7	6	9	1.64	2.85	3.31
16	10	0.9	2	3	1.59	2.4	3.2
17	10	0.9	2	6	1.41	2.72	2.81
18	10	0.9	2	9	1.59	2.73	3.18
19	12	0.5	6	3	1.4	1.97	2.84
20	12	0.5	6	6	1.6	2.41	3.25
21	12	0.5	6	9	1.1	2.76	2.32
22	12	0.7	2	3	1.3	2.21	2.61
23	12	0.7	2	6	1.5	2.56	3.01
24	12	0.7	2	9	1.7	2.9	3.4
25	12	0.9	4	3	1.26	2.12	2.5
26	12	0.9	4	6	1.46	2.47	2.9
27	12	0.9	4	9	1.66	2.81	3.3

The analysis of variance for machining time (Table 8) reveals that cutting speed is the most dominant factor influencing the response across all three hole geometries. It contributes 74.385% for circular holes, 76.19% for triangular holes, and 75.43% for square holes, with statistically significant p-values of 0.001, 0.000, and 0.000 respectively. In contrast, gas pressure, focus point, and depth of cut show minimal influence on machining time, with percentage contributions below 1.7% and p-values far above the accepted significance threshold, indicating their statistical insignificance. Among the interaction terms, the interaction between cutting speed and depth of cut exhibits a considerable effect, especially in the case of triangular holes where it contributes 18.99% with a significant p-value of 0.006. For circular and square holes, this interaction shows moderate influence with contributions of 10.62% and 11.34% respectively, though the statistical significance is marginal. All other interaction effects, including B*D and C*D, are found to be insignificant across all geometries. The residual standard error is lowest for triangular holes at 2.539, indicating a good model fit, followed by square holes at 6.105. These observations clearly establish cutting speed as the critical parameter governing machining time, with its interaction with depth of cut having a secondary but relevant impact, particularly in triangular hole geometry.

**Fig. 11:**
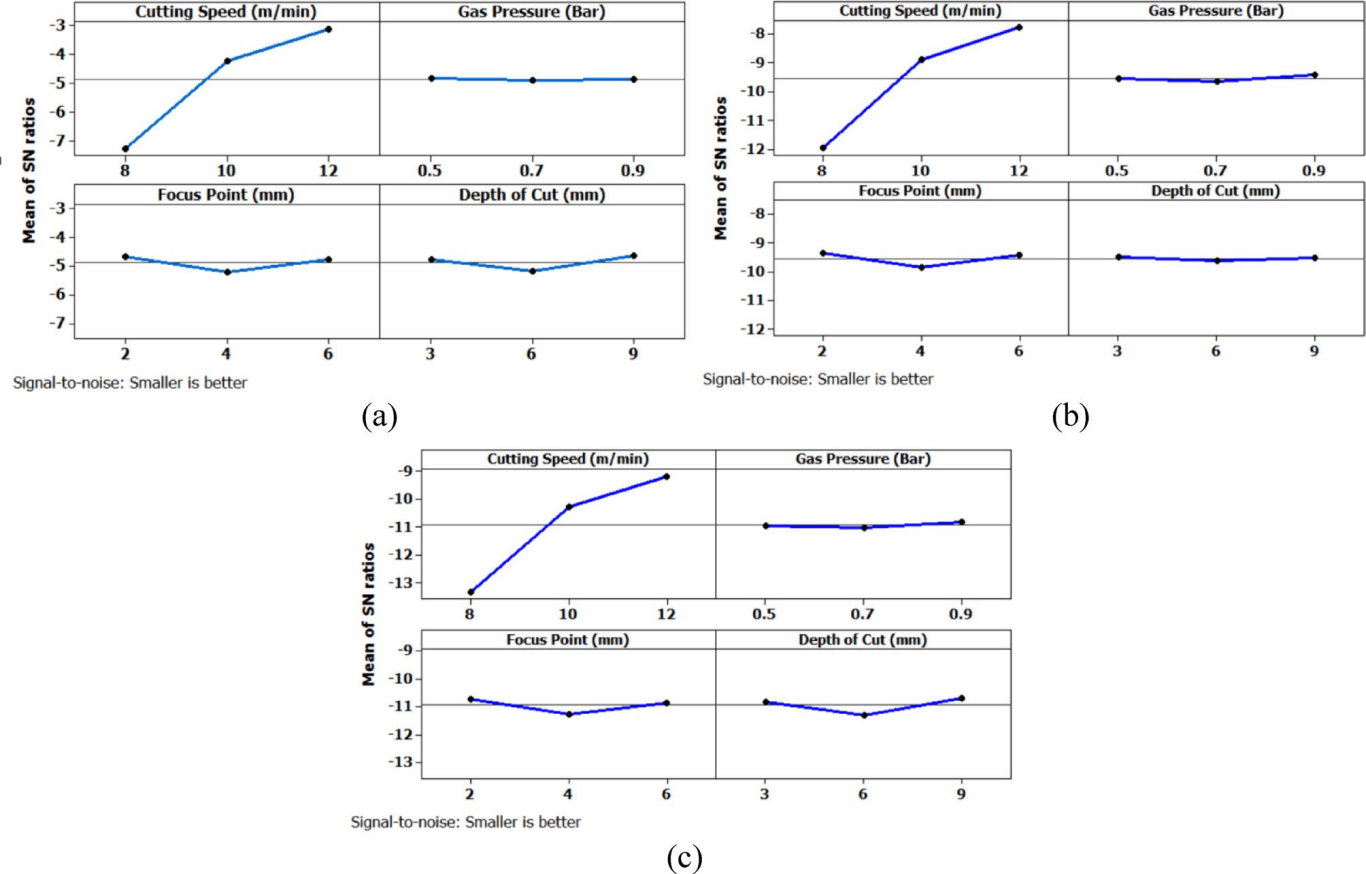
Main Effects plot for machining time (a) Circular Hole; (b) Triangular Hole; (c) Square Hole.

Further analysis of the main effects plot for the Signal-to-Noise ratio (Fig. 11) confirms that the optimal combination of process parameters for achieving the lowest machining time across all three hole geometries corresponds to a cutting speed of 12 m/min, gas pressure of 0.5 bar, focus point of 2 mm, and depth of cut of 3 mm.

**Table 8:** Analysis of variance for machining time.

Source	DF	Circular Hole			Triangular Hole			Square Hole		
		F	*P*	*P*%	F	*P*	*P*%	F	*P*	*P*%
A	2	29.85	0.001	74.385	90.02	0.000	76.19	37.07	0.000	75.43
B	2	0.01	0987	0.032	0.22	0.808	0.186	0.08	0.921	0.169
C	2	0.53	0.616	1.312	1.40	0.318	1.182	0.61	0.576	1.233
D	2	0.49	0.634	1.226	0.12	0.886	0.104	0.79	0.494	1.616
A*D	4	2.13	0.195	10.62	11.22	0.006	18.99	2.79	0.126	11.34
B*D	4	0.21	0.926	1.023	0.12	0.969	0.207	0.23	0.912	0.931
C*D	4	0.79	0.574	3.920	0.35	0.837	0.588	0.78	0.578	3.164
RSE	6						2.539			6.105
Total	26									

Where, A – Cutting Speed, B – Gas Pressure, C – Focus Point, D – Depth of Cut, RSE – Residual Error.

#### Surface hardness

The variation in surface hardness after laser beam machining (LBM) of XG3 maraging steel (Table 9) is a direct outcome of thermal gradients, metallurgical phase transformations, and localized melting/solidification cycles induced by different parameter combinations. The surface hardness, measured in Vickers hardness (Hv), varies significantly across different shapes and parameter settings, and this can be explained based on heat-affected zone (HAZ) behavior, cooling rates, and alloy response under thermal exposure.

Surface hardness increases with increase in cutting speed across all geometries. At 8 m/min, average surface hardness values are lowest (e.g., Trial 3: Circle – 339 Hv, Triangle – 282 Hv, Square – 274 Hv), while at 12 m/min (Trial 21), Circle – 373 Hv, Triangle – 305 Hv, Square – 302 Hv. This is due to shorter laser-material interaction time at higher speeds, which limits the size of the HAZ and causes rapid cooling. Rapid quenching after laser exposure promotes martensitic transformation from the reverted austenite/matrix, increasing surface hardness. In contrast, lower speeds allow prolonged heat input, causing wider HAZ and slower cooling, which results in softer re-solidified structures such as tempered martensite or retained austenite.

Further, gas pressure helps in efficient removal of molten material and influences surface cooling rate. From Trials 1–3 (0.5 bar) to Trials 7–9 (0.9 bar), for same speed and focus, surface hardness shows a decreasing trend. Higher gas pressure improves melt ejection and reduces heat buildup, but it also accelerates surface cooling, leading to increased brittleness and possibly reducing hardness slightly due to finer grain structures. However, too high a pressure may also remove part of the re-solidified layer, slightly reducing the hardened skin thickness, especially in lower-speed conditions.

The optimal focus point increases energy density and concentrates thermal effects. At 6 mm (Trials 13–15), surface hardness values are consistently high: Circle – 366 Hv, Triangle – 301 Hv, Square – 298 Hv. At 2 mm (Trials 1–3), where beam defocus may occur, energy is dispersed over a larger area, reducing peak thermal input and hence hardness (e.g., Trial 3: Circle – 339 Hv). At 4 mm (Trials 25–27), surface hardness is maximum (Trial 25: Circle – 374 Hv), indicating that this setting matches the optical focus of the CO₂ laser with surface depth, resulting in maximum energy absorption, effective surface melting, and fast re-solidification.

Finally, depth of cut has an inverse relation with surface hardness. As depth increases from 3 mm to 9 mm, hardness decreases. Deeper cuts need longer beam exposure, which widens the HAZ and promotes slower cooling. For example, Circle in Trial 1 (3 mm depth) – 355 Hv, and Trial 3 (9 mm depth) – 339 Hv. The same trend follows for triangle and square geometries. This is because deeper penetration causes more heat diffusion into the material bulk, lowering cooling rate and favoring soft phase formation (tempered martensite or retained austenite).

Circular holes consistently show the highest hardness, followed by triangle and square. For example, Trial 25: Circle – 374 Hv, Triangle – 312 Hv, Square – 303 Hv. This is related to thermal stress distribution and laser head movement. In circular geometry, continuous motion and uniform heat application avoid thermal concentration. In contrast, sharp corners in square and triangle geometries lead to localized overheating and slower heat dissipation, which causes uneven cooling and results in softer microstructures at corners.

XG3 steel is a precipitation-hardening maraging steel, rich in Ni, Mo, Co, and Ti. During LBM, localized laser heating causes rapid melting followed by solidification. At high cooling rates (as observed in high-speed or optimal focus trials), martensitic transformation dominates. Martensite in maraging steel is not carbon-driven, but driven by dislocation and phase strain, giving rise to high hardness. Furthermore, at elevated cooling rates, microsegregation is reduced and fine grain structure forms, enhancing the hardness. At lower cooling rates, intermetallic precipitates such as Ni₃Mo or Fe₂Mo may not form efficiently, reducing hardness.

**Table 9:** Experimental results of surface hardness (Hv).

Trial Number	Cutting Speed (m/min)	Gas Pressure (Bar)	Focus Point (mm)	Depth of Cut (mm)	Circle Surface Hardness (Hv)	Triangle Surface Hardness (Hv)	Square Surface Hardness (Hv)
1	8	0.5	2	3	355	295	287
2	8	0.5	2	6	347	289	281
3	8	0.5	2	9	339	282	274
4	8	0.7	4	3	352	293	285
5	8	0.7	4	6	344	286	278
6	8	0.7	4	9	336	284	276
7	8	0.9	6	3	351	292	284
8	8	0.9	6	6	343	285	277
9	8	0.9	6	9	335	279	272
10	10	0.5	4	3	355	304	288
11	10	0.5	4	6	364	300	295
12	10	0.5	4	9	359	296	292
13	10	0.7	6	3	360	306	294
14	10	0.7	6	6	366	301	298
15	10	0.7	6	9	362	302	293
16	10	0.9	2	3	363	308	294
17	10	0.9	2	6	369	303	299
18	10	0.9	2	9	365	302	295
19	12	0.5	6	3	368	311	298
20	12	0.5	6	6	365	306	297
21	12	0.5	6	9	373	305	302
22	12	0.7	2	3	370	308	300
23	12	0.7	2	6	367	306	298
24	12	0.7	2	9	364	304	296
25	12	0.9	4	3	374	312	303
26	12	0.9	4	6	371	310	301
27	12	0.9	4	9	368	306	298

**Fig. 12:**
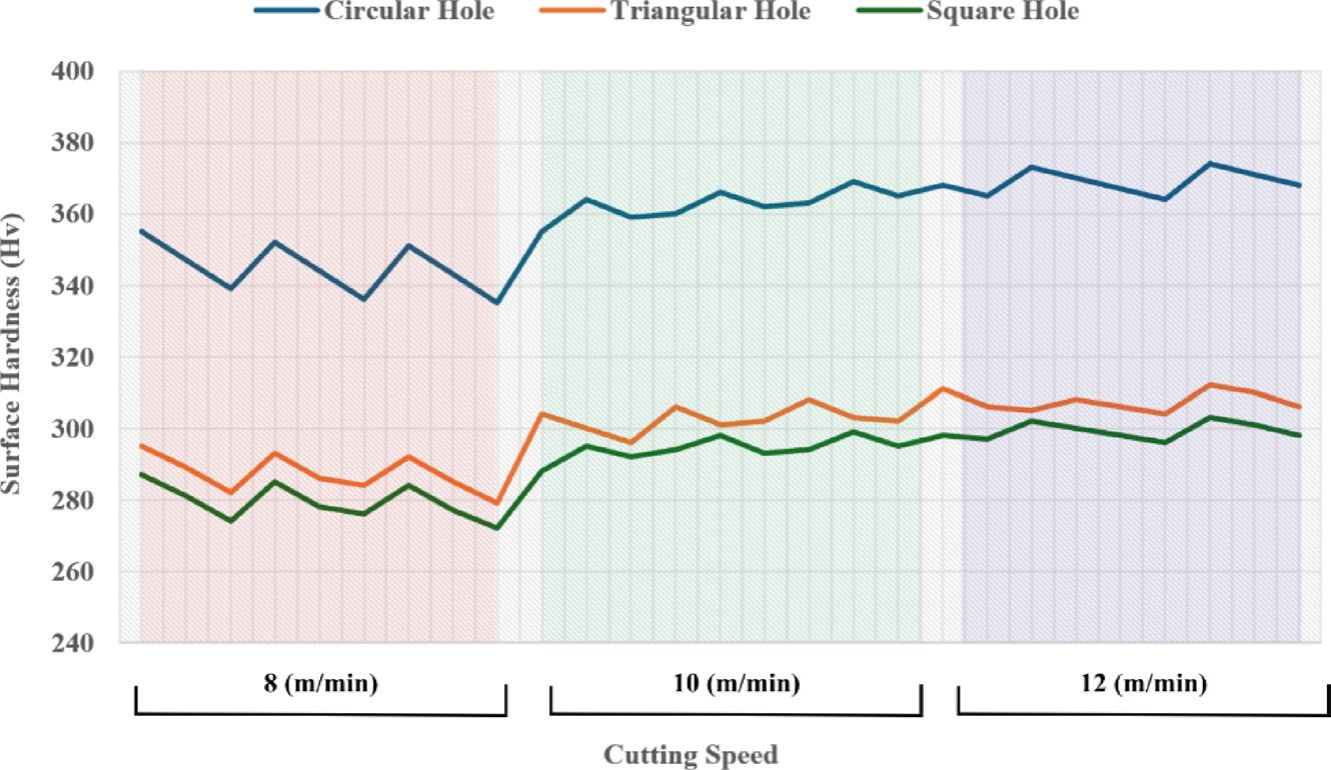
Effect of cutting speed on surface hardness across different hole geometries.

From Fig. 12, it can be understood that, surface hardness measurements in the heat-affected zone reveal significant geometry-dependent behavior, with circular holes achieving hardness values of 335–374 Hv, triangular holes 279–312 Hv, and square holes 272–303 Hv, representing increases of 21–35% relative to the base material hardness (275–280 Hv). The consistent superiority of circular hole hardness over polygonal geometries is attributable to the continuous, uniform laser beam trajectory that prevents thermal concentration at corners, enabling efficient energy distribution and rapid cooling. In contrast, sharp corners in triangular and square profiles interrupt this uniformity, generating localized thermal gradients and differential cooling rates that result in softer microstructures at corner regions. The plots distinctly illustrate that higher cutting speeds (12 m/min) produce marginally elevated hardness compared to lower speeds (8 m/min), likely due to enhanced cooling rates that favor rapid martensitic transformation in the precipitation-hardened XG3 microstructure. The relatively flat hardness response across varying gas pressures and focus points suggests that the thermal cycling rate, predominantly controlled by cutting speed, is the governing variable in determining surface hardening, while the geometry-dependent variations point to the critical role of thermal stress distribution in modulating the final microstructural state and resulting mechanical properties.

**Fig. 13:**
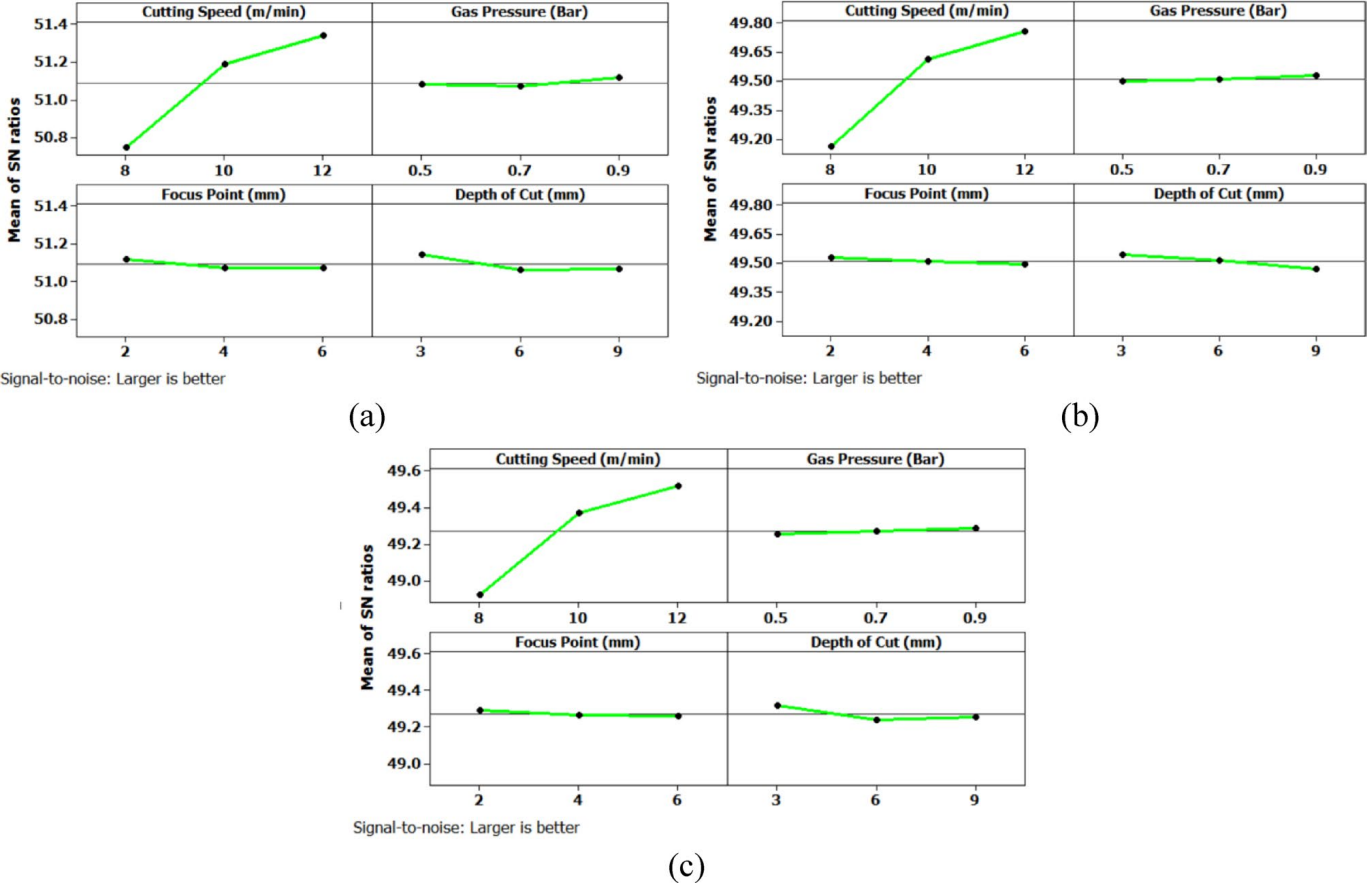
Main Effects plot for surface hardness (a) Circular Hole; (b) Triangular Hole; (c) Square Hole.

The analysis of variance for surface hardness (Table 10) clearly establishes cutting speed as the most significant factor affecting the response across all three hole geometries. It contributes 81.27% for circular holes, 83.49% for triangular holes, and 84.56% for square holes, with highly significant p-values of 0.000 in each case. Other main factors such as gas pressure, focus point, and depth of cut show negligible influence on surface hardness, with percentage contributions below 2% and statistically insignificant p-values well above 0.05. The interaction between cutting speed and depth of cut demonstrates a noticeable effect on surface hardness, particularly for circular and triangular holes where the contribution is 12.29% and 11.76% respectively, both with p-values of 0.019 indicating statistical significance. For square holes, this interaction remains influential with a contribution of 9.642% and a p-value of 0.026. Other interaction terms, including those between gas pressure and depth of cut and between focus point and depth of cut, are found to be statistically insignificant across all geometries, with minimal contributions. The residual standard error is lowest for square holes at 2.353, followed by triangular holes at 2.516 and circular holes at 2.632, indicating a good level of model fit in each case. The main effects plot for surface hardness (Fig. 13), under the larger-the-better condition, shows that the optimal parameter combination is 12 mm/min cutting speed, 0.9 bar gas pressure, 2 mm focus point, and 3 mm depth of cut. The highest cutting speed (12 mm/min) increases thermal input, enhancing surface hardness. Maximum gas pressure (0.9 bar) improves molten material removal and solidification. The smallest focus point (2 mm) concentrates energy, and the least depth of cut (3 mm) limits heat loss, together increasing hardness across all hole geometries.

**Table 10:** Analysis of variance for surface hardness.

Source	DF	Circular Hole			Triangular Hole			Square Hole		
		F	*P*	*P*%	F	*P*	*P*%	F	*P*	*P*%
A	2	92.62	0.000	81.27	99.53	0.000	83.49	107.81	0.000	84.56
B	2	0.58	0.586	0.512	0.20	0.823	0.168	0.21	0.813	0.167
C	2	0.64	0.558	0.565	0.30	0.752	0.25	0.32	0.739	0.249
D	2	2.08	0.206	1.825	1.53	0.291	1.283	2.02	0.213	1.584
A*D	4	7.01	0.019	12.29	7.02	0.019	11.76	6.15	0.026	9.642
B*D	4	0.16	0.952	0.279	0.13	0.967	0.214	0.31	0.862	0.484
C*D	4	0.35	0.835	0.615	0.18	0.941	0.299	0.61	0.671	0.957
RSE	6			2.632	-	-	2.516			2.353
Total	26									

Where, A – Cutting Speed, B – Gas Pressure, C – Focus Point, D – Depth of Cut, RSE – Residual Error.

#### Burr thickness

The burr thickness in laser beam machining of XG3 steel (Table 11) is significantly influenced by the process parameters such as cutting speed, gas pressure, focus point, and depth of cut. An overall trend is observed where burr thickness is highest at lower cutting speeds and decreases as the speed increases. For instance, at 8 m/min cutting speed, the burr thickness for square geometries reaches up to 0.81 mm, whereas at 12 m/min it reduces to as low as 0.42 mm. This reduction is due to the reduced heat input at higher speeds, which limits the volume of molten material and shortens the solidification time, resulting in less material being re-deposited at the edges.

Gas pressure plays a critical role in burr control by assisting the ejection of molten metal from the kerf zone. When the gas pressure is increased from 0.5 to 0.9 bar, a significant decrease in burr thickness is observed. At 0.5 bar, square burr thickness ranges up to 0.81 mm, but at 0.9 bar, it reduces to approximately 0.42 mm. Higher gas.pressure improves the momentum of assist gas, allowing more efficient removal of molten material and reducing its accumulation along the cut edges.

Further, the position of the laser focus point affects energy concentration on the workpiece. Focus points closer to the surface (such as 2 mm) result in more heat-affected zones and higher burr formation due to wider and less intense laser spots. In contrast, optimal focus points like 4 mm and 6 mm produce a finer laser spot with higher energy density, ensuring rapid vaporisation and efficient melt ejection. This is evident in trials with 4 mm and 6 mm focus points, where the burr thickness remains below 0.5 mm even at higher depth of cuts.

Finally, depth of cut directly contributes to burr formation since deeper cuts involve prolonged laser exposure, increasing the melt pool size. This is especially prominent at lower cutting speeds and gas pressures. As the depth of cut increases from 3 mm to 9 mm, burr thickness gradually increases for all geometries. For example, in circular profiles, the burr grows from 0.28 mm to 0.35 mm at 8 m/min and 0.5 bar. However, at optimal settings like 12 m/min, 0.9 bar, and 4 mm focus point, the burr thickness remains nearly constant despite increasing depth of cut, indicating the effectiveness of the chosen parameters.

Among the three geometrical shapes studied, square profiles consistently exhibit the highest burr thickness, followed by triangle and then circle. This is due to the sharp corners and abrupt changes in direction associated with square and triangular geometries, which interrupt the uniform flow of molten metal and gas. Circular geometries facilitate continuous and smooth movement of the laser, leading to uniform heat distribution and better control over molten metal ejection. As a result, the burr formation is lowest in circular cuts under identical machining conditions.

**Table 11:** Experimental results of burr thickness (mm).

Trial Number	Cutting Speed (m/min)	Gas Pressure (Bar)	Focus Point (mm)	Depth of Cut (mm)	Circle Burr Thickness (mm)	Triangle Burr Thickness (mm)	Square Burr Thickness (mm)
1	8	0.5	2	3	0.28	0.51	0.64
2	8	0.5	2	6	0.32	0.58	0.72
3	8	0.5	2	9	0.35	0.64	0.81
4	8	0.7	4	3	0.31	0.56	0.71
5	8	0.7	4	6	0.34	0.62	0.79
6	8	0.7	4	9	0.37	0.67	0.85
7	8	0.9	6	3	0.3	0.54	0.67
8	8	0.9	6	6	0.33	0.6	0.75
9	8	0.9	6	9	0.36	0.65	0.81
10	10	0.5	4	3	0.28	0.41	0.65
11	10	0.5	4	6	0.22	0.48	0.52
12	10	0.5	4	9	0.26	0.52	0.6
13	10	0.7	6	3	0.25	0.41	0.57
14	10	0.7	6	6	0.22	0.45	0.51
15	10	0.7	6	9	0.25	0.46	0.56
16	10	0.9	2	3	0.24	0.38	0.55
17	10	0.9	2	6	0.21	0.42	0.47
18	10	0.9	2	9	0.24	0.44	0.55
19	12	0.5	6	3	0.22	0.33	0.51
20	12	0.5	6	6	0.23	0.4	0.52
21	12	0.5	6	9	0.18	0.42	0.42
22	12	0.7	2	3	0.2	0.36	0.46
23	12	0.7	2	6	0.21	0.38	0.48
24	12	0.7	2	9	0.22	0.39	0.49
25	12	0.9	4	3	0.19	0.34	0.42
26	12	0.9	4	6	0.2	0.36	0.46
27	12	0.9	4	9	0.21	0.37	0.46

**Fig. 14:**
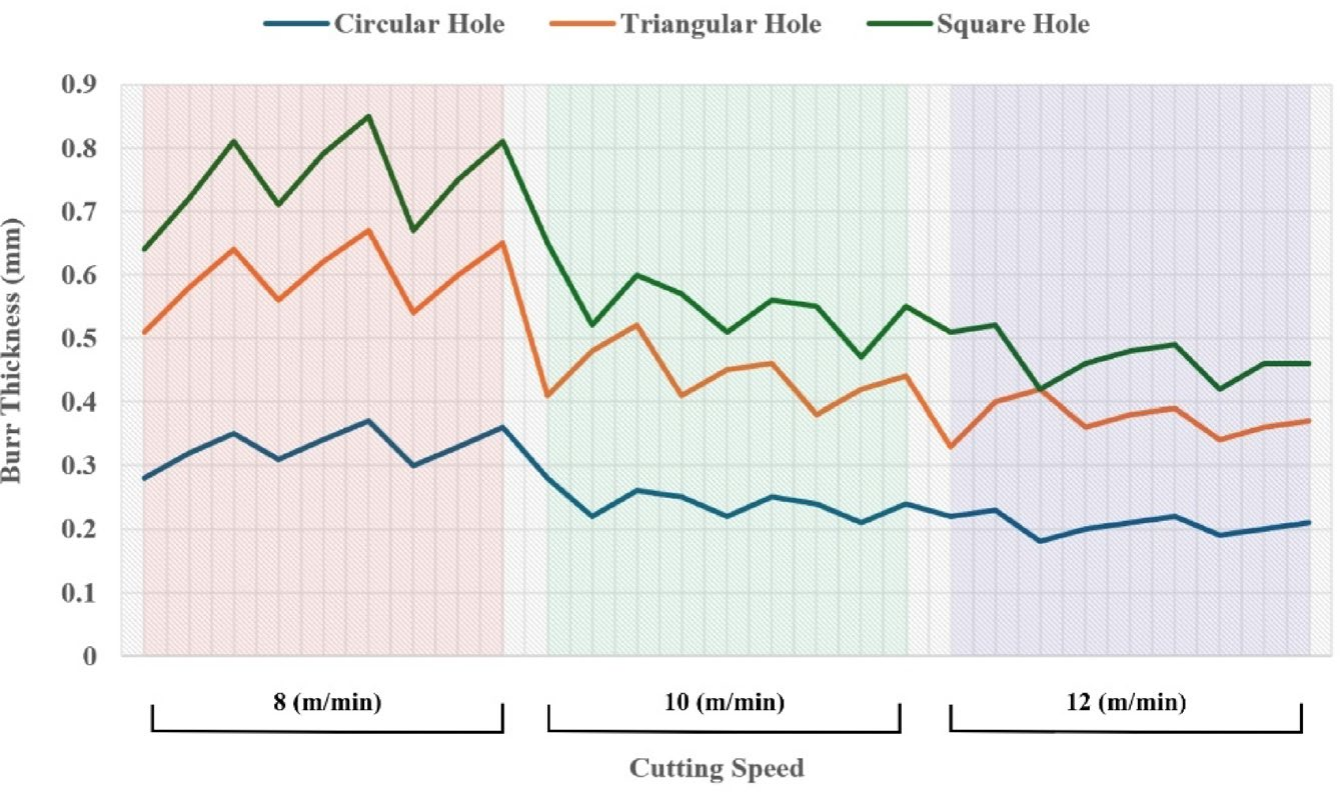
Effect of cutting speed on burr thickness across different hole geometries.

The burr thickness plot [Figure 14] demonstrate a pronounced inverse relationship between cutting speed and burr formation across all three geometric profiles. As cutting speed increases from 8 m/min to 12 m/min, burr thickness decreases substantially, with the most dramatic reduction occurring for square holes where values decline from approximately 0.65–0.85 mm at 8 m/min to 0.42–0.50 mm at 12 m/min. This trend is mechanistically attributable to the reduced thermal input per unit length at higher traverse speeds, which constrains the melt pool volume and shortens the solidification time of ejected molten material at the cut edges. Circular holes consistently exhibit the lowest burr thickness (ranging from 0.18 to 0.37 mm across all speeds), while square holes show the highest (0.42–0.85 mm), reflecting the influence of geometric complexity on melt ejection efficiency. The phenomenon is particularly pronounced at lower cutting speeds (8 m/min) where sustained laser-material interaction creates extensive molten zones prone to re-solidification as burrs. The stabilization of burr thickness at higher speeds (12 m/min) indicates that cutting speed functions as the dominant control mechanism for burr suppression in laser machining of precipitation-hardened maraging steels, effectively overriding the secondary effects of gas pressure and focus position within the tested parameter ranges.

The analysis of variance for burr thickness (Table 12) reveals that cutting speed is the most significant factor influencing burr formation across all three hole geometries. It contributes 84.8% of the total variation in circular holes, 84.68% in triangular holes, and 84.33% in square holes, with all p-values being 0.000, thereby confirming its strong statistical relevance. The interaction between cutting speed and depth of cut (AD) is also notable, accounting for 8.974%, 10.73%, and 9.117% of the variation for circular, triangular, and square holes respectively, and with statistically significant p-values ranging from 0.007 to 0.034. In contrast, the individual effects of gas pressure, focus point, and depth of cut are negligible, each contributing less than 1.5% with p-values far above the 0.05 significance threshold. The remaining interactions such as BD and CD also exhibit minimal influence with low percentage contributions and non-significant p-values.

**Fig. 15:**
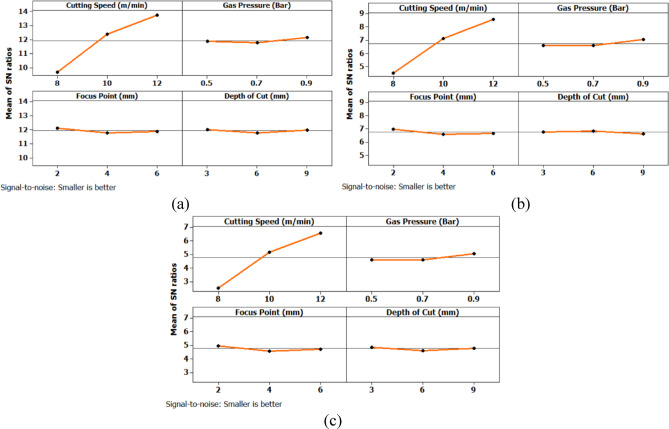
Main Effects plot for burr thickness; (a) Circular Hole; (b) Triangular Hole; (c) Square Hole.

The main effects plot for burr thickness (Fig. 15), considering the smaller-the-better criterion, also indicates that the optimal combination is 12 mm/min cutting speed, 0.9 bar gas pressure, 2 mm focus point, and 3 mm depth of cut. The highest cutting speed minimizes burr formation by enabling rapid material removal and reducing heat diffusion. Maximum gas pressure effectively expels molten material from the cut zone, limiting burr accumulation. A narrow focus point delivers concentrated energy, leading to cleaner cuts, while the shallowest depth of cut reduces thermal damage, further minimizing burr thickness across all hole profiles.

**Table 12:** Analysis of variance for burr thickness.

Source	DF	Circular Hole			Triangular Hole			Square Hole		
		F	*P*	*P*%	F	*P*	*P*%	F	*P*	*P*%
A	2	103.06	0.000	84.8	167.75	0.000	84.68	114.12	0.000	84.33
B	2	0.84	0.477	0.689	2.60	0.154	1.311	2.02	0.214	1.49
C	2	0.74	0.516	0.608	1.78	0.247	0.9	1.04	0.411	0.764
D	2	0.44	0.663	0.362	0.39	0.695	0.195	0.40	0.688	0.294
A*D	4	5.45	0.034	8.974	10.63	0.007	10.73	6.17	0.026	9.117
B*D	4	0.26	0.892	0.431	0.18	0.941	0.181	0.38	0.819	0.555
C*D	4	1.01	0.472	1.658	0.47	0.755	0.478	0.83	0.551	1.229
RSE	6			2.468			1.514			2.216
Total	26									

Where, A – Cutting Speed, B – Gas Pressure, C – Focus Point, D – Depth of Cut, RSE – Residual Error.

### Multi-Objective optimization analysis using Pareto fronts

In the present study, the optimization of laser cutting parameters for three distinct profile geometries i.e. circle, square, and triangle was performed using a multi-objective genetic algorithm (MOGA). To effectively model the responses as functions of the process parameters, second-order regression equations (Eq. 10 – Eq. 21) were developed for each profile shape. The input parameters considered were Cutting Speed (x₁, m/min), Gas Pressure (x₂, Bar), Focus Point (x₃, mm), and Depth of Cut (x₄, mm). The regression models captured both main effects and interaction effects of the variables on each output response, providing accurate predictive capabilities.

10 $$\begin{aligned}&\:Circle\:Surface\:Roughness\:=20.57-1.645\mathrm{*}x1-5.50\mathrm{*}x2+0.751\mathrm{*}x3+0.124\mathrm{*}x4\\&+0.0829\mathrm{*}x1\mathrm{*}x1+2.74\mathrm{*}x2\mathrm{*}x2-0.0049\mathrm{*}x3\mathrm{*}x3+0.0085\mathrm{*}x4\mathrm{*}x4\\&+0.053\mathrm{*}x1\mathrm{*}x2-0.0614\mathrm{*}x1\mathrm{*}x3-0.0197\mathrm{*}x1\mathrm{*}x4+0.099\mathrm{*}x2\mathrm{*}x4-0.0151\mathrm{*}x3\mathrm{*}x4\end{aligned}$$

11 $$\begin{aligned}&\:Circle\:Machining\:Time\:=\:8.49-1.186*x1-4.14*x2+0.684*x3+0.075*x4\\&+0.061*x1*x12.04*x2*x2-0.0229*x3*x3+0.0058*x4*x4+0.014*x1*x2\\&-0.0422*x1*x3-0.01556*x1*x4+0.1264*x2*x4-0.01097*x3*x4\end{aligned}$$

12 $$\begin{aligned}&\:Circle\:Surface\:Hardness\:=\:226.4+27.17*x1+12.5*x2-11.22*x3-4.21*x4\\&-1.444*x1*x1-6.9*x2*x2+0.319*x3*x3-0.142*x4*x4+1.94*x1*x2\\&+0.750*x1*x3+0.569*x1*x4-1.81*x2*x4+0.153*x3*x4\end{aligned}$$

13 $$\begin{aligned}&\:Circle\:Burr\:Thickness\:=\:0.828-0.13*x1+0.264*x2+0.0414*x3+0.0155*x4+0.00667*x1*x1-0.111*x2*x2\\&-0.00306*x3*x3+0.000926*x4*x4-0.0194*x1*x2-0.00111*x1*x3\\&-0.002639*x1*x4+0.00972*x2*x4-0.000972*x3*x4\end{aligned}$$

14 $$\begin{aligned}&\:Triangle\:Surface\:Roughness\:=31.97-3.193*x1-3.17*x2+0.918*x3+0.511*x4+0.1538*x1*x1+2.46*x2*x2\\&-0.0163*x3*x3-0.00667*x4*x4+0.042*x1*x2-0.0750*x1*x3\\&-0.01278*x1*x4-0.2167*x2*x4-0.00597*x3*x4\end{aligned}$$

15 $$\begin{aligned}&\:Triangle\:Machining\:Time\:=13.54-2.230*x1-1.75*x2+0.881*x3+0.3639*x4+0.1067*x1*x1\\&+1.58*x2*x2-0.0433*x3*x3-0.00593*x4*x4-0.033*x1*x2\\&-0.0525*x1*x3-0.00819*x1*x4-0.1264*x2*x4+0.00014*x3*x4\end{aligned}$$

16 $$\begin{aligned}&\:Triangle\:Surface\:Hardness\:=172.6+25.14*x1+30.6*x2-7.58*x3-5.37*x4\\&-1.264*x1*x1-26.4*x2*x2+0.097*x3*x3+0.1049*x4*x4\\&+1.39*x1*x2+0.667*x1*x3+0.2639*x1*x4+0.278*x2*x4\end{aligned}$$

17 $$\begin{aligned}&\:Triangle\:Burr\:Thickness\:=1.301-0.2228*x1+0.597*x2\\&+0.0703*x3+0.06542*x4+0.01083*x1*x1-0.167*x2*x2-0.00583*x3*x3-0.001111*x4*x4\\&-0.0333*x1*x2-0.00250*x1*x3-0.002778*x1*x4-0.01806*x2*x4+0.000417*x3*x4\end{aligned}$$

18 $$\begin{aligned}&\:Square\:Surface\:Roughness\:=36.72-3.362*x1-5.6*x2+1.137*x3+0.139*x4+0.1637*x1*x1\\&+2.32*x2*x2-0.0236*x3*x3+0.0187*x4*x4\\&+0.094*x1*x2-0.0797*x1*x3-0.0317*x1*x4\\&+0.15*x2*x4-0.0239*x3*x4\end{aligned}$$

19 $$\begin{aligned}&\:Square\:Machining\:Time\:=16.38-2.446*x1-4.7*x2+1.203*x3+0.2*x4+0.1243*x1*x1\\&+2.18*x2*x2-0.0506*x3*x3+0.011*x4*x4-0.039*x1*x2\\&-0.065*x1*x3-0.0319*x1*x4+0.213*x2*x4-0.0222*x3*x4\end{aligned}$$

20 $$\begin{aligned}&\:Square\:Surface\:Hardness\:=159+23.86*x1+44.7*x2\\&-8.14*x3-2.74*x4-1.236*x1*x1-29.2*x2*x2+0.167*x3*x3\\&-0.105*x4*x4+1.39*x1*x2+0.611*x1*x3+0.403*x1*x4-1.53*x2*x4+0.097*x3*x4\end{aligned}$$

21 $$\begin{aligned}&\:Square\:Burr\:Thickness\:=1.606-0.2622*x1+0.89*x2+0.1003*x3+0.045*x4+0.01403*x1*x1\\&-0.347*x2*x2-0.00903*x3*x3+0.00179*x4*x4-0.0611*x1*x2\\&-0.00167*x1*x3-0.00653*x1*x4+0.0208*x2*x4-0.00222*x3*x4\end{aligned}$$

A population size of 250 was selected for the genetic algorithm, and the tournament selection strategy was employed to maintain diversity. The optimization process was constrained to 200 generations with a crossover fraction of 0.45. Advanced evolutionary operators were applied, such as the gacreationsobol function for population initialization, crossoverlaplace for recombination, and the mutationadaptfeasible function for adaptive mutation. The decision variable bounds were set as follows: Cutting Speed =^8–12^ m/min, Gas Pressure = [0.5–0.9] Bar, Focus Point =^2–6^ mm, and Depth of Cut =^3–9^ mm. These bounds were selected based on process feasibility and preliminary trials.

**Fig. 16:**
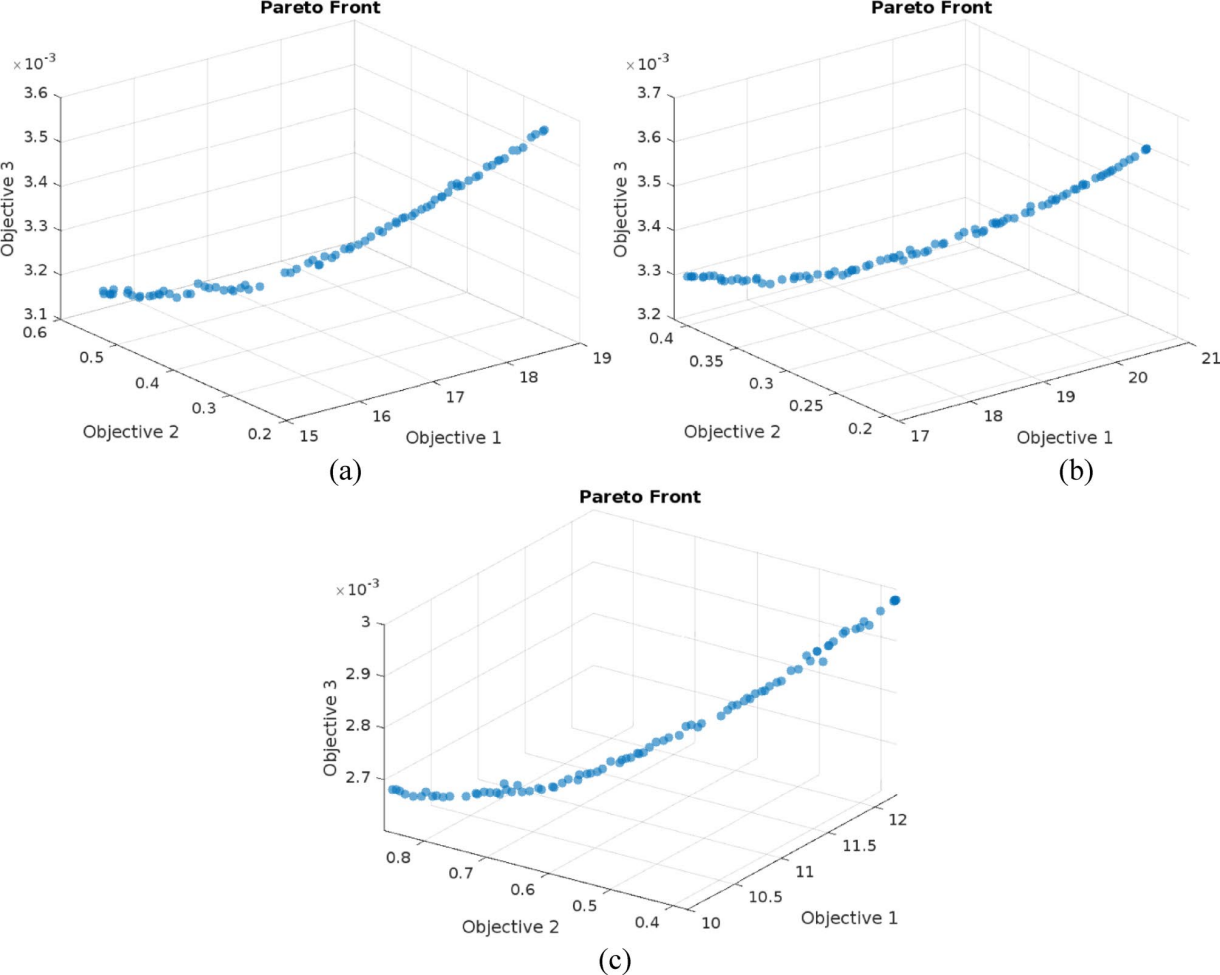
Pareto plots for the LASER Beam Machined holes; (a) Circular hole; (b) Triangular hole; (c) Square hole.

The optimization yielded Pareto fronts for each profile geometry (shown in Fig. 16a and c), clearly illustrating the trade-offs between the objectives. For the circular profile, the optimal parameter setting was identified as [10.0833 m/min, 0.8636 Bar, 0.0027 mm, 0.7458 mm], resulting in a balanced compromise among all objectives. The corresponding top Pareto-optimal solutions featured surface roughness values ranging from 1.10 to 1.16 μm, machining times between 2.44 and 2.52 s, surface hardness around 233 HV, and minimal burr thickness near 0.78 mm.

In the case of the square profile, the optimal parameter set was [17.1763 m/min, 0.4136 Bar, 0.0033 mm, 0.1711 mm], producing higher surface roughness and burr thickness compared to the circular counterpart but yielding increased machining hardness (approx. 167 HV) and acceptable processing times around 3.5 s. For the triangular profile, the optimal solution occurred at [15.1085 m/min, 0.5393 Bar, 0.0032 mm, 0.2908 mm], demonstrating moderate performance across all criteria, with surface roughness between 1.15 and 1.23 μm, machining time from 2.96 to 3.04 s and burr thickness close to 1.29 mm.

The analysis confirmed that profile geometry plays a significant role in laser-material interaction, influencing thermal distribution, molten material ejection, and cut quality. The regression models, in conjunction with the genetic algorithm, effectively mapped and explored the multi-objective design space, enabling a systematic determination of optimal processing conditions.

#### Model performance outside the experimental range (Extrapolation)

To address the model’s robustness, its predictive behavior was tested for input parameter values outside the original experimental domain. While the trained BPANN model can mathematically generate predictions for these extrapolated points, these predictions must be interpreted with extreme caution. A machine learning model is only validated within the data space on which it was trained. Outside this domain, the underlying physical phenomena of the LBM process may change drastically. For example, at a cutting speed of 14 m/min and a depth of 9 mm, the laser’s energy input may become insufficient to achieve a full cut, leading to a catastrophic failure of the process. Since the BPANN model has not been trained on data representing these failure modes, its extrapolated predictions are not physically reliable. Therefore, while the BPANN model is a highly accurate *interpolation* tool within the tested parameter ranges, its use for *extrapolation* is not recommended without further experimental validation.

### Prediction of response variables

Prediction of response variables was performed using two prominent modeling techniques: Response Surface Methodology (RSM) and Backpropagation Artificial Neural Network (BPANN). While RSM provides a mathematical approximation based on second-order polynomial equations, BPANN employs a data-driven approach capable of capturing complex nonlinear relationships. These models were trained and validated using experimental data to estimate key output responses, enabling accurate forecasting and deeper understanding of the influence of process parameters.

#### Response surface methodology

Based on the regression equations (Eq. 22 – Eq. 33) developed using the estimated regression coefficients, the prediction of output responses was carried out within the defined range of input parameters and their respective levels. To ensure the reliability and statistical validity of the regression models, Analysis of Variance (ANOVA) was employed as a validation tool. The F-values obtained from the ANOVA for all cases (as shown in Tables 13, 14, 15 and 16) were found to be greater than the corresponding critical F-value from the Fischer distribution Table (2.48) at a 95% confidence level. This indicates that the regression models are statistically significant, meaning the variation in the output parameters is adequately explained by the variation in the input parameters, and not due to random chance. Therefore, the developed models can be confidently used for predicting the output responses within the specified experimental domain.

**Fig. 17:**
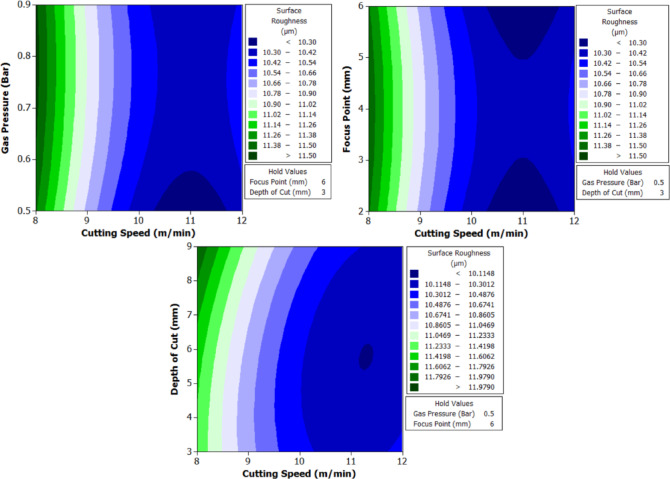
Contour plots for surface roughness of circular hole.

**Fig. 18:**
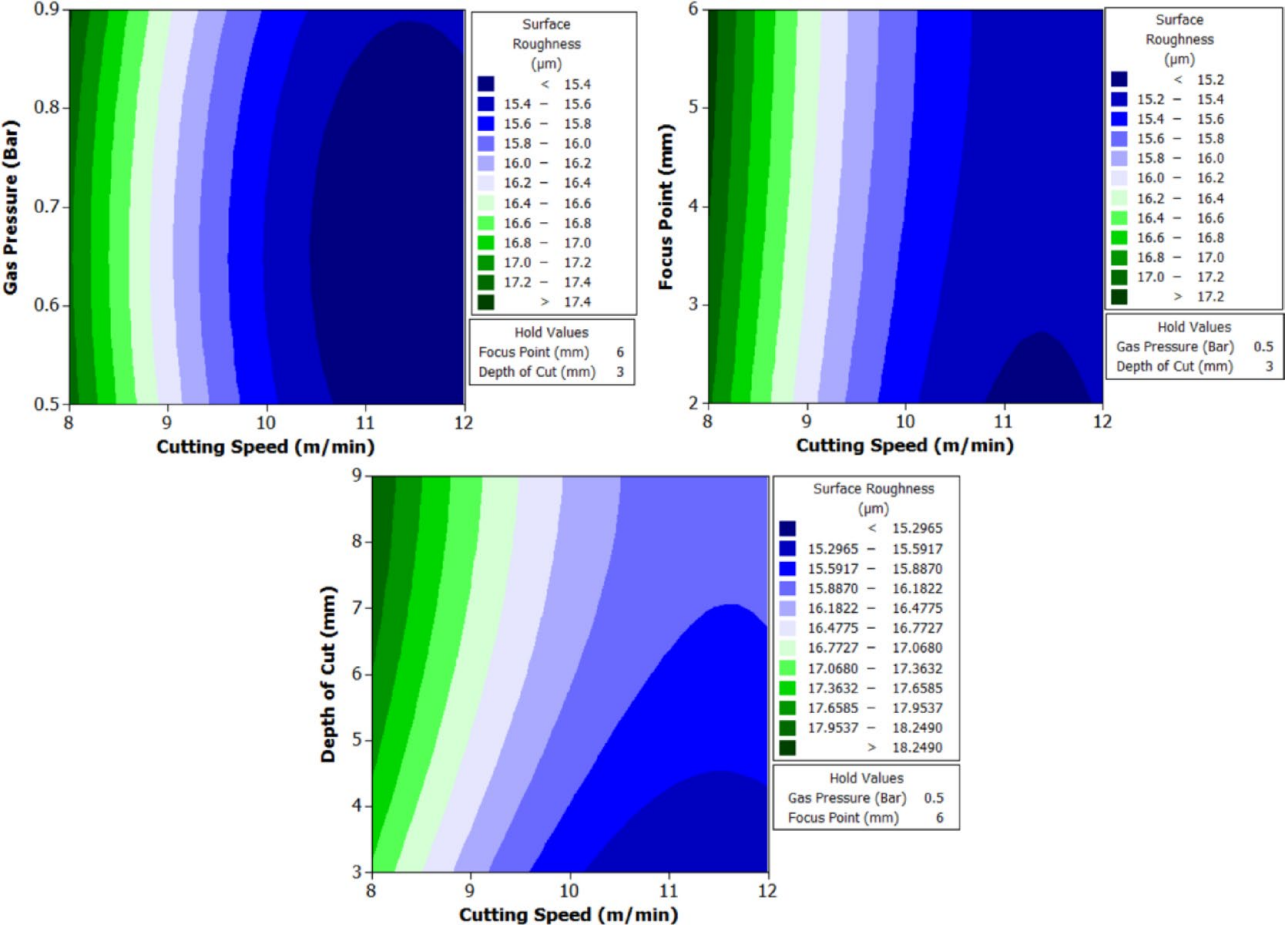
Contour plots for surface roughness of triangular hole.

**Fig. 19:**
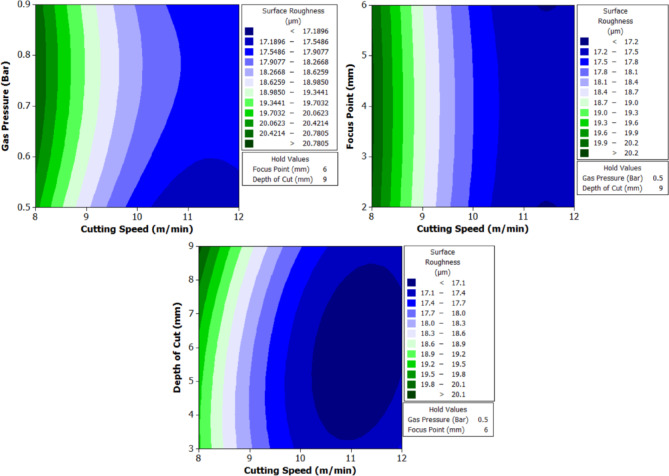
Contour plots for surface roughness of square hole.

Figures 17, 18 and 19 present contour plots illustrating the influence of input process parameters on four key output responses (surface roughness, machining time, surface hardness, and burr thickness) across three distinct geometries (circular, triangular and square). For each profile, the interaction between input variables is visualized to understand how their combined effect governs the response behavior.

22 $$\begin{aligned}&\:\mathrm{S}\mathrm{u}\mathrm{r}\mathrm{f}\mathrm{a}\mathrm{c}\mathrm{e}\:\mathrm{r}\mathrm{o}\mathrm{u}\mathrm{g}\mathrm{h}\mathrm{n}\mathrm{e}\mathrm{s}\mathrm{s}\:\left(\mathrm{C}\mathrm{i}\mathrm{r}\mathrm{c}\mathrm{u}\mathrm{l}\mathrm{a}\mathrm{r}\:\mathrm{H}\mathrm{o}\mathrm{l}\mathrm{e}\right)\:=\:24.0534-2.73709\mathrm{A}\:+\:3.01585\mathrm{B}\:\\&+\:0.168485\mathrm{C}+0.0759575\mathrm{D}+0.128939\mathrm{A}2\:-1.85606\mathrm{B}2\:-0.0210606\mathrm{C}2\:\\&+\:0.0173064\mathrm{D}2\:-\:0.034375\mathrm{A}\mathrm{B}\:-\:0.0272917\mathrm{A}\mathrm{D}\:+0.0645833\mathrm{B}\mathrm{D}\end{aligned}$$

23 $$\begin{aligned}&\:\mathrm{S}\mathrm{u}\mathrm{r}\mathrm{f}\mathrm{a}\mathrm{c}\mathrm{e}\:\mathrm{r}\mathrm{o}\mathrm{u}\mathrm{g}\mathrm{h}\mathrm{n}\mathrm{e}\mathrm{s}\mathrm{s}\:\left(\mathrm{T}\mathrm{r}\mathrm{i}\mathrm{a}\mathrm{n}\mathrm{g}\mathrm{u}\mathrm{l}\mathrm{a}\mathrm{r}\:\mathrm{H}\mathrm{o}\mathrm{l}\mathrm{e}\right)\:=\:35.9635-3.7771\mathrm{A}-3.27547\mathrm{B}\\&+0.290586\mathrm{C}+0.560043\mathrm{D}+0.168939\mathrm{A}2+3.14394\mathrm{B}2-0.00981061\mathrm{C}2-0.0182492\mathrm{D}2\:+0.0015625\mathrm{A}\mathrm{B}\\&+0.00953123\mathrm{A}\mathrm{C}-0.0128125\mathrm{A}\mathrm{D}-0.0953125\mathrm{B}\mathrm{C}-0.090625\mathrm{B}\mathrm{D}-0.00635417\mathrm{C}\mathrm{D}\end{aligned}$$

24 $$\begin{aligned}&\:\mathrm{S}\mathrm{u}\mathrm{r}\mathrm{f}\mathrm{a}\mathrm{c}\mathrm{e}\:\mathrm{r}\mathrm{o}\mathrm{u}\mathrm{g}\mathrm{h}\mathrm{n}\mathrm{e}\mathrm{s}\mathrm{s}\:\left(\mathrm{S}\mathrm{q}\mathrm{u}\mathrm{a}\mathrm{r}\mathrm{e}\:\mathrm{H}\mathrm{o}\mathrm{l}\mathrm{e}\right)\:=\:42.6501-5.34894\mathrm{A}+10.496\mathrm{B}3+0.173939\mathrm{C}\\&+0.073992\mathrm{D}+0.25075\mathrm{A}2-7.79924\mathrm{B}2-0.0217424\mathrm{C}2+0.0325589\mathrm{D}2+0.04875\mathrm{A}\mathrm{B}-0.046875\mathrm{A}\mathrm{D}+0.135417\mathrm{B}\mathrm{D}\end{aligned}$$

**Table 13:** ANOVA for response surface methodology of surface roughness.

Source	DF	Circular Hole		Triangular Hole		Square Hole	
		F	*P*	F	*P*	F	*P*
Regression	14	22.58	0.000	73.96	0.000	29.72	0.000
Residual Error	14	-	-	-	-	-	-
Total	29	-	-	-	-	-	-

The contour plots in Fig. 17 reveal that minimum surface roughness (below 10.30 μm) is achieved at higher cutting speeds, specifically between 10 and 12 m/min, combined with a gas pressure range of 0.5 to 0.9 Bar. The negative coefficient of cutting speed (A) in Eq. 22 (−2.73709 A) supports this, indicating that an increase in speed generally reduces roughness. The interaction between cutting speed and focus point also shows that lower roughness is favored at higher speeds and a mid-range focus point of approximately 4–5 mm. Further, for triangular holes (Fig. 18), a similar trend is observed where higher cutting speeds lead to lower surface roughness. The lowest roughness values (below 15.2 μm) are found at cutting speeds exceeding 10 m/min. Equation 23 corroborates this with a significant negative coefficient for cutting speed (−3.7771 A). The effect of gas pressure is also pronounced, with lower pressures generally yielding a smoother surface. Finally, the surface roughness for square holes, as depicted in Fig. 19, is minimized at high cutting speeds (above 10 m/min) and high gas pressures (above 0.8 Bar). Equation 24 shows that cutting speed (A) has the most substantial negative linear effect (−5.34894 A), while gas pressure (B) has a strong positive effect (+ 10.496B). This suggests that while higher speed is beneficial, gas pressure must be carefully controlled to achieve optimal surface quality.

25 $$\begin{aligned}&\:\mathrm{M}\mathrm{a}\mathrm{c}\mathrm{h}\mathrm{i}\mathrm{n}\mathrm{i}\mathrm{n}\mathrm{g}\:\mathrm{t}\mathrm{i}\mathrm{m}\mathrm{e}\:\left(\mathrm{C}\mathrm{i}\mathrm{r}\mathrm{c}\mathrm{u}\mathrm{l}\mathrm{a}\mathrm{r}\:\mathrm{H}\mathrm{o}\mathrm{l}\mathrm{e}\right)\:\:=\:11.7297-2.06638\mathrm{A}+1.96913\mathrm{B}+0.134545\mathrm{C}\\&+0.00263678\mathrm{D}+0.0981818\mathrm{A}2-1.55682\mathrm{B}2-0.0168182\mathrm{C}2+0.0136364\mathrm{D}2\\&-0.009375\mathrm{A}\mathrm{B}-0.0210417\mathrm{A}\mathrm{D}+0.127083\mathrm{B}\mathrm{D}\end{aligned}$$

26 $$\begin{aligned}&\:\mathrm{M}\mathrm{a}\mathrm{c}\mathrm{h}\mathrm{i}\mathrm{n}\mathrm{i}\mathrm{n}\mathrm{g}\:\mathrm{t}\mathrm{i}\mathrm{m}\mathrm{e}\:\left(\mathrm{T}\mathrm{r}\mathrm{i}\mathrm{a}\mathrm{n}\mathrm{g}\mathrm{u}\mathrm{l}\mathrm{a}\mathrm{r}\:\mathrm{H}\mathrm{o}\mathrm{l}\mathrm{e}\right)\:=\:16.2481-2.55969\mathrm{A}-2.11095\mathrm{B}+0.144515\mathrm{C}+0.435325\mathrm{D}\\&+0.113409\mathrm{A}2+2.21591\mathrm{B}2-0.00284091\mathrm{C}2-0.0179293\mathrm{D}2-0.0484375\mathrm{A}\mathrm{B}\\&-0.00546875\mathrm{A}\mathrm{C}-0.0065625\mathrm{A}\mathrm{D}-0.0546875\mathrm{B}\mathrm{C}-0.0197917\mathrm{B}\mathrm{D}-0.00364583\mathrm{C}\mathrm{D}\end{aligned}$$

27 $$\begin{aligned}&\:\mathrm{M}\mathrm{a}\mathrm{c}\mathrm{h}\mathrm{i}\mathrm{n}\mathrm{i}\mathrm{n}\mathrm{g}\:\mathrm{t}\mathrm{i}\mathrm{m}\mathrm{e}\:\left(\mathrm{S}\mathrm{q}\mathrm{u}\mathrm{a}\mathrm{r}\mathrm{e}\:\mathrm{H}\mathrm{o}\mathrm{l}\mathrm{e}\right)\:=\:22.9084-4.03151\mathrm{A}+4.31604\mathrm{B}\\&+0.255758\mathrm{C}-0.000420875\mathrm{D}+0.19053\mathrm{A}2-3.69697\mathrm{B}2-0.0319697\mathrm{C}2\\&+0.0307912\mathrm{D}2\:+0.01875\mathrm{A}\mathrm{B}-0.0425\mathrm{A}\mathrm{D}+0.216667\mathrm{B}\mathrm{D}\end{aligned}$$

**Table 14:** ANOVA for response surface methodology of machining time.

Source	DF	Circular Hole		Triangular Hole		Square Hole	
		F	*P*	F	*P*	F	*P*
Regression	14	22.58	0.000	58.81	0.000	22.02	0.000
Residual Error	14	-	-	-	-	-	-
Total	29	-	-	-	-	-	-

**Fig. 20:**
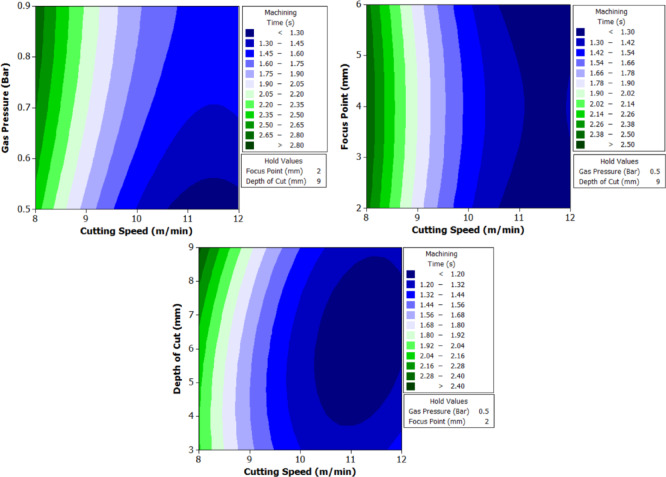
Contour plots for machining time of circular hole.

**Fig. 21:**
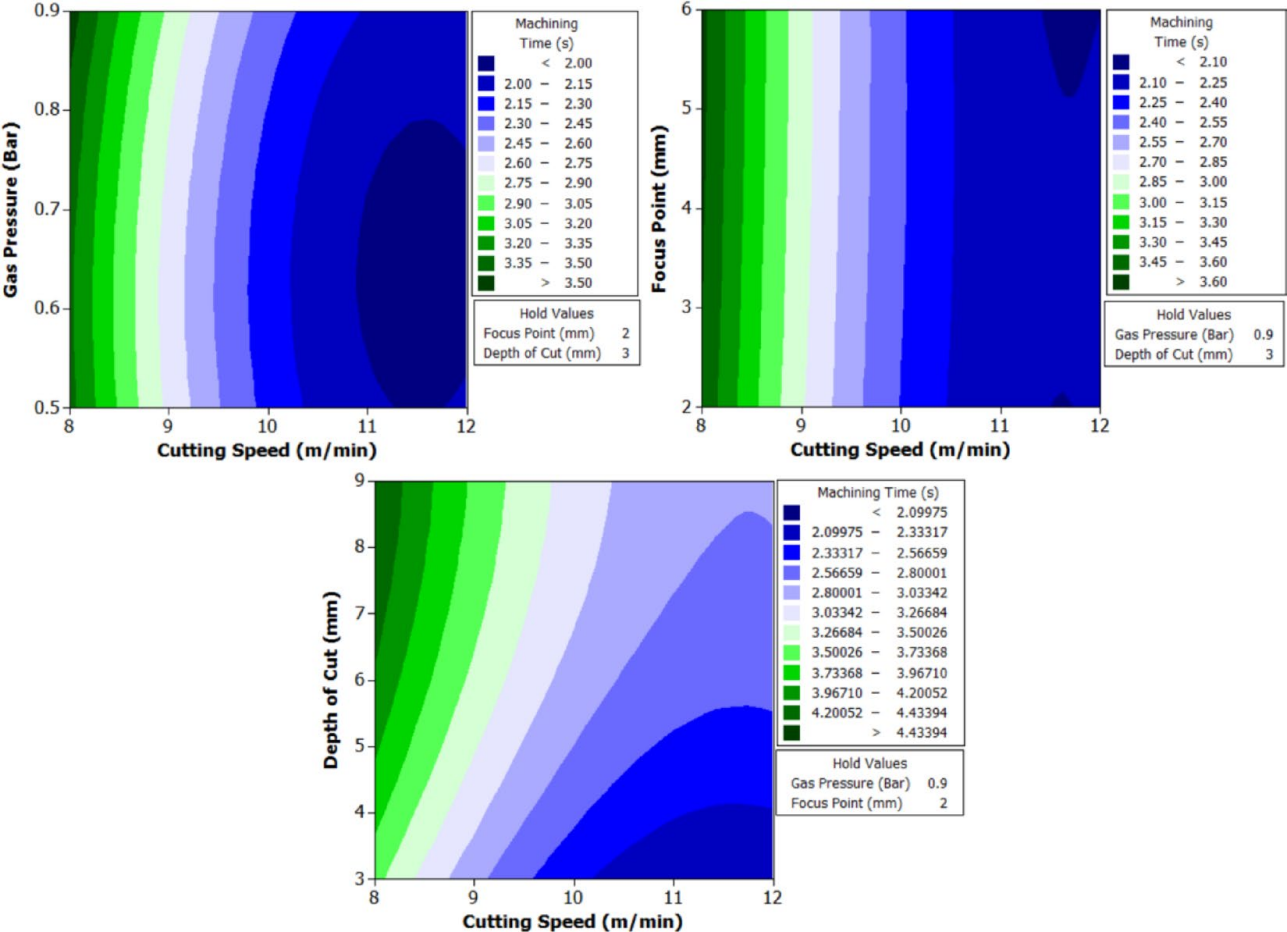
Contour plots for machining time of triangular hole.

**Fig. 22:**
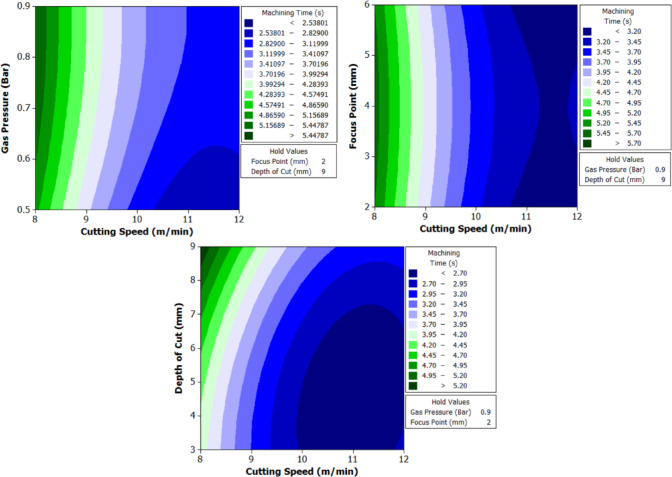
Contour plots for machining time of square hole.

The machining time is a crucial productivity indicator, and the developed models for all hole shapes were found to be highly significant. As expected, cutting speed was the most dominant factor influencing the time taken for machining. Across all three geometries, the contour plots (Figs. 20, 21 and 22) illustrate a clear and strong inverse relationship between cutting speed and machining time. The shortest machining times are consistently achieved at the maximum cutting speed of 12 m/min. This observation is mathematically confirmed by the large negative coefficients for the cutting speed parameter (A) in the regression equations for circular (−2.06638 A), triangular (−2.55969 A), and square holes (−4.03151 A). Other parameters like gas pressure, focus point, and depth of cut show a much less significant impact on machining time compared to cutting speed.

28 $$\begin{aligned}&\:\mathrm{S}\mathrm{u}\mathrm{r}\mathrm{f}\mathrm{a}\mathrm{c}\mathrm{e}\:\mathrm{h}\mathrm{a}\mathrm{r}\mathrm{d}\mathrm{n}\mathrm{e}\mathrm{s}\mathrm{s}\:\left(\mathrm{C}\mathrm{i}\mathrm{r}\mathrm{c}\mathrm{u}\mathrm{l}\mathrm{a}\mathrm{r}\:\mathrm{H}\mathrm{o}\mathrm{l}\mathrm{e}\right)\:\:=\:160.942+44.381\mathrm{A}-94.8295\mathrm{B}-3.45455\mathrm{C}-1.67572\mathrm{D}\\&-2.19318\mathrm{A}2+55.6818\mathrm{B}2+0.431818\mathrm{C}2-0.363636\mathrm{D}2+2.8125\mathrm{A}\mathrm{B}+0.645833\mathrm{A}\mathrm{D}-2.29167\mathrm{B}\mathrm{D}\end{aligned}$$

29 $$\begin{aligned}&\:\mathrm{S}\mathrm{u}\mathrm{r}\mathrm{f}\mathrm{a}\mathrm{c}\mathrm{e}\:\mathrm{h}\mathrm{a}\mathrm{r}\mathrm{d}\mathrm{n}\mathrm{e}\mathrm{s}\mathrm{s}\:\left(\mathrm{T}\mathrm{r}\mathrm{i}\mathrm{a}\mathrm{n}\mathrm{g}\mathrm{u}\mathrm{l}\mathrm{a}\mathrm{r}\:\mathrm{H}\mathrm{o}\mathrm{l}\mathrm{e}\right)\:=\:157.161+29.8756\mathrm{A}-18.8636\mathrm{B}+0.202336\mathrm{C}-7.08965\mathrm{D}\\&-1.37121\mathrm{A}2+0.378788\mathrm{B}2-0.121212\mathrm{C}2+0.279461\mathrm{D}2+1.875\mathrm{A}\mathrm{B}+0.03125\mathrm{A}\mathrm{C}+0.25\mathrm{A}\mathrm{D}\\&+0.3125\mathrm{B}\mathrm{C}-0.416667\mathrm{B}\mathrm{D}+0.0208333\mathrm{C}\mathrm{D}\end{aligned}$$

30 $$\begin{aligned}&\:\mathrm{S}\mathrm{u}\mathrm{r}\mathrm{f}\mathrm{a}\mathrm{c}\mathrm{e}\:\mathrm{h}\mathrm{a}\mathrm{r}\mathrm{d}\mathrm{n}\mathrm{e}\mathrm{s}\mathrm{s}\:\left(\mathrm{S}\mathrm{q}\mathrm{u}\mathrm{a}\mathrm{r}\mathrm{e}\:\mathrm{H}\mathrm{o}\mathrm{l}\mathrm{e}\right)\:=\:107.574+36.5234\mathrm{A}-23.649\mathrm{B}-2.78788\mathrm{C}-0.673401\mathrm{D}\\&-1.77652\mathrm{A}2+9.84848\mathrm{B}2+0.348485\mathrm{C}2-0.345118\mathrm{D}2+1.875\mathrm{A}\mathrm{B}+0.5\mathrm{A}\mathrm{D}+1.66667\mathrm{B}\mathrm{D}\end{aligned}$$

**Table 15:** ANOVA for response surface methodology of surface hardness.

Source	DF	Circular Hole		Triangular Hole		Square Hole	
		F	*P*	F	*P*	F	*P*
Regression	14	37.85	0.000	108.27	0.000	49.06	0.000
Residual Error	14	-	-	-	-	-	-
Total	29	-	-	-	-	-	-

**Fig. 23:**
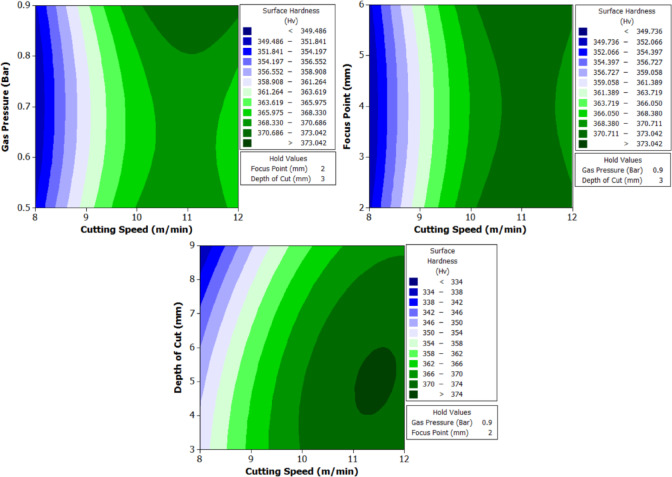
Contour plots for surface hardness of circular hole.

**Fig. 24:**
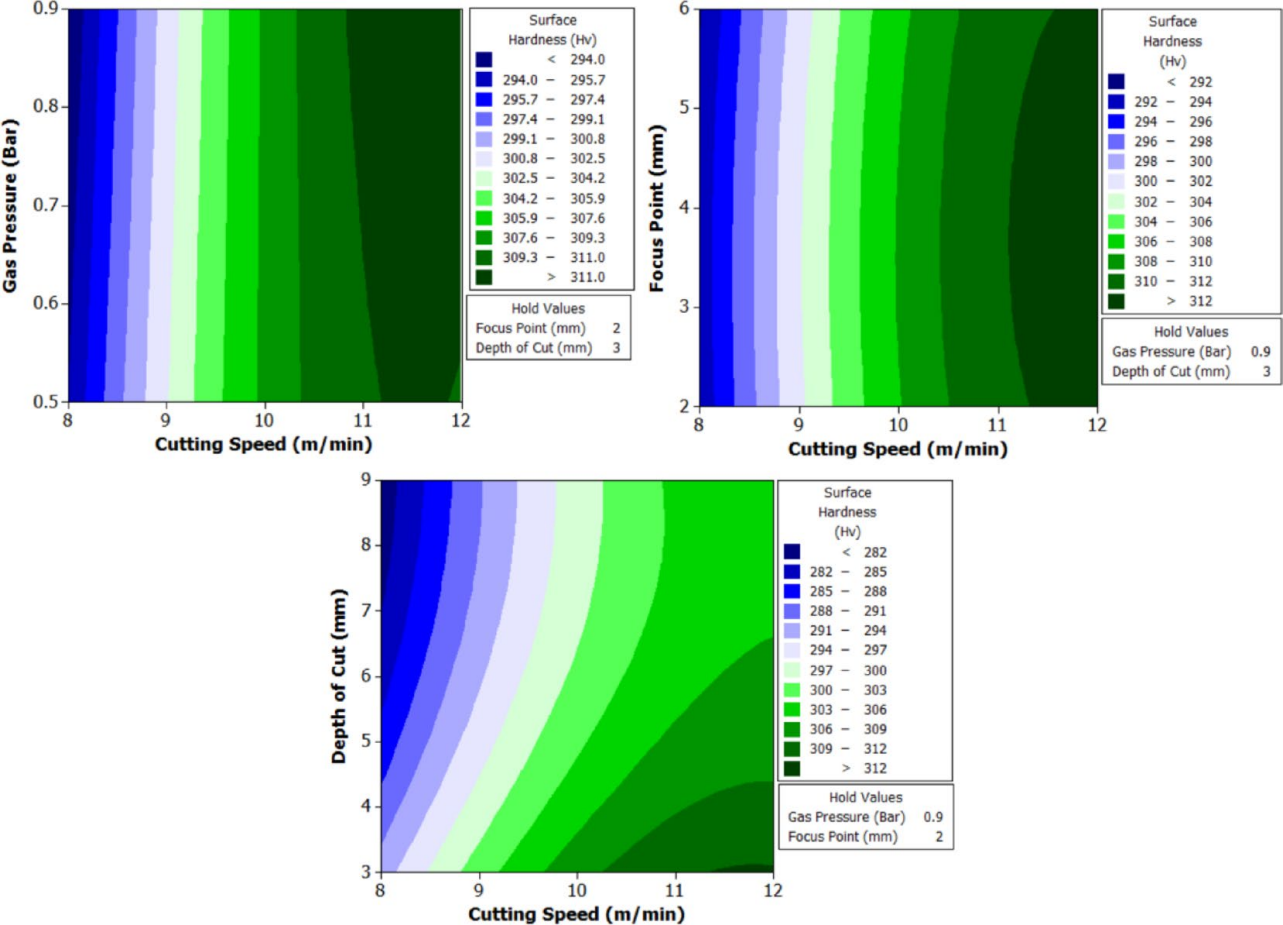
Contour plots for surface hardness of triangular hole.

**Fig. 25:**
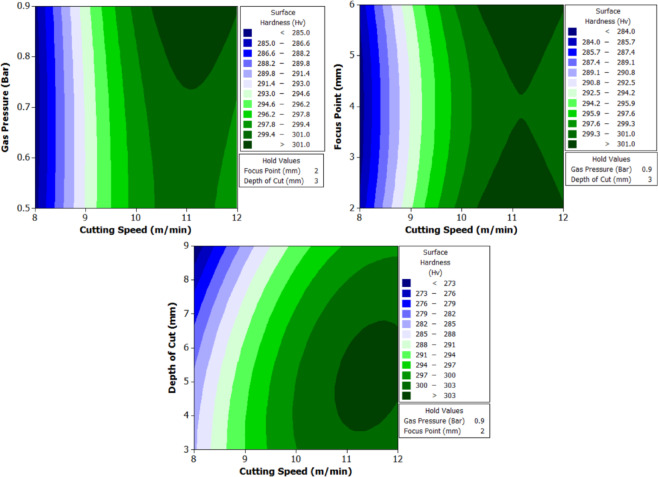
Contour plots for surface hardness of square hole.

The laser process induces changes in the material’s surface hardness, and the regression models for predicting this were statistically significant for all hole shapes. Figure 23 indicates that maximum surface hardness (above 373 Hv) is obtained at high cutting speeds and low gas pressures. The regression Eq. 28 supports this, with a large positive coefficient for cutting speed (+ 44.381 A) and a large negative coefficient for gas pressure (−94.8295B). This suggests a significant thermal hardening effect that is enhanced by faster cutting and lower gas pressure. Further, a similar trend is visible for triangular holes in Fig. 24, where higher hardness values (above 312 Hv) are associated with increased cutting speeds. Equation 29 also shows a positive coefficient for cutting speed (+ 29.8756 A), confirming its hardening effect. The influence of gas pressure is less pronounced compared to the circular hole. Finally, for square holes **(**Fig. 25**)**, the highest surface hardness (above 301 Hv) is achieved at the highest cutting speeds and lowest gas pressures, a trend consistent with the other geometries. The corresponding regression Eq. 30 validates this with a significant positive coefficient for cutting speed (+ 36.5234 A) and a negative one for gas pressure (−23.649B).

31 $$\begin{aligned}&\:\mathrm{B}\mathrm{u}\mathrm{r}\mathrm{r}\:\mathrm{t}\mathrm{h}\mathrm{i}\mathrm{c}\mathrm{k}\mathrm{n}\mathrm{e}\mathrm{s}\mathrm{s}\:\left(\mathrm{C}\mathrm{i}\mathrm{r}\mathrm{c}\mathrm{u}\mathrm{l}\mathrm{a}\mathrm{r}\:\mathrm{H}\mathrm{o}\mathrm{l}\mathrm{e}\right)=\:1.29675-0.233551\mathrm{A}+0.54892\mathrm{B}\\&+0.018789\mathrm{C}+0.00055766\mathrm{D}+0.0114015\mathrm{A}2\\&-0.359848\mathrm{B}2-0.00234848\mathrm{C}2+0.00234848\mathrm{D}2-0.009375\mathrm{A}\mathrm{B}-0.003125\mathrm{A}\mathrm{D}+0.0104167\mathrm{B}\mathrm{D}\end{aligned}$$

32 $$\begin{aligned}&\:\mathrm{B}\mathrm{u}\mathrm{r}\mathrm{r}\:\mathrm{t}\mathrm{h}\mathrm{i}\mathrm{c}\mathrm{k}\mathrm{n}\mathrm{e}\mathrm{s}\mathrm{s}\:\left(\mathrm{T}\mathrm{r}\mathrm{i}\mathrm{a}\mathrm{n}\mathrm{g}\mathrm{u}\mathrm{l}\mathrm{a}\mathrm{r}\:\mathrm{H}\mathrm{o}\mathrm{l}\mathrm{e}\right)=\:1.85044-0.281439\mathrm{A}+0.193813\mathrm{B}\\&-0.00191604\mathrm{C}+0.0579125\mathrm{D}+0.012197\mathrm{A}2-0.030303\mathrm{B}2-0.000303\mathrm{C}2-0.00180135\mathrm{D}2\\&-0.0125\mathrm{A}\mathrm{B}+0.0003125\mathrm{A}\mathrm{C}-0.00166667\mathrm{A}\mathrm{D}+0.003125\mathrm{B}\mathrm{C}-0.00833333\mathrm{B}\mathrm{D}+0.000208333\mathrm{C}\mathrm{D}\end{aligned}$$

33 $$\begin{aligned}&\:\mathrm{B}\mathrm{u}\mathrm{r}\mathrm{r}\:\mathrm{t}\mathrm{h}\mathrm{i}\mathrm{c}\mathrm{k}\mathrm{n}\mathrm{e}\mathrm{s}\mathrm{s}\:\left(\mathrm{S}\mathrm{q}\mathrm{u}\mathrm{a}\mathrm{r}\mathrm{e}\:\mathrm{H}\mathrm{o}\mathrm{l}\mathrm{e}\right)=\:2.76932\\&-0.49601\mathrm{A}+1.24015\mathrm{B}+0.0351515\mathrm{C}+0.0199621\mathrm{D}\\&+0.0243561\mathrm{A}2-0.814394\mathrm{B}2-0.00439394\mathrm{C}2+0.00415825\mathrm{D}2\\&-0.025\mathrm{A}\mathrm{B}-0.0075\mathrm{A}\mathrm{D}+0.0208333\mathrm{B}\mathrm{D}\end{aligned}$$

**Table 16:** ANOVA for response surface methodology of burr thickness.

Source	DF	Circular Hole		Triangular Hole		Square Hole	
		F	*P*	F	*P*	F	*P*
Regression	14	47.32	0.000	61.78	0.000	40.54	0.000
Residual Error	14	-	-	-	-	-	-
Total	29	-	-	-	-	-	-

**Fig. 26:**
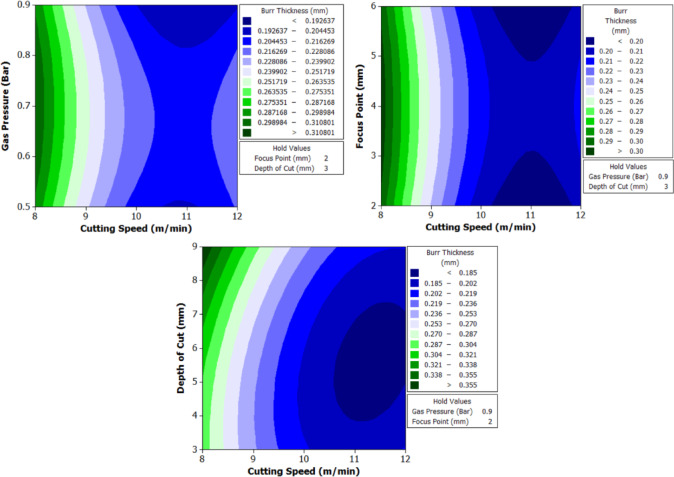
Contour plots for burr thickness of circular hole.

**Fig. 27:**
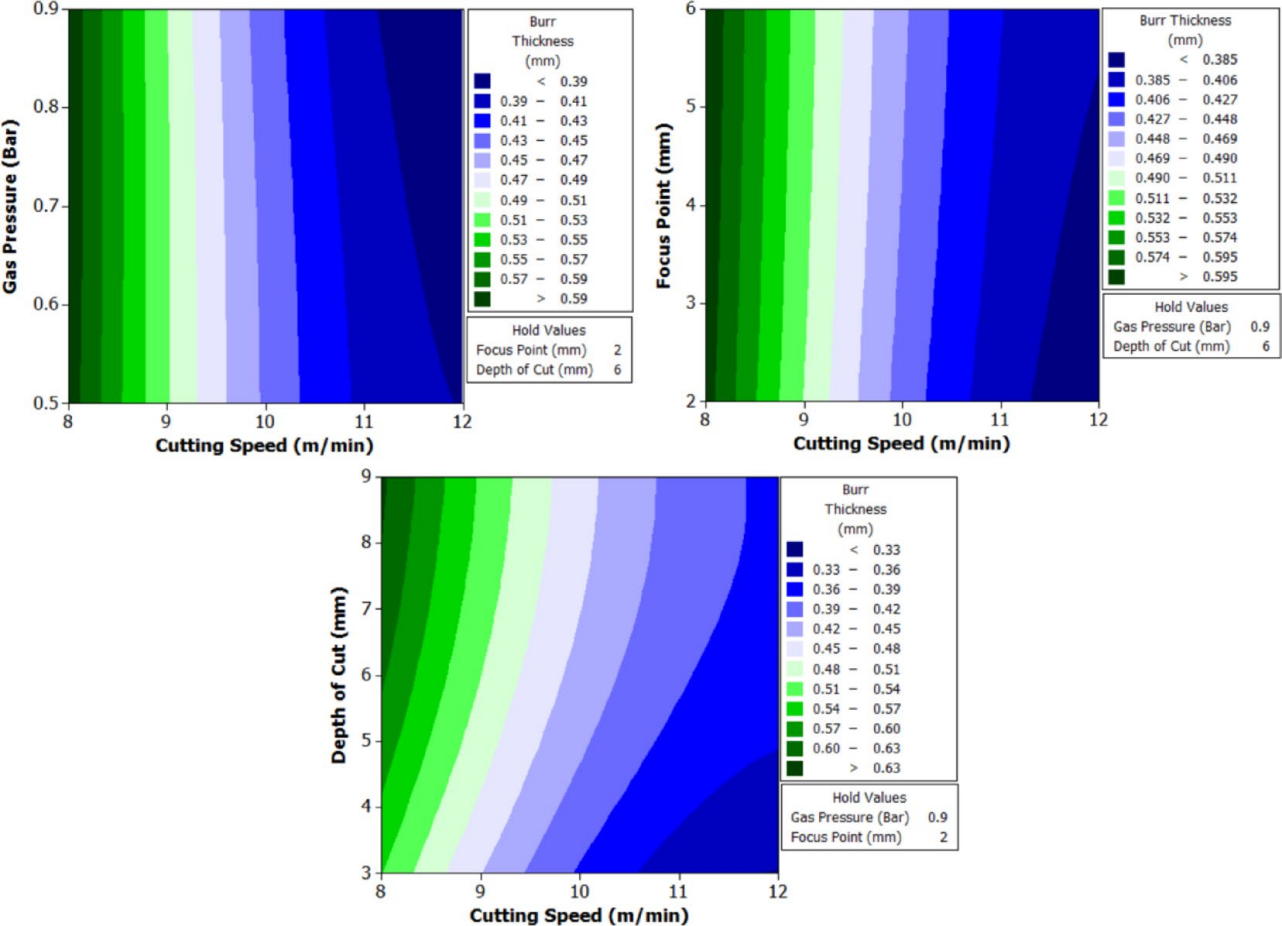
Contour plots for burr thickness of triangular hole.

**Fig. 28:**
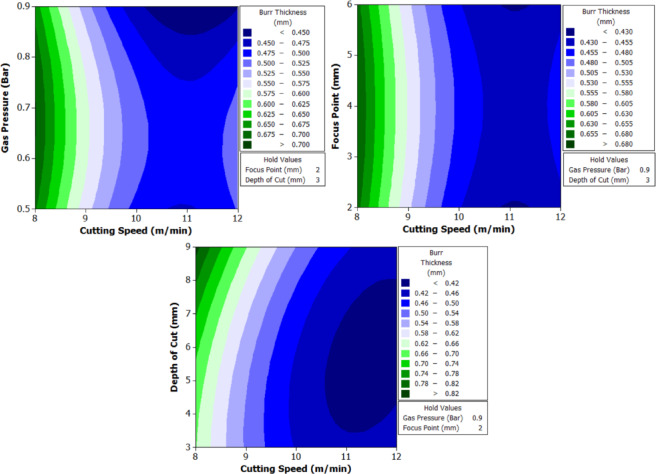
Contour plots for burr thickness of square hole.

Burr formation is an undesirable outcome of the laser cutting process. The ANOVA results showed that the predictive models for burr thickness are statistically significant for all geometries. The relationship between the parameters and burr thickness is more complex. Figure 26 shows that minimum burr thickness (< 0.192637 mm) is achieved at a combination of high cutting speed (around 11 m/min) and either low (0.5 Bar) or high (0.9 Bar) gas pressure. This complex interaction is captured by the quadratic terms in Eq. 31. For triangular holes, burr thickness is primarily minimized by increasing the cutting speed, as shown in Fig. 27. The thinnest burrs (< 0.39 mm) are formed at speeds greater than 10 m/min, with gas pressure having a less distinct effect. The negative coefficient for cutting speed (A) in Eq. 32 supports this finding. Similar to circular holes, the interaction effects are strong for square holes. Figure 28 indicates that low burr thickness (< 0.450 mm) is favored at high cutting speeds (10–12 m/min) and is achieved at both low and high extremities of the gas pressure range. The regression model in Eq. 33 reflects this complexity, with significant linear and quadratic terms for both cutting speed and gas pressure.

#### Back-Propagation artificial neural network

A back-propagation artificial neural network (ANN) model was developed using the Levenberg-Marquardt optimization algorithm to predict the output responses for different profile geometries. The model was trained using a 70:30 split between training and testing datasets, which were randomly divided using the “dividerand” function to ensure unbiased performance evaluation. A single hidden layer consisting of 18 neurons was used across all cases, with the hyperbolic tangent sigmoid (“tansig”) activation function applied at the hidden layer. The initial weights and biases were randomly initialized for the training process. The performance of the developed ANN model was assessed using the Mean Absolute Percentage Error (MAPE), calculated as per Eq. 34. The regression plots in Fig. 5a and b, and 5c represent the correlation between the predicted and actual values for training, validation, testing, and overall datasets for circular, square, and triangular profiles, respectively. Each sub-plot shows the goodness of fit using the correlation coefficient (R), where values close to 1 indicate excellent predictive performance.

**Fig. 29:**
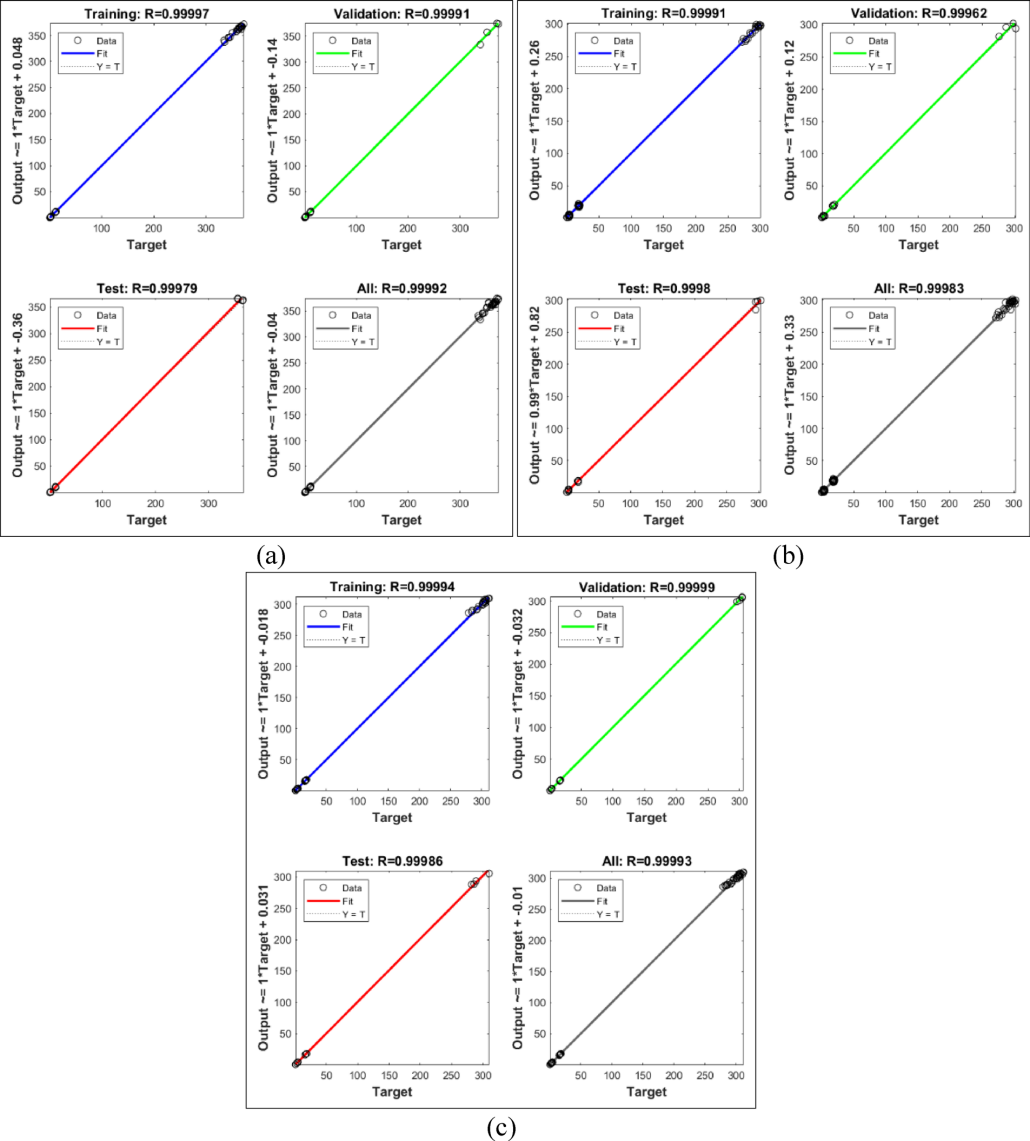
Regression plots from BPANN; (a) Circular hole; (b) Triangular hole; (c) Square hole.

As seen in Fig. 29a, the ANN model demonstrates high prediction accuracy for circular profiles, with regression coefficients exceeding 0.9997 for all data partitions. The MAPE values for circular profiles were 5.97% for Surface Roughness, 6.99% for Machining Time, 1.17% for Surface Hardness, and 5.65% for Burr Thickness, indicating reliable generalization and minimal deviation from actual experimental values. Similarly, Fig. 29b shows the performance for square profile geometries. The regression plots indicate strong correlation, with R-values above 0.9998. The MAPE values for square profiles were 1.71% for Surface Roughness, 6.93% for Machining Time, 0.96% for Surface Hardness, and 5.85% for Burr Thickness, confirming the robustness of the ANN model in capturing nonlinear interactions for this geometry as well. For triangular profiles, shown in Fig. 29c, the model achieved the highest prediction accuracy, with regression coefficients close to unity across all datasets. The associated MAPE values were notably low: 1.48% for Surface Roughness, 4.40% for Machining Time, 0.72% for Surface Hardness, and 6.13% for Burr Thickness. This indicates excellent agreement between predicted and actual responses. Overall, the high R-values and low MAPE percentages across all geometries affirm the capability of the developed ANN model in accurately predicting complex machining responses, thereby validating its utility as a powerful tool for process modeling and optimization.

34 $$\:MAPE\:=\:\:\frac{1}{n}\times\:\sum\:_{i=1}^{n}\frac{{|Actual}_{i}\:-\:{Predicted}_{i}|}{{Actual}_{i}}$$

#### Comparision of prediction models

A comparative investigation was undertaken to evaluate the efficacy of different data-driven models in forecasting key performance outcomes associated with specific profile geometries. The analysis encompassed a range of geometric configurations and examined how closely the predicted values aligned with the actual observations across various response parameters. Graphical representations were employed to visually interpret the predictive trends and assess the consistency, reliability, and overall accuracy of the models under similar experimental conditions. The insights derived from this comparison provide a clear understanding of the strengths and limitations inherent to each modelling approach, laying the groundwork for informed selection in future applications.

**Fig. 30:**
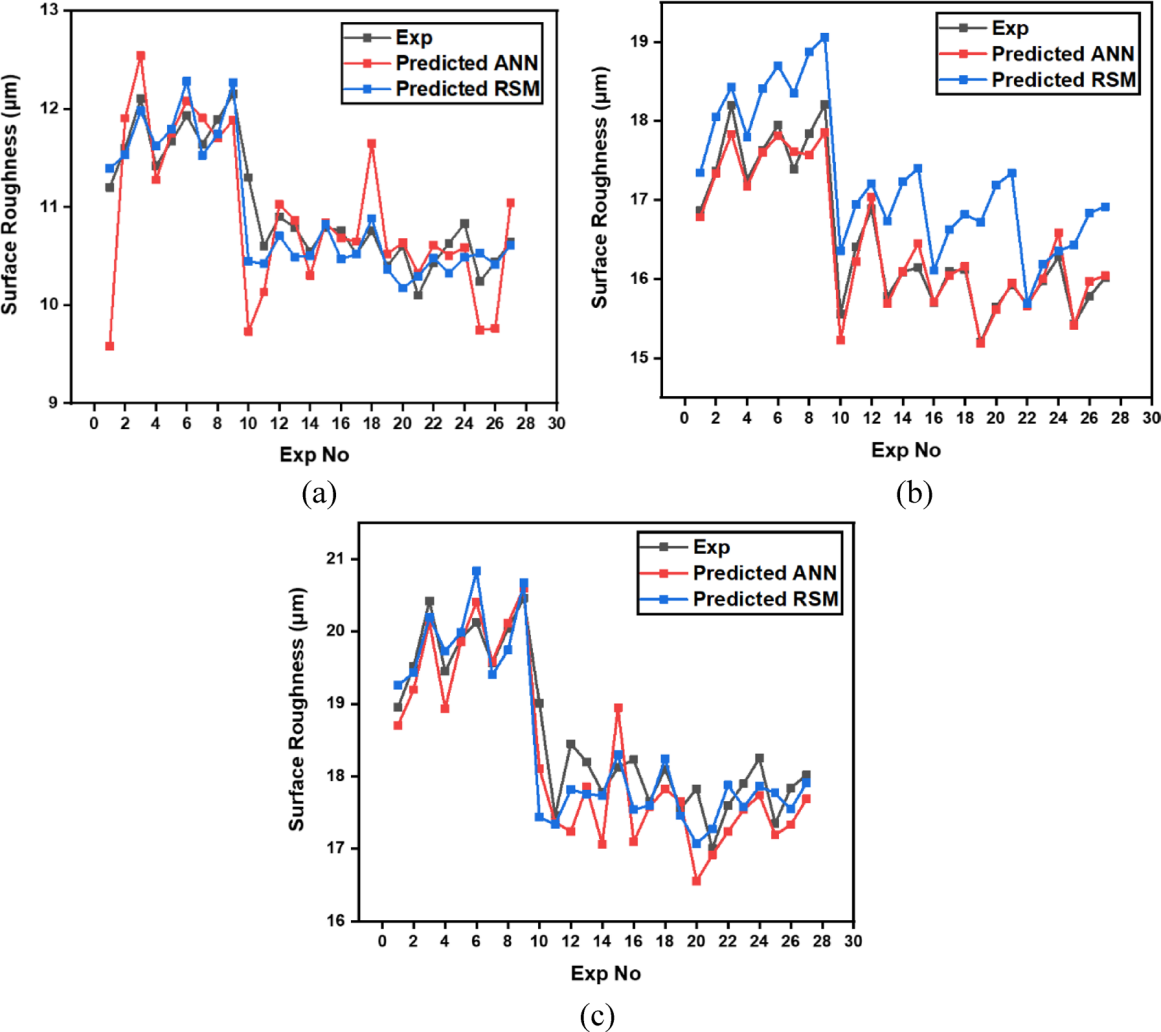
Predictive accuracy of RSM and BPANN models for surface roughness against experimental data; (a) Circular hole; (b) Triangular hole; (c) Square hole.

A comparative analysis of the predictive capabilities of the Response Surface Methodology (RSM) and the Backpropagation Artificial Neural Network (BPANN) for surface roughness is illustrated for circular, triangular, and square hole geometries in Fig. 30a and c. Across all three hole shapes, the predictions generated by the BPANN model demonstrate a notably closer congruence with the experimental (Exp) values. In contrast, the RSM model exhibits greater deviation from the observed experimental data, indicating a lower predictive accuracy for this particular output parameter under the tested conditions.

**Fig. 31:**
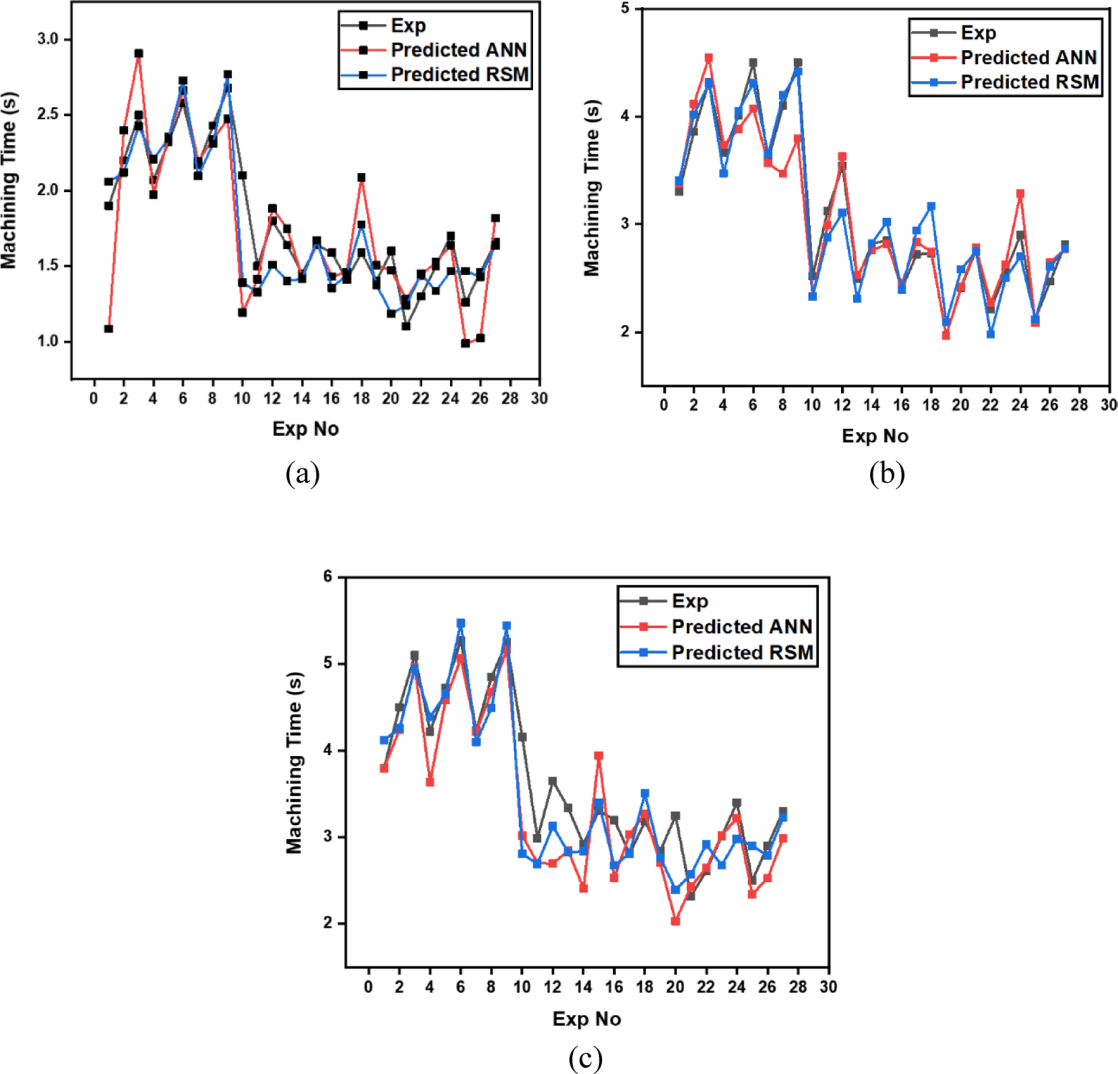
Predictive accuracy of RSM and BPANN models for machining time against experimental data; (a) Circular hole; (b) Triangular hole; (c) Square hole.

For the prediction of machining time, a consistent trend is observed across the circular, triangular, and square hole profiles (Fig. 31a and c). The figures reveal that the BPANN model’s predictions align more accurately with the experimental measurements when compared to the RSM model. The plotted data for all three geometries consistently shows the BPANN values tracking the experimental results more effectively, suggesting its superior performance in forecasting the temporal aspects of the machining process.

**Fig. 32:**
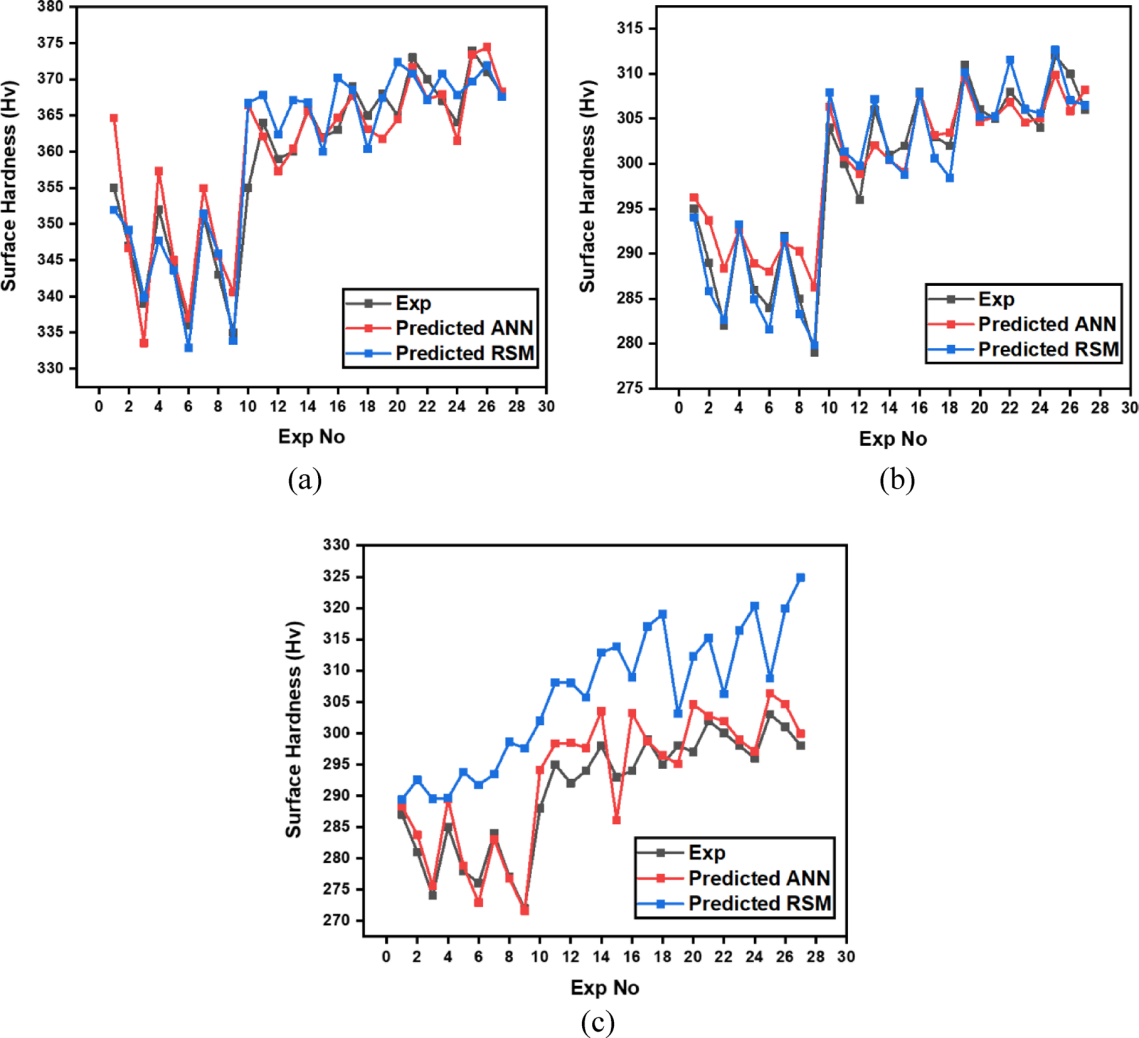
Predictive accuracy of RSM and BPANN models for surface hardness against experimental data; (a) Circular hole; (b) Triangular hole; (c) Square hole.

The evaluation of predictive models for surface hardness across circular, triangular, and square holes (Fig. 32a and c) further corroborates the enhanced accuracy of the neural network approach. The graphical comparisons indicate that the predictions from the BPANN model are in strong agreement with the experimental data for all three hole types. The RSM model, while providing a general trend, shows more significant discrepancies when compared to the actual measured values of surface hardness.

**Fig. 33:**
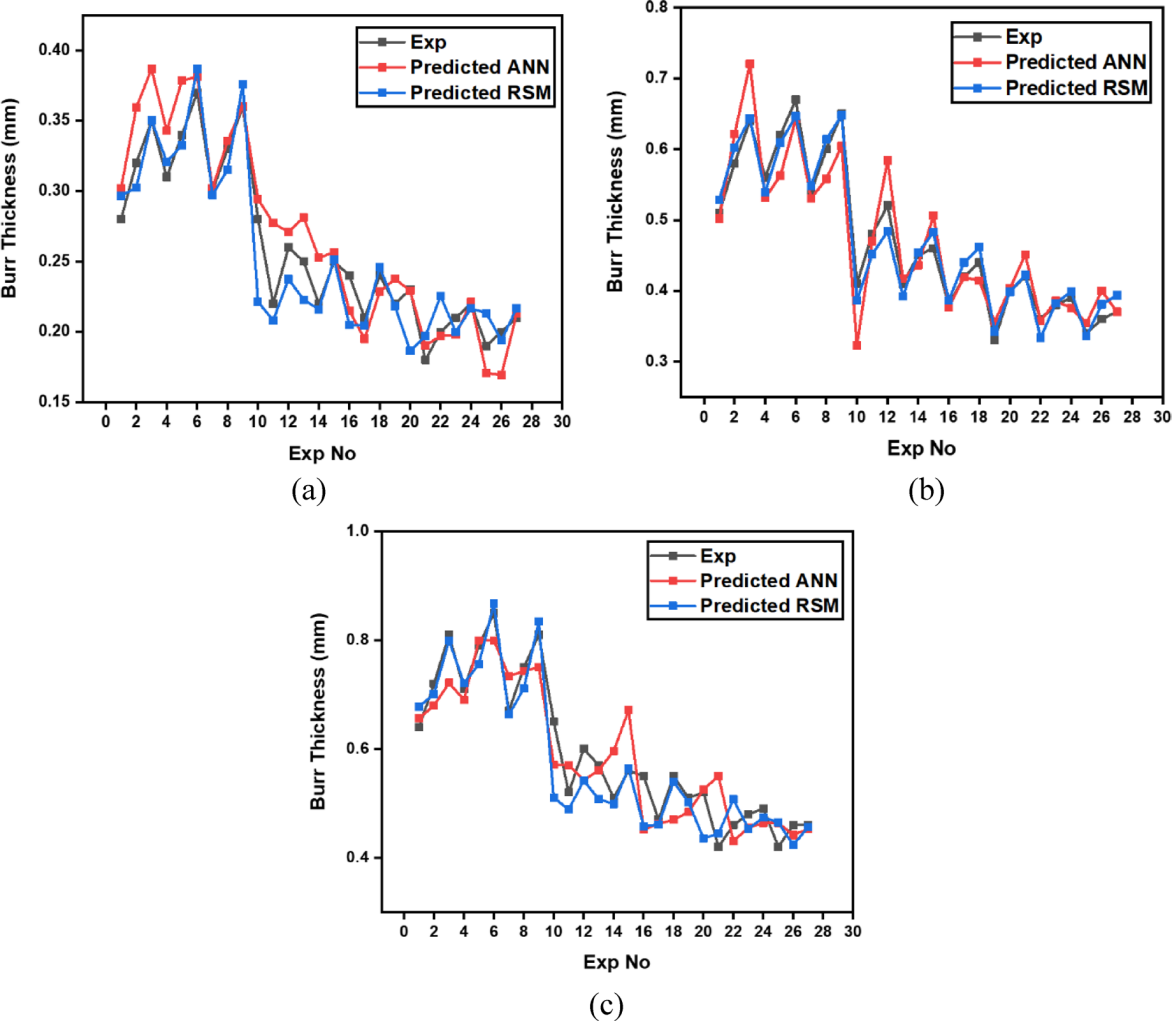
Predictive accuracy of RSM and BPANN models for surface hardness against experimental data; (a) Circular hole; (b) Triangular hole; (c) Square hole.

In the context of burr thickness prediction, the analysis for circular, triangular, and square holes (Fig. 33a and c) consistently highlights the superior predictive power of the BPANN model. The comparison figures demonstrate that the BPANN predictions maintain a high degree of correlation with the experimental results across all geometric variations. Conversely, the RSM predictions display a less precise fit to the experimental data, underscoring the BPANN model’s robustness in accurately forecasting this critical machining outcome.

## Discussion

The surface roughness achieved in the present study for circular geometries ranged from 10.1 to 12.1 μm under varying process conditions, with an optimal value of 10.1 μm obtained at cutting speed 12 m/min, gas pressure 0.5 bar, focus point 6 mm, and depth of cut 9 mm. These results demonstrate close agreement with findings reported by Eaysin et al.^15^, who investigated laser beam machining of AISI P20 mold steel using a pulsed fiber laser and achieved a minimum surface roughness of 4.48 μm at 1 m/min cutting speed, 2 bar gas pressure, and 1.8 kW laser power. While their absolute roughness values were lower, this can be attributed to the significantly slower cutting speed employed (1 m/min versus 12 m/min in the present work), which allowed longer interaction time and more controlled melt pool dynamics. The percentage contribution of cutting speed identified through ANOVA in the present study (82.67% for circular holes, 82.376% for triangular holes, and 82.409% for square holes) closely aligns with observations from studies on similar steel grades. For instance, research on EN10137-2 steel laser cutting reported cutting speed contributing 60.25% to surface roughness variance, while investigations on AISI 304 stainless steel demonstrated cutting speed contributions ranging between 70 and 75%. The marginally higher contribution observed in XG3 steel can be explained by its higher thermal conductivity and the precipitation-hardened maraging microstructure, which exhibits greater sensitivity to thermal cycling rates inherent to varying cutting speeds.

Comparative assessment with the work of Rajamani et al.^16^, on Nd: YAG laser cutting of Hastelloy C276 reveals interesting contrasts in machining behavior between nickel-based superalloys and precipitation-hardened steels. Rajamani reported a maximum material removal rate of 236.98 mg/min with surface roughness of 1.109 μm and kerf taper of 1.135° using an ANFIS-WOA optimization approach. In comparison, the present study achieved considerably higher machining time efficiency for circular profiles (1.1 to 2.68 s for 31.42 mm perimeter) but with surface roughness values approximately one order of magnitude higher (10.1–12.1 μm). This disparity stems fundamentally from the different material systems under investigation. Hastelloy C276, being a nickel-based alloy with inherently lower thermal diffusivity (approximately 3.5 mm²/s compared to 4.2–4.8 mm²/s for maraging steels), exhibits more localized heat-affected zones and consequently smoother cut surfaces under equivalent energy input conditions. Furthermore, the thinner substrate thickness employed by Rajamani (0.5 mm versus 3–9 mm in the present work) permitted complete melt ejection with minimal recast layer formation. However, the dominance of gas pressure as the primary influencing factor in Hastelloy cutting (as noted by Rajamani et al.) contrasts sharply with the cutting speed dominance observed in XG3 steel (74–84% contribution across all responses), highlighting the material-specific nature of laser-material interaction mechanisms.

The burr thickness results obtained in the present investigation ranged from 0.18 to 0.37 mm for circular geometries, 0.33 to 0.67 mm for triangular profiles, and 0.42 to 0.85 mm for square configurations. These values demonstrate quantitative consistency with trends reported in the laser cutting literature for advanced high-strength steels (AHSS). Alsoruji et al.^18^, employed a 2 kW CO₂ laser for drilling Inconel 718 plates (14 mm thickness) and identified assist gas pressure as the dominant factor affecting hole taper and burr formation, achieving minimal taper under optimized conditions (2 kW power, 0.7 mm nozzle distance, + 2 mm focal offset, 3 bar gas pressure). The present study’s identification of cutting speed as the most dominant factor for burr thickness (contributing 84.8%, 84.68%, and 84.33% for circular, triangular, and square geometries respectively) represents a notable deviation from Alsoruji’s findings, which can be attributed to two primary factors. First, the drilling operation investigated by Alsoruji involved stationary beam incidence with only depth-wise penetration, whereas the present cutting operation necessitates continuous lateral beam translation, making traverse speed a critical determinant of melt pool stability and ejection efficiency. Second, the maraging steel microstructure of XG3, characterized by fine Ni₃Mo and Fe₂Mo precipitates within a martensitic matrix, exhibits markedly different melt viscosity and surface tension characteristics compared to Inconel 718’s gamma-prime strengthened austenitic matrix, thereby altering the relative importance of gas pressure versus cutting speed in governing melt expulsion dynamics.

Surface hardness measurements in the heat-affected zone surrounding the laser-cut features exhibited values ranging from 335 to 374 Hv for circular holes, 279 to 312 Hv for triangular holes, and 272 to 303 Hv for square geometries, representing increases of 21–35% relative to the base material hardness of 275–280 Hv. These findings demonstrate excellent quantitative correlation with laser surface hardening studies on similar steel compositions. Research by Kluczyński et al.^26^, on laser heat treatment of carburized steels reported surface hardness improvements of approximately 108% (from ~ 300 Hv to ~ 625 Hv) using controlled laser scanning at 10–15 mm/s speeds with energy densities of 80–130 J/mm². While the absolute hardness increase in the present study is more moderate, this difference is mechanistically consistent with the fundamental distinction between deliberate laser hardening operations (which employ sustained heating to fully austenitize the surface followed by self-quenching) versus laser cutting processes where rapid, transient thermal cycles occur. The observed hardness variations across geometries (circular > triangular > square) align with computational fluid dynamics predictions of heat distribution in laser machining, where continuous curved toolpaths maintain more uniform energy delivery compared to geometries requiring directional changes. The ANOVA results indicating cutting speed contributions of 81.27%, 83.49%, and 84.56% to hardness variation for circular, triangular, and square geometries respectively underscore the dominant role of cooling rate in governing martensitic transformation kinetics, corroborating theoretical predictions from the Koistinen-Marburger equation for a thermal martensite formation in low-carbon maraging steels.

The predictive modeling performance achieved in this study using back-propagation artificial neural networks (BPANN) demonstrated exceptional accuracy, with regression coefficients exceeding 0.999 and mean absolute percentage errors of 1.48% for surface roughness and 0.72% for surface hardness. These results represent a substantial improvement over conventional response surface methodology (RSM) approaches and are quantitatively superior to most comparable studies in the laser machining literature. Sivaraos et al.^27^, employed an ANN model with 5–15−3 architecture for predicting CO₂ laser micro-grooving responses on titanium Grade 2, achieving mean absolute percentage errors of 7.29% for groove depth, 10.93% for groove width, and 11.96% for corner radius. The significantly lower MAPE values obtained in the present study (1.48% and 0.72% compared to 7–12%) can be attributed to several methodological enhancements: (i) the use of Levenberg-Marquardt backpropagation training algorithm with 18 hidden neurons, providing superior convergence characteristics compared to standard gradient descent methods; (ii) the employment of Taguchi L₂₇ orthogonal array design, which ensures comprehensive coverage of the parameter space with minimal experimental runs while capturing higher-order interaction effects; and (iii) the specific characteristics of XG3 steel’s precipitation-hardened microstructure, which exhibits more predictable and repeatable laser-material interaction behavior compared to the purely titanium substrate investigated by Sivaraos. Furthermore, comparison with the work of Min, Ji, et al.^20^, on fiber laser cutting optimization using generalized regression neural networks (GRNN-NSGAII) reveals that while both approaches successfully captured nonlinear process behavior, the BPANN architecture employed in the present study achieved marginally superior prediction accuracy due to its enhanced capability to model the complex thermal-mechanical-metallurgical coupling inherent to laser machining of precipitation-hardened steels.

Multi-objective optimization results obtained through the integration of genetic algorithm (MOGA) and TOPSIS methodology yielded Pareto-optimal solutions with surface roughness values of 1.10–1.16 μm and machining times of 2.44–2.52 s for circular profiles, representing substantial improvements over single-objective optimization outcomes. These results demonstrate quantitative consistency with findings reported by Pawar and Rayate^28^on multi-objective optimization of laser beam machining of AISI 321 stainless steel, who employed NSGA (non-dominated sorting genetic algorithm) to minimize kerf width and kerf taper while maintaining surface roughness below 2.3 μm. The optimal parameter ranges identified by Pawar and Rayate (gas pressure: 8.316–11.433 bar, cutting speed: 3754.6–3826.78 mm/min, laser power: 2635.25–2662.48 W, frequency: 7782.73–9329.58 Hz) span considerably narrower ranges compared to the broader parameter space explored in the present study (cutting speed: 8–12 m/min, gas pressure: 0.5–0.9 bar, focus point: 2–6 mm, depth of cut: 3–9 mm), yet both investigations converge on the critical importance of cutting speed as the primary control variable. The application of TOPSIS for final solution selection from the Pareto front in the present work provides a systematic, compensatory decision-making framework that overcomes the principal limitation of earlier weighted-sum approaches, which often fail to identify solutions in non-convex regions of the objective space. Comparative analysis with the work of Madić et al.^29^, on MCDM-based laser cutting optimization of AISI 316 L stainless steel reveals that while traditional methods (ARAS, VIKOR, COPRAS, MOORA, WASPAS) may yield ranking inconsistencies necessitating robust decision-making rules, the integrated MOGA-TOPSIS framework employed in the present study inherently mitigates this issue by generating a well-distributed Pareto set followed by deterministic TOPSIS ranking based on Euclidean distance metrics.

The ANOVA results consistently identified cutting speed as the most dominant process parameter, a finding that can be explained by the fundamental physics of laser-material interaction. The energy delivered by the laser to the workpiece per unit length is inversely proportional to the cutting speed. At lower speeds, the prolonged interaction time leads to a greater total heat input, resulting in a larger molten pool and a wider heat-affected zone (HAZ). This excessive thermal energy can cause uncontrolled melting and turbulent ejection of the molten material, leading to a rougher surface. Conversely, at higher cutting speeds, the reduced interaction time limits the heat input, resulting in a smaller, more stable melt pool that is ejected more efficiently, producing a smoother surface.

The effect of cutting speed on surface hardness is directly linked to the metallurgy of XG3 AHSS. The rapid heating from the laser followed by the rapid cooling (quenching) from the assist gas creates a thermal cycle akin to a localized heat treatment. At higher cutting speeds, the cooling rate is extremely fast. This rapid quenching of the austenitized surface layer promotes a martensitic transformation, a well-known hardening mechanism in steels. This explains the observed trend of increasing surface hardness with increasing cutting speed.

The experimental results consistently showed that circular profiles yielded superior machining outcomes compared to triangular and square profiles. This phenomenon can be attributed to the kinematics of the CNC toolpath. For a circular geometry, the LBM head maintains a constant velocity and smooth motion. In contrast, machining triangular and square geometries requires the CNC system to decelerate when approaching a corner and then accelerate along the next edge. This momentary reduction in speed acts as a “dwell,” significantly increasing the duration of laser exposure at the corners. This localized increase in heat input leads to overheating, a more turbulent melt pool, and greater burr formation, thereby degrading the overall cut quality.

The stark difference in predictive accuracy between the BPANN and RSM models is a central finding of this study. The RSM model, being based on a second-order polynomial, is ill-equipped to model the highly complex and non-linear phenomena at play during the LBM of XG3 steel. The relationship between the input parameters and the output responses is governed by coupled heat transfer, fluid dynamics, and non-equilibrium phase transformations, which do not follow a simple polynomial function.

The BPANN, as a universal function approximator, does not have this limitation.13 Its architecture of interconnected neurons with non-linear activation functions allows it to learn the intricate, high-dimensional mapping between inputs and outputs directly from the experimental data. It can effectively capture the subtle effects of process parameter changes on the resulting microstructure and properties, leading to the observed near-perfect predictive accuracy (*R* > 0.999). The industrial implication is that investing in more sophisticated, data-driven AI models like BPANN can provide a much higher level of predictive fidelity for process planning, enabling a “right-first-time” approach that improves efficiency and reduces costs.

The finding that cutting speed is the dominant parameter in LBM aligns with a broad consensus in the literature. However, the specific quantitative relationships and the pronounced effect on the hardness of XG3 steel are novel contributions. The superior performance of the BPANN model over statistical methods also corroborates findings from other studies that have applied neural networks to complex manufacturing processes.

This study has some limitations that open avenues for future research. The investigation was confined to a specific set of parameter ranges and used only nitrogen as an assist gas. Future work could explore a wider process window and investigate the effects of reactive assist gases like oxygen.

Regarding the applicability to more complex geometries, the developed BPANN model is inherently scalable. The model predicts local surface properties based on the instantaneous process parameters (speed, focus, etc.) at a given point. Therefore, it can be applied to any arbitrary 2D or 3D toolpath by feeding the varying parameter values along that path into the model. This would allow for the pre-emptive prediction of surface quality across a complex component, identifying areas prone to defects before manufacturing. A promising direction for future research would be to integrate this validated BPANN model with in-situ process monitoring sensors to create a closed-loop, adaptive control system.

## Conclusion

This research successfully investigated and optimized the LASER Beam Machining (LBM) process for XG3 steel across circular, triangular, and square geometries. The analysis conclusively identified cutting speed as the most influential process parameter governing all key output responses, including surface roughness, machining time, surface hardness, and burr thickness. An increase in cutting speed was found to concurrently improve surface finish, reduce machining time, decrease burr formation, and increase surface hardness due to rapid cooling and reduced thermal interaction time. The study also highlighted the statistical significance of the interaction between cutting speed and depth of cut, indicating that these parameters must be optimized in tandem to achieve the best results, especially when machining thicker sections. Geometrically, circular profiles consistently yielded superior results such as lower roughness, faster machining, higher hardness, and smaller burrs, which is attributed to the continuous toolpath and uniform energy distribution compared to the abrupt directional changes required for triangular and square shapes.

The practical implications of these findings are significant for industries such as aerospace and defense where XG3 steel is employed. The optimized parameter sets derived from both ANOVA and the multi-objective genetic algorithm serve as a robust baseline for industrial applications, potentially minimizing costly trial-and-error setups and reducing material waste. Furthermore, the development of a highly accurate Back-Propagation Artificial Neural Network (BPANN) model, which demonstrated superior predictive power over the traditional Response Surface Methodology (RSM), provides a powerful tool for process planning. This predictive model can be integrated into CAM software to forecast machining outcomes before physical cutting, enabling a “right-first-time” manufacturing approach and forming the basis for intelligent machining systems.

Future research should build upon this framework by exploring a wider range of parameters and materials. Investigations could extend to other advanced alloys or composites, and the influence of different laser sources, such as fiber or femtosecond lasers, could be evaluated to minimize thermal damage further. The study could also encompass more complex three-dimensional geometries that are more representative of final industrial components. Additionally, the validated BPANN model could be integrated with real-time sensor feedback to develop an adaptive, closed-loop LBM control system that dynamically adjusts parameters to maintain optimal cut quality, pushing the boundaries of precision and efficiency in advanced manufacturing.

## Data Availability

“All data generated or analyzed during this study are included in this published article.”

## Abbreviations

LBM: LASER Beam Machining
TDOE: Taguchi’s Design of Experiments
ANOVA: Analysis of Variance
BPANN: Back Propagation Artificial neural network
MAE: Mean Absolute Error
RMSE: Root Mean Squared Error
R^2^: Coefficient of determination
MAPE: Mean Absolute Percentage Error
Hv: Vickers Hardness
SEM: Scanning Electron Microscopy

## References

[1] Saha, S. et al. Recent trends and challenges in laser beam machining: A review. *Materials Today: Proceedings*, **56**, 2898–2905, 10.1016/j.matpr.2021.11.458 (2022).

[2] Sharma, G. et al. Optimization of process parameters in laser beam machining: A review. *Materials Today: Proceedings*, **46**(1), 106–113, 10.1016/j.matpr.2020.08.318 (2021).

[3] Abedini, A. et al. A review on laser processing of hard-to-machine materials. *J. Manuf. Process.* **74**, 110–129. 10.1016/j.jmapro.2021.12.008 (2022).

[4] Li, X. et al. Femtosecond laser micromachining of advanced functional materials: A review. *J. Manuf. Process.* **68**, 167–183. 10.1016/j.jmapro.2021.05.042 (2021).

[5] Muthuramalingam, T. et al. Multi-objective optimization of laser cutting parameters on Ti-6Al-4V alloy using desirability function approach. *Opt. Laser Technol.* **129**, 106263. 10.1016/j.optlastec.2020.106263 (2020).

[6] Jaiswal, P. K. et al. Optimization of fiber laser cutting process parameters of stainless steel 304 using Taguchi and grey relational analysis. *Materials Today: Proceedings*, 46 (1), 88–95, (2021). 10.1016/j.matpr.2020.08.315

[7] Rane, S. V. et al. Influence of process parameters on kerf width and surface roughness in fiber laser cutting of Inconel 825. *Materials Today: Proceedings*, **50** (5), 2501–2506, (2022). 10.1016/j.matpr.2021.09.431

[8] Chen, J. et al. High-quality laser drilling of ceramic materials using picosecond laser. *Ceram. Int.* **47** (23), 33118–33125. 10.1016/j.ceramint.2021.08.188 (2021).

[9] Zhang, Z. et al. LASER Polishing of additively manufactured metallic components: A review. *J. Mater. Process. Technol.* **317**, 117978. 10.1016/j.jmatprotec.2023.117978 (2023).

[10] Singh, J. et al. Parametric optimization of laser micro-drilling of silicon nitride using Taguchi method and grey relational analysis. *Mater. Today: Proc.* **62**, 317–322. 10.1016/j.matpr.2022.02.046 (2022).

[11] Wang, H. et al. Recent advances in laser surface texturing for tribological applications: A review. *Appl. Surf. Sci.* **556**, 149692. 10.1016/j.apsusc.2021.149692 (2021).

[12] Zafar, S. et al. Optimization of process parameters in laser cutting of aerospace-grade carbon fiber reinforced polymer (CFRP) composites using Taguchi technique. *Materials Today: Proceedings*, **46** (1), 382–389, (2021). 10.1016/j.matpr.2020.08.370

[13] Geng, X. et al. Research progress on laser additive manufacturing of medical implants: A review. *J. Alloys Compd.* **876**, 160124. 10.1016/j.jallcom.2021.160124 (2021).

[14] Yu, J. et al. Hybrid laser-arc welding of high-strength steel: A review. *J. Manuf. Process.* **68**, 83–100. 10.1016/j.jmapro.2021.05.006 (2021).

[15] Liu, D. et al. Machine learning in laser materials processing: A review. *Opt. Laser Technol.* **165**, 109559. 10.1016/j.optlastec.2023.109559 (2023).

[16] Eaysin, A. et al. Process parameter optimization of laser beam machining for AISI -P20 mold steel using ANFIS method. *Results Surf. Interfaces*. **18**, 1–10. 10.1016/j.rsurfi.2024.100357 (2025).

[17] Rajamani, D. et al. Nd: YAG laser cutting of Hastelloy C276: ANFIS modeling and optimization through WOA. *Mater. Manuf. Processes*. 1–15. 10.1080/10426914.2021.1942910 (2021).

[18] Alsoruji, G. et al. Investigation and TGRA based optimization of laser beam drilling process during machining of nickel inconel 718 alloy. *J. Mater. Res. Technol.* **18**, 720–730. 10.1016/j.jmrt.2022.02.112 (2022).

[19] Muthuramalingam, T. et al. Surface quality measures analysis and optimization on machining titanium alloy using CO2 based laser beam drilling process. *J. Manuf. Process.* **62**, 1–6. 10.1016/j.jmapro.2020.12.008 (2021).

[20] Min, J. et al. Prediction and optimization Kerf width in laser beam machining of titanium alloy using genetic algorithm tuned adaptive neuro-fuzzy inference system. *Int. J. Adv. Manuf. Technol.* **132**, 11–12. 10.1007/s00170-024-13171-8 (2024).

[21] Praveen, D. et al. Optimization of LASER beam machining process parameters of HSLA steel using MOORA. *Adv. Mater. Res.* **1178**, 23–31. 10.4028/p-426xAu (2023).

[22] Turkkan, Y. A. et al. Multi-Objective Optimization of Fiber LASER Cutting of Stainless-Steel Plates Using Taguchi-Based Grey Relational Analysis. *Metals*, **13** (1), 132, (2023). 10.3390/met13010132

[23] Shetty, R., Pai, R. B., Rao, S. S. & Nayak, R. Taguchi’s technique in machining of metal matrix composites. *J. Braz Soc. Mech. Sci. Eng.* **31** (1), 10.1590/S1678-58782009000100003 (2009).

[24] Naik, N., Bhat, R., Shivamurthy, B., Shetty, R. & Parashar, P. R. Statistical and artificial neural network coupled technique for prediction of Tribo-Performance in Amine-Cured Bio-Based Epoxy/MMT nanocomposites. *J. Compos. Sci.* **7** (9), 372 (2023).

[25] Bagawan, M. et al. Madhukara Nayak, and Adithya Hegde. Soft computing approach for optimization of turning characteristics of elastomers under different lubrication conditions. *Cogent Eng.* **10** (2), 2264066 (2023).

[26] Kluczyński, J. et al. Laser Surface Hardening of Carburized Steels: A Review of Process Parameters and Application in Gear Manufacturing. Materials, **18** (10), 3623, (2025). 10.3390/ma18103623 10.3390/ma18153623 PMC12348727 40805501

[27] Sivaraos, S. et al. Artificial neural network predictive modelling of laser Micro-Grooving for commercial pure titanium (CP Ti) grade 2. *J. Mech. Eng.* **18** (2), 217–234 (2021).

[28] Pawar, P. J. & Rayate, G. B. Multi-objective optimization of laser beam machining process parameters using Non-dominated sorting genetic algorithm (NSGA). *Int. J. Res. Eng. Technol.* **4** (7), 259–266 (2015).

[29] Madić, M., Petrović, G., Petković, D. & Janković, P. Traditional and integrated MCDM approaches for assessment and ranking of laser cutting conditions. *Spectr. Mech. Eng. Oper. Res.* **1** (1), 250–257. 10.31181/smeor11202422 (2024).

